# Supplementary material for: OXTRHigh stroma fibroblasts control the invasion pattern of oral squamous cell carcinoma via ERK5 signaling
Source: Nat Commun. 2022 Aug 31;13:5124. doi: 10.1038/s41467-022-32787-y (PMC9433374; doi:10.1038/s41467-022-32787-y)
Supplement: Supplementary file 1 — Supplementary Information [file 41467_2022_32787_MOESM1_ESM.pdf]

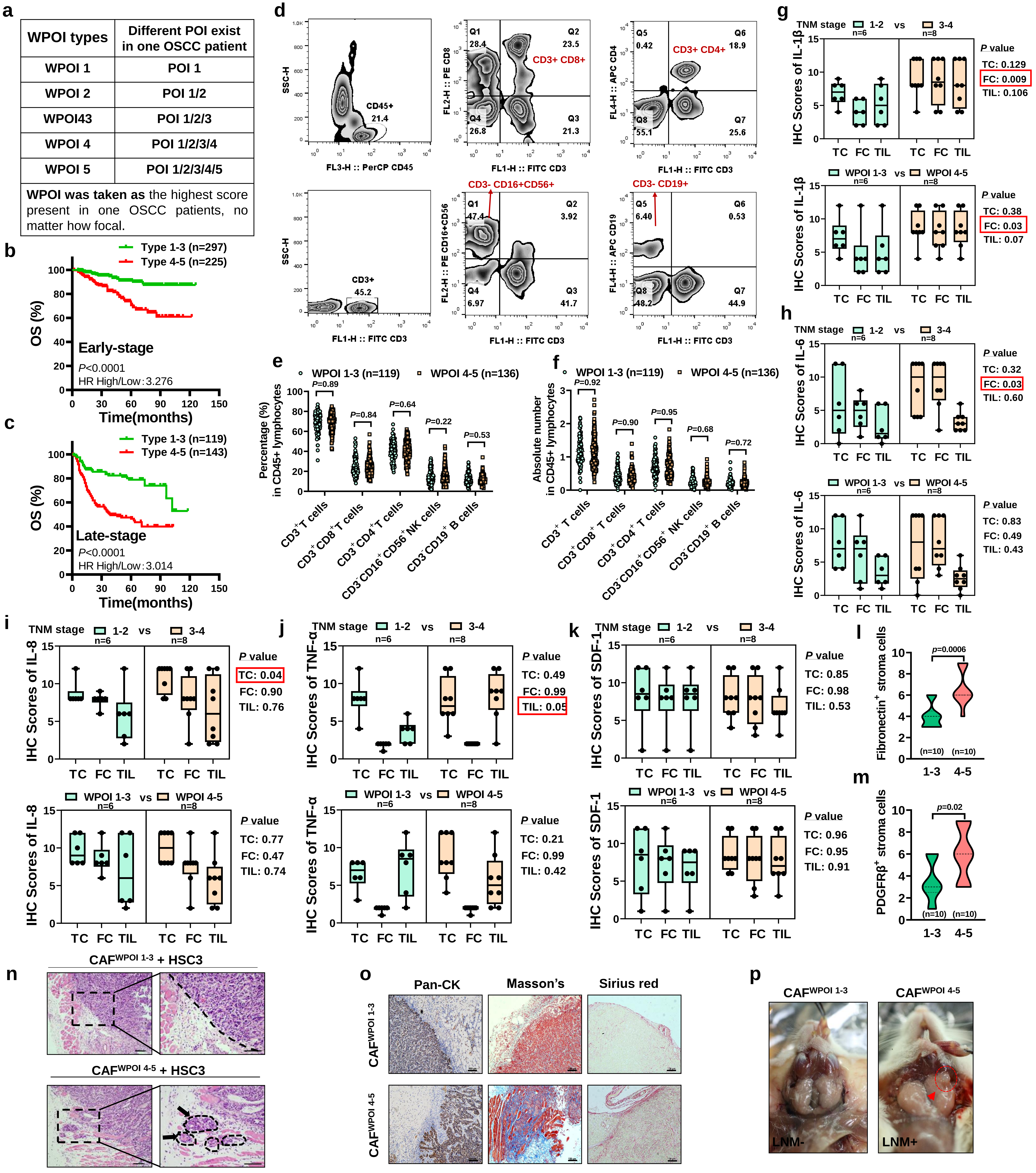

WPOI types	Different POI exist in one OSCC patient
WPOI 1	POI 1
WPOI 2	POI 1/2
WPOI43	POI 1/2/3
WPOI 4	POI 1/2/3/4
WPOI 5	POI 1/2/3/4/5
**WPOI was taken as** the highest score present in one OSCC patients, no matter how focal.	

Supplementary Figure 1. The Role of stroma CAFs in WPOI 4-5. (a) Tables indicated the association between different POI and WPOI for each OSCC patients. (b, c) Overall survival was assessed in patients with WPOI 1-3 (n=416) and 4-5 (n=368) by Log-rank (Mantel-Cox) test. (d-f) The ratio and absolute number of human $CD3^+$ T cells, $CD3^+CD4^+$ helper/inducer T cells, $CD3^+CD8^+$ cytotoxic T cells, $CD3^-CD19^+$ B cells, and $CD3^-CD16^+$ and/or $CD56^+$ NK cells in peripheral blood was analyzed in WPOI 1-3 (n=119) and 4-5 (n=136) groups using the BD Multitest™ reagent. *P*=multiple t test. (g-k) Graphical representation of the expressions of five classical inflammation mediators (IL-1β, IL-6, IL-8, SDF-1, TNF-α) were simultaneously detected *in situ* in OSCC tissues (n=14). TC: Tumor cell; FC: Fibroblasts; TIL: Tumor-infiltrated lymphocytes. *P*=multiple t test, red box indicated $P<0.05$. (l, m) Graphical quantitation of IHC of fibronectin/PDGFRβ^+ stromal fibroblasts staining from OSCC patient's samples with WPOI 1-3 (n=10) and WPOI 4-5 (n=10) were showed by IHC and IF analysis. *P*=Two-tailed t test. (n) Representative H&E photomicrographs of the micrometastatic foci in tumor invasion front in xenograft mouse model was showed by H&E analysis, n=6. (o) Representative photomicrographs showing the histological shape of tumor cells at the invasion front revealed by pan-CK staining. Collagen components within tumor stroma are indicated by Masson Trichrome staining and Sirius Red staining, n=6. (p) The LNM (Red arrow indicated LNM foci) was showed. Scale bars, 100 μm (n), 100 μm (o). Results are shown as mean and standard deviation (SD). Boxes indicate the first and third quartiles, bands indicate medians, and whiskers indicate ±1.5 interquartile range. Source data are provided as a Source data file.

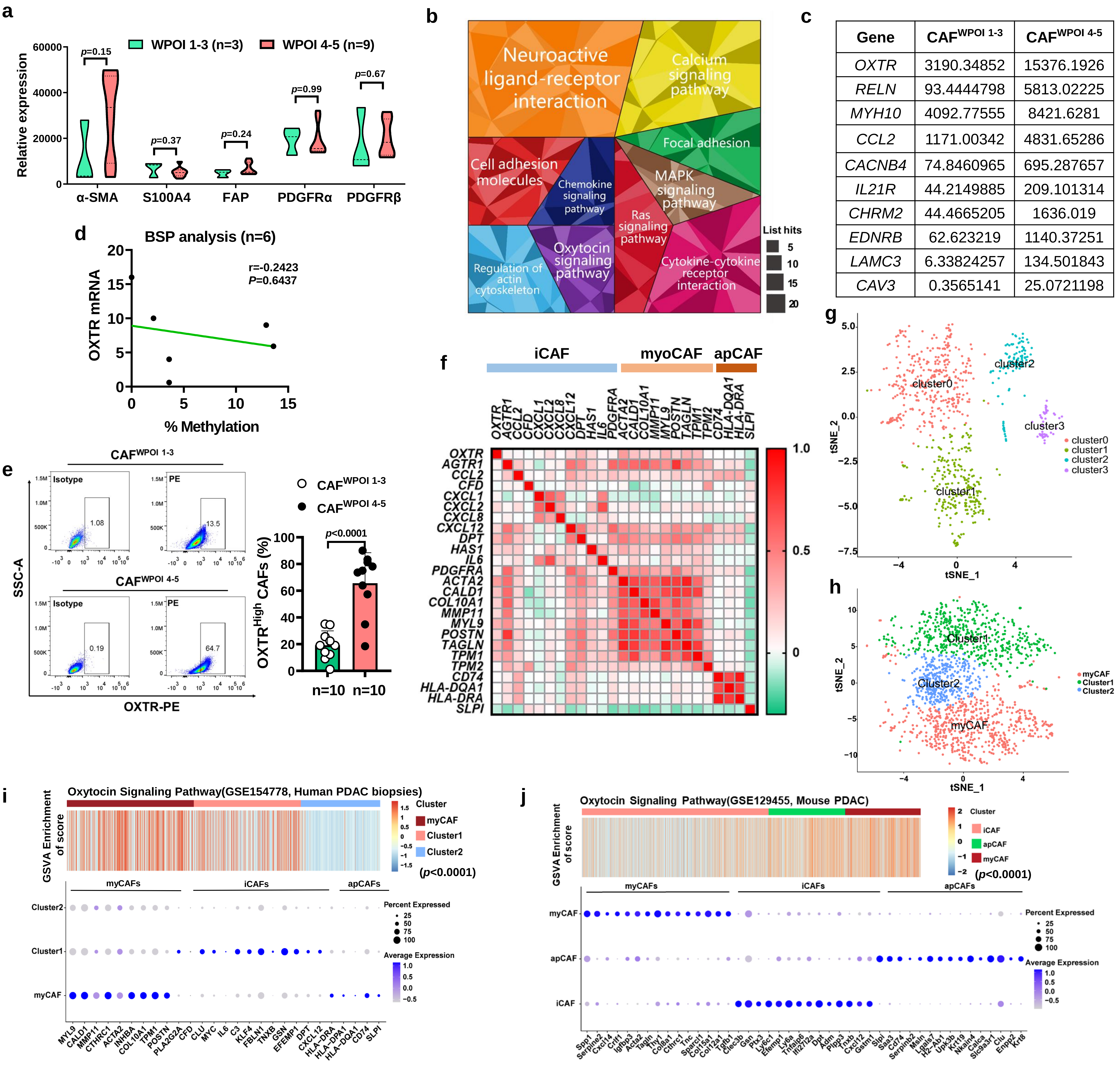

Gene	CAF^WPOI 1-3	CAF^WPOI 4-5
OXTR	3190.34852	15376.1926
RELN	93.4444798	5813.02225
MYH10	4092.77555	8421.6281
CCL2	1171.00342	4831.65286
CACNB4	74.8460965	695.287657
IL21R	44.2149885	209.101314
CHRM2	44.4665205	1636.019
EDNRB	62.623219	1140.37251
LAMC3	6.33824257	134.501843
CAV3	0.3565141	25.0721198

Supplementary Figure 2. OXTR is up-regulated in CAF$^{WPOI\ 4\text{-}5}$. (a) Graphical representation of the relative expression of classical CAF markers in WPOI 1-3 (n=3) and 4-5 (n=9) -derived CAFs. *P*=multiple t test. (b) Representation of KEGG pathway analysis from RNA sequencing and differential gene analysis of CAF$^{WPOI\ 1\text{-}3}$ and CAF$^{WPOI\ 4\text{-}5}$ types. Differential KEGG pathways are shown with the number of gene hits adjusted according to rectangle size. (c) The relative expression of up-regulated gene in the top hit pathways. (d) The methylation of *OXTR* was determined by bisulfite sequencing PCR (BSP) by spearman rank correlation test. (e) Representative flow cytometry analysis showing the proportion of OXTR^{High} CAFs in isolated CAF$^{WPOI\ 1\text{-}3}$ and CAF$^{WPOI\ 4\text{-}5}$ of OSCC, n=10/group. *P*=Two-tailed t test. (f) Heatmap showing OXTR gene correlation in the three CAF subtypes' from HNSCC (n=522, TCGA data set, Firehose Legacy). (g-j) UMAP visualization of CAF cell populations from scRNA-seq of human HNSCC (GSE103322) (g), human PDAC (GSE154778) (h). GSVA of the oxytocin signaling pathway performed on scRNA-seq data sets from human PDAC (GSE154778) (i) and murine PDAC (GSE129455) (j). Similarities between different fibroblasts subtypes and myCAFs, iCAFs and apCAFs were analyzed in the lower bubble plot (p<0.001). *P* = Two-tailed t test. Results are shown as mean and standard deviation (SD). Source data are provided as a Source data file.

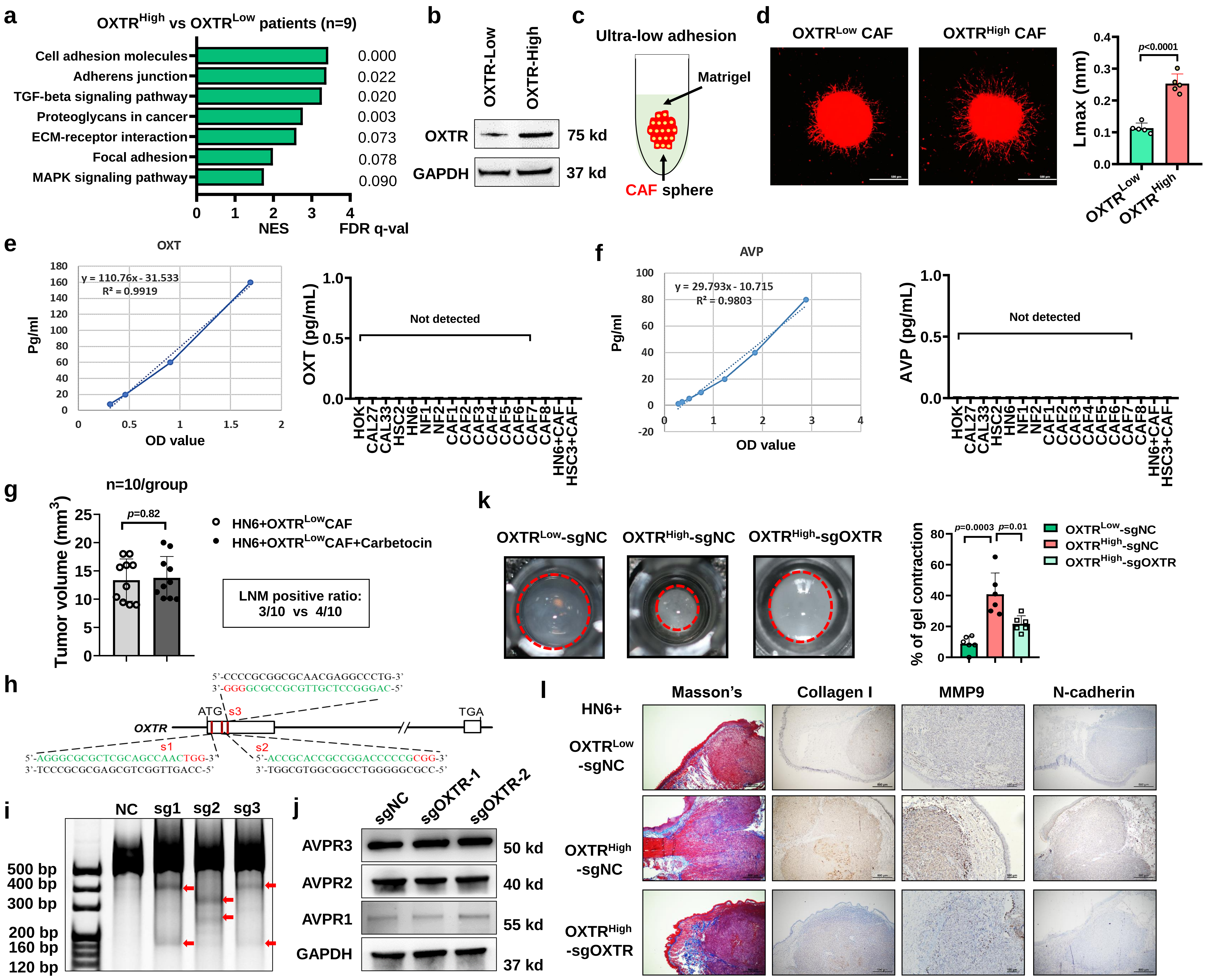

Supplementary Figure 3. OXTR⁺ CAF promotes tumor invasion. (a) Top ECM remodeling pathways and CAF activation signaling from KEGG analysis of $OXTR^{High}$ versus $OXTR^{Low}$ OSCC patients, n=9. (b) IB analysis of OXTR expression in $OXTR^{High}$ and $OXTR^{Low}$ CAFs. The immunoblotting results are representative of three independent experiments. (c, d) Setup of homospheroid formation from 8 × 10^4 DiI (Red)-labeled $OXTR^{low}$ or $OXTR^{high}$ CAFs cultured in Matrigel gels mixed with collagen I, n=5/group. The matrix invasion of CAFs homospheroids was determined by estimating the maximal distance of invasion from the spheroid border (Lmax). *P*=Two-tailed t test. (e, f) Graphical representation of data from ELISA assay to determine OXT and AVP levels from the culture medium of the cells indicated. n=3 biologically independent samples. (g) Tumor volume data and numbers of LNM from and orthotopic model of OSCC in NCG mice as indicated. Carbetocin was used twice weekly (6 mg/kg/mice, i.p.), n=10/group. *P*=Two-tailed t test. (h) Design of three sgRNA-OXTR using a survey of validation strategies for CRISPR-Cas9 editing (T7E1), with (i) their validation from three independent replicates. (j) WB analysis for AVPR1/2/3 levels after the lentiviral plasmid sgRNA-OXTR was transfected into CAFs. The immunoblotting results are representative of three independent experiments. (k) Representative photomicrographs and graphical representation of gel contraction after OXTR knockdown by lentivirus-sgRNA in $OXTR^{High}$ CAFs, n=6/group. *P*=Two-tailed t test. (l) Representative photomicrographs depicting the expression of the collagen components, MMP9 and N-cadherin by IHC analysis in tumor from the indicated samples with estimation by Masson Trichrome staining, n=6. Scale bars, 500 µm (Masson Trichrome staining and Collagen I), 100 µm (MMP9 and N-cadherin). *P* = Student's t test. Results are shown as mean and standard deviation (SD). Source data are provided as a Source data file.

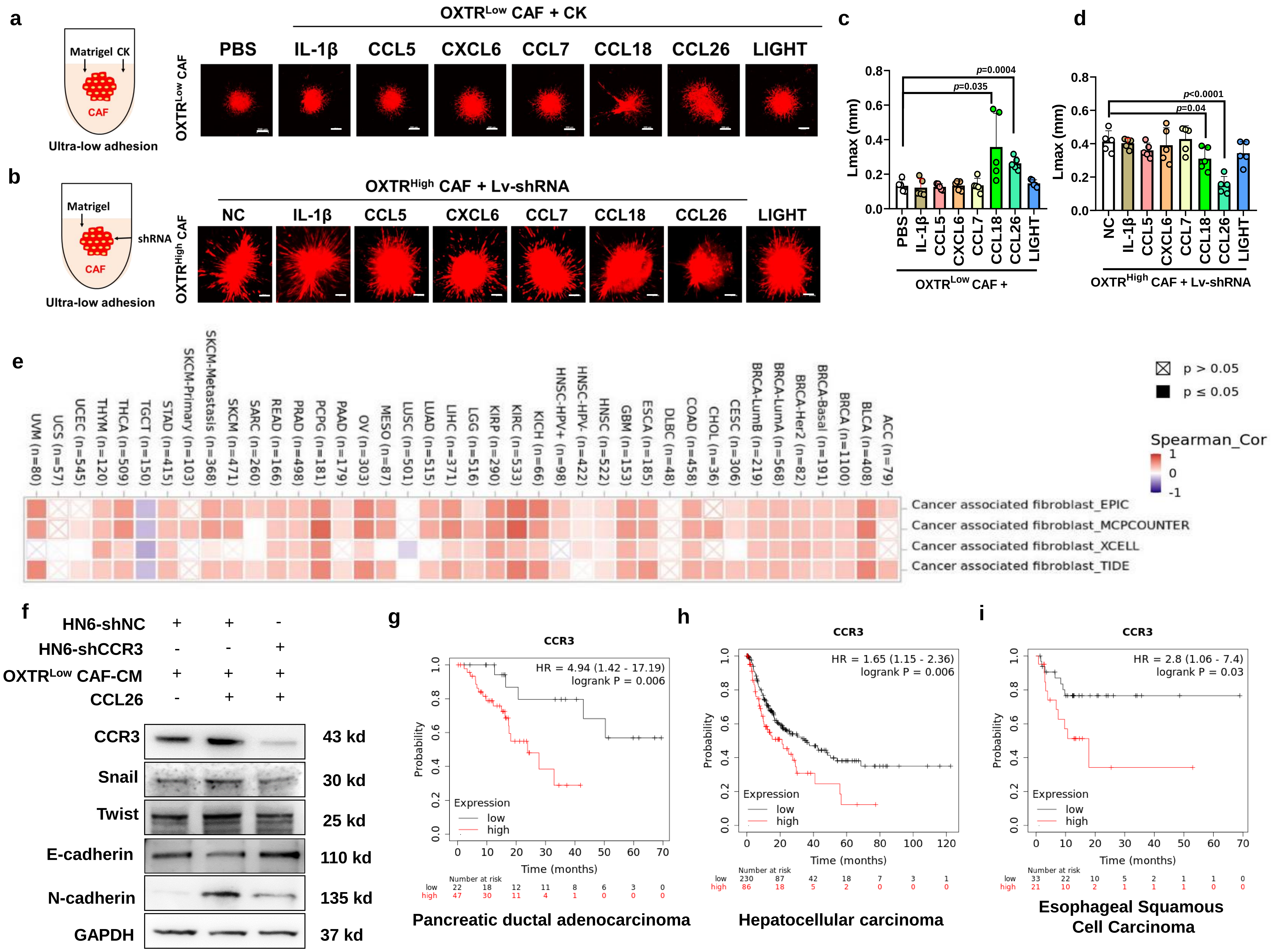

Supplementary Figure 4. CCL26 is require for the pro-invasion function of OXTR⁺CAF**.** (a-d) Set up to establish Homogeneous spheroids, which were embedded into conditional 3D collagen matrix for organoid invasion measurement (8 x 10^4 DiI-labeled conditional CAFs (red). Graphical data of invasion scores are expressed as maximal distance of invasion from the spheroid border (Lmax) of the indicated CAFs using ImageJ software, Scale bar: 200 μm. n=10/group in c and d. *P*=Two-tailed t test. (e) Heat map showing the correlation between CCL26 expression and CAFs markers from multiple cancers (http://timer.cistrome.org/). (f) EMT signature determined by WB analysis for the indicated proteins from HN6 with CCR3 knockdown treated with OXTR^{Low} CAFs-CM and/or CCL26. This experiment were repeated twice and the results were reproducible. (g-i) Graphical representation of the correlation between CCR3 expression and recurrence-free survival in PDAC, hepatocellular carcinoma and esophageal squamous cell carcinoma by Kaplan-Meier plotter (http://kmplot.com/analysis/). *P* = Two-tailed t test. Results are shown as mean and standard deviation (SD). Source data are provided as a Source data file.

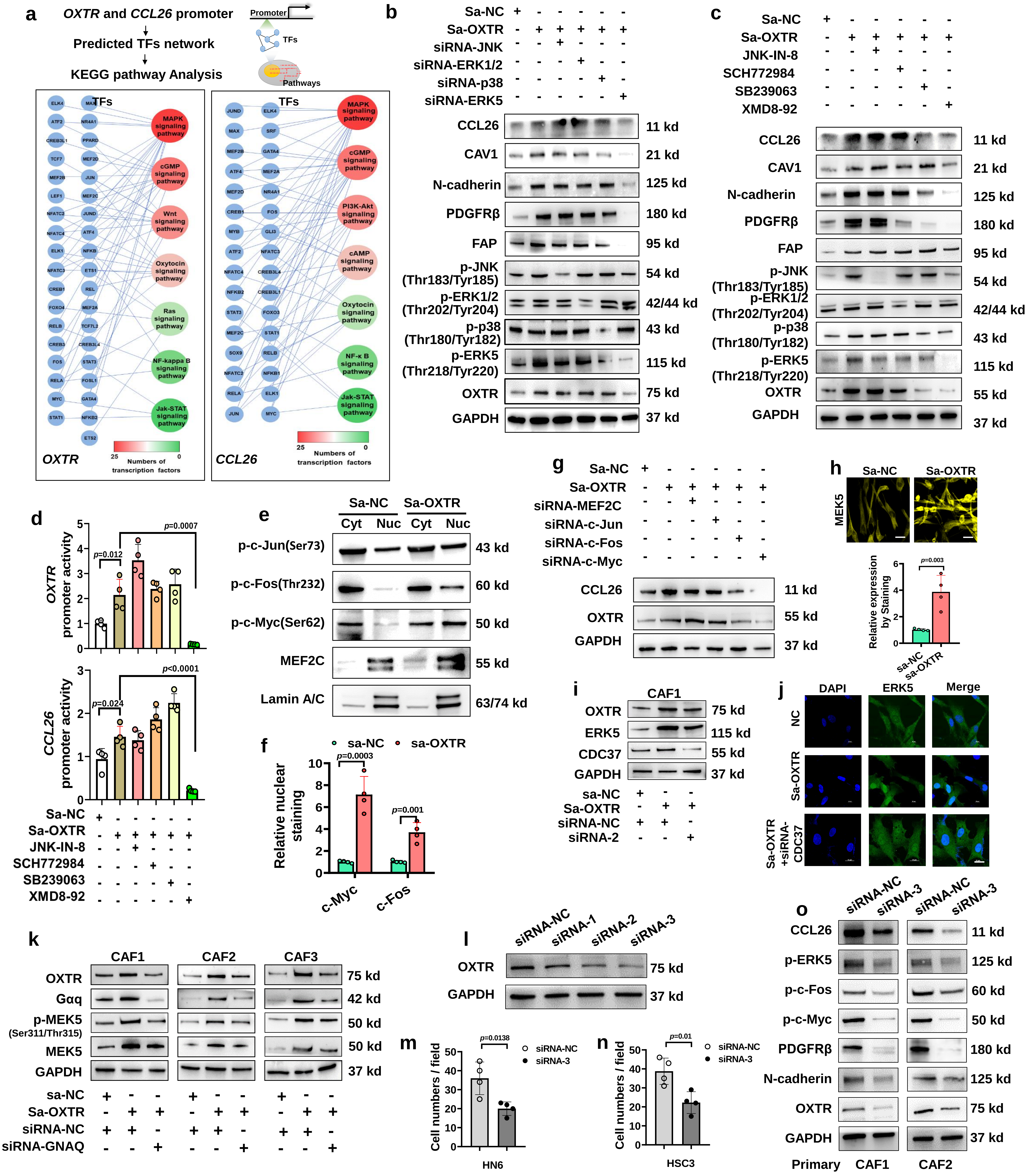

a
OXTR and CCL26 promoter
Predicted TFs network
KEGG pathway Analysis
Promoter
TFs
Pathways
OXTR
CCL26
MAPK signaling pathway
cGMP signaling pathway
Wnt signaling pathway
Oxytocin signaling pathway
Ras signaling pathway
NF-kappa B signaling pathway
Jak-STAT signaling pathway
PI3K-Akt signaling pathway
cAMP signaling pathway
NF-κ B signaling pathway
Numbers of transcription factors
b
Sa-NC
Sa-OXTR
siRNA-JNK
siRNA-ERK1/2
siRNA-p38
siRNA-ERK5
CCL26 11 kd
CAV1 21 kd
N-cadherin 125 kd
PDGFRβ 180 kd
FAP 95 kd
p-JNK (Thr183/Tyr185) 54 kd
p-ERK1/2 (Thr202/Tyr204) 42/44 kd
p-p38 (Thr180/Tyr182) 43 kd
p-ERK5 (Thr218/Tyr220) 115 kd
OXTR 75 kd
GAPDH 37 kd
c
JNK-IN-8
SCH772984
SB239063
XMD8-92
OXTR 55 kd
d
OXTR promoter activity
CCL26 promoter activity
p=0.012
p=0.0007
p=0.024
p<0.0001
e
Cyt
Nuc
p-c-Jun(Ser73) 43 kd
p-c-Fos(Thr232) 60 kd
p-c-Myc(Ser62) 50 kd
MEF2C 55 kd
Lamin A/C 63/74 kd
f
sa-NC
sa-OXTR
Relative nuclear staining
p=0.0003
p=0.001
c-Myc
c-Fos
g
siRNA-MEF2C
siRNA-c-Jun
siRNA-c-Fos
siRNA-c-Myc
h
MEK5
Relative expression by Staining
p=0.003
i
CAF1
ERK5 115 kd
CDC37 55 kd
siRNA-NC
siRNA-2
j
DAPI
ERK5
Merge
NC
Sa-OXTR
Sa-OXTR +siRNA-CDC37
k
CAF2
CAF3
Gαq 42 kd
p-MEK5 (Ser311/Thr315) 50 kd
MEK5 50 kd
siRNA-GNAQ
l
siRNA-1
siRNA-3
m
Cell numbers / field
p=0.0138
HN6
n
p=0.01
HSC3
o
p-ERK5 125 kd
p-c-Fos 60 kd
p-c-Myc 50 kd
Primary

Supplementary Figure 5. ERK5 activation by Gq and CDC37 is critical for OXTR and CCL26 expression**.** (a) GTRD analysis for predicted transcription factors (TFs) in the promoter regions of CCL26 and OXTR showing enriched KEGG pathways. (b, c) WB analysis of CAFs for the proteins indicated after treatment with sa-NC or sa-OXTR and specific knockdown or inhibitors of JNK (JNK-IN-8), ERK1/2 (SCH772984), p38 (SB239063) or ERK5 (XMD8-92). (d) Graphical analysis of the promoter activity effects of specific inhibitors of JNK (JNK-IN-8), ERK1/2 (SCH772984), p38 (SB239063) and ERK5 (XMD8-92) on OXTR and CCL26 transcription using a dual-luciferase reporter assay system. n=4/group *P*=Two-tailed t test. (e) WB analysis for nuclear c-Fos and c-Myc of CAFs transfected with sa-NC and sa-OXTR and (f) accompanying IF staining (n=4/group). *P*=Two-tailed t test. (g) Representative western blots showing knockdown of four TFs (c-fos, c-jun, c-myc and MEF2C) in CAFs and the effect on OXTR and CCL26 protein expression. (h) Representative IF images from CAFs transfected with sa-NC and sa-OXTR, showing MEK5 localization, n=4/group. *P*=Two-tailed t test. Scale bar: 50 μm (i) WB for OXTR/ERK5 expression/activation from sa-OXTR CAFs with knockdown of CDC37 by siRNA, with ERK5 localization by IF (j). Scale bar: 10 μm. (k) WB for OXTR/MEK5 expression or activation from CAFs 1-3 treated with siRNA-GNAQ. (l-n) WB for OXTR in primary CAFs inhibited by siRNA and graphical representation of invasive cell number when co-cultured with OSCC cells in the Transwell system, n=4/group. *P*=Two-tailed t test. (o) WB for the indicated proteins involved in OXTR/ERK5/TFs signaling in two primary CAFs with siRNA-OXTR. Results are shown as mean and standard deviation (SD). All immunoblotting results are representative of three independent experiments. Source data are provided as a Source data file.

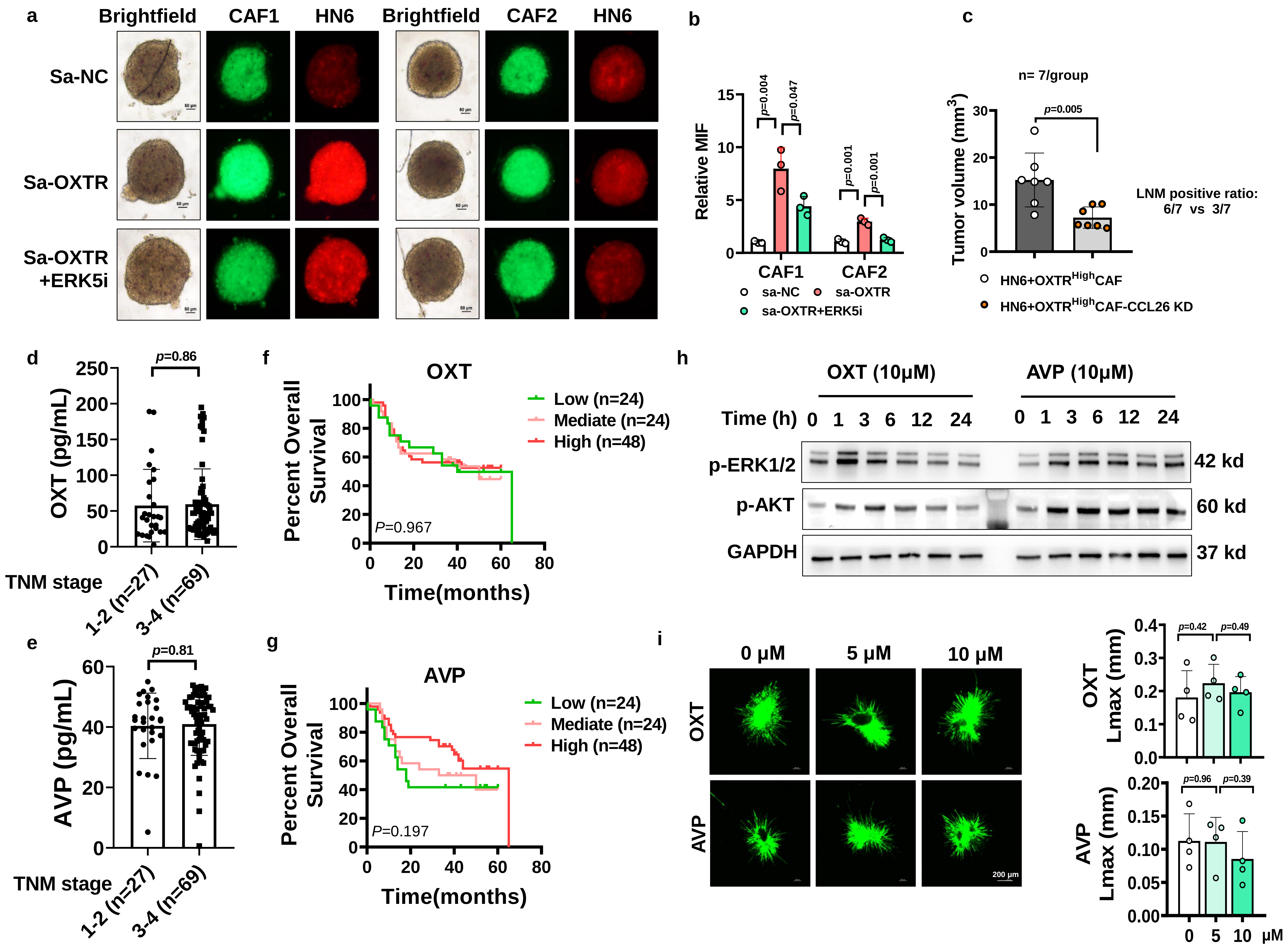

Supplementary Figure 6. OXT showed no impacts on WPOI. (a, b) Representative images of co-culture of HN6 with CAFs (6 x10^4) in an ultra-low adhesive environment, showing formation of heterotypic spheroids after 10 days, with (b) Mean fluorescence intensity (MIF), n=3/group. *P*=Two-tailed t test. (c) Graphical analysis of tongue tumor volume or LMN percentage after 2×10^5 HN6 OSCC cells were mixed with 1×10^6 sa-OXTR conditional CAFs (with/without CCL26 knockdown) and injected into the tongue of 4- to 5-week-old NCG mice. *P*=Two-tailed t test. (d, e) Graphical representation of the levels (n=27 in TNM 1-2 group and n=69 in TNM 3-4 group). *P*=Two-tailed t test. (f, g) prognostic value of serum OXT and AVP in patients (n=96) analyzed by ELISA and Kaplan-Meier curves (logrank tests). (h) WB for activated ERK1/2 and AKT protein in CAFs after treatment OXT and AVP for indicated times. This experiment were repeated twice and the results were reproducible. (i) Representative IF images and graphical analysis of Lmax (invasion in 3D collagen matrix) of fibroblasts treated with OXT or AVP. n=4/group, *P*=Two-tailed t test. Results are shown as mean and standard deviation (SD). Source data are provided as a Source data file.

Supplementary Table 1 Detailed information on the antibodies, reagents, cell lines, sgRNA and saRNA etc.

REAGENT or RESOURCE	SOURCE	IDENTIFIER
**Antibodies and dilutions**		
Anti-alpha smooth muscle Actin[1A4], IHC-P (0.034 µg/ml), WB (0.341 µg/ml), IF (1:100)	abcam	ab7817
Anti-wide spectrum Cytokeratin, IHC-P (1:100), WB (1:1000)	abcam	ab9377
Donkey anti-Rabbit IgG (H+L) Highly Cross-Adsorbed Secondary Antibody, Alexa Fluor 488, IF (1:300)	life	A-21206
Donkey anti-Mouse IgG (H+L) Highly Cross-Adsorbed Secondary Antibody, Alexa Fluor Plus 594, IF (1:300)	life	A32744
Anti-Oxytocin Receptor, WB (1:500)	abcam	ab87312
Oxytocin Receptor Antibody, FC (0.2 ug/sample)	proteintech	23045-1-AP
Anti-Caveolin-1[7C8], WB (1:50)	abcam	ab17052
OXTR Polyclonal Antibody (Lot:B7201, B72RC9), WB (1:1000), IHC (1:10000)	ImmunoWay	YN2672
Rabbit Anti-OXTR, NT (OXTR, Oxytocin receptor) (APC), FC (2ul/sample)	Usbio	039556-APC
Anti-Oxytocin Receptor[EPR12789], IF (1:200)	abcam	ab181077
Anti-Caveolin-1[E249] - Caveolae Marker, WB (1:1000)	abcam	ab32577
Phospho-p44/42 MAPK (Erk1/2) (Thr202/Tyr204) (D13.14.4E) XP Rabbit mAb, IF (1:200), WB (1:1000)	CST	4370T
Alexa Fluor 647-AffiniPure Goat Anti-Rabbit IgG (H+L), IF (1:500)	Jackson	111-605-003
Anti-GNAQ, WB (1 µg/ml), IP (1:50)	abcam	ab75825
Anti-CD146[EPR3208], IHC (1:250)	abcam	ab75769
Anti-FOXP3[EPR22102-37], IHC (1:250)	abcam	ab215206
Anti-CCR3[Y31], IHC (1:100), WB (1:500)	abcam	ab32512
Phospho-c-Jun (Ser73) (D47G9) XP Rabbit mAb, IF (1:500), WB (1:1000)	CST	3270T
Erk5 (D3I5V) Rabbit mAb, IP (1:50), WB (1:1000)	CST	12950S
Phospho-Erk5 (Thr218/Tyr220) Antibody, WB (1:1000)	CST	3371S
Phospho-c-Fos (Ser32) (D82C12) XP Rabbit mAb, IF (1:200), WB (1:1000)	CST	5348T
c-Myc Antibody, WB (1:1000)	CST	9402S
PDGF Receptor β (28E1) Rabbit mAb, IF (1:100), WB (1:1000)	CST	3169S
Anti-MEF2C[EPR19089-202] - ChIP Grade, WB (1:1000)	abcam	ab211493
CD29-PE, human 30 tests, FC (1ul/sample)	miltenyi	130-101-275
Lamin A/C (4C11) Mouse mAb, WB (1:1000)	CST	#4777T

Phospho-c-Fos (Ser32) (D82C12) XP Rabbit mAb, IF (1:200), WB (1:1000)	CST	5348S
ERK 5 antibody (C-7), IF (1:50)	Santacruz	sc-398015
β-Arrestin 1/2 (D24H9) Rabbit mAb, WB (1:1000), IP (1:100)	CST	4674s
Anti-Beta Arrestin 1 and 2, WB (1:1000)	abcam	ab32099/54790
SUMO-2/3 (18H8) Rabbit mAb, WB (1:1000)	CST	4971T
HSP90 (C45G5) Rabbit mAb, WB (1:1000)	CST	4877T
CDC37 (D11A3) XP Rabbit mAb, WB (1:1000)	CST	4793S
c-Fos (phospho Ser374) Polyclonal Antibody, WB (1:1000)	ImmunoWay	YP1095
Anti-MEK5 (phospho S311 + T315), WB (1:1000)	abcam	ab254134
Anti-MEK5, WB (1:1000)	abcam	ab210748
Human FAP Phycoerythrin MAb (Clone 427819) (25 TESTS), FC (2ul/sample)	R&D	FAB3715P-025
AVP Receptor V3 Polyclonal Antibody, WB (1:1000)	ImmunoWay	YT6001
AVP Receptor V2 Polyclonal Antibody, WB (1:1000)	ImmunoWay	YT0423
AVPR1A Polyclonal Antibody, WB (1:1000)	ImmunoWay	YT6097
PKC ζ Polyclonal Antibody, WB (1:1000)	ImmunoWay	YT3765
PKC ζ (phospho Thr560) Polyclonal Antibody, WB (1:1000)	ImmunoWay	YP0230
Epithelial-Mesenchymal Transition (EMT) IF Antibody Sampler Kit, WB (1:500-1000)	Cell Signaling	49398
ECM Profiling Sampler Kit, IF (1:100-400)	Cell Signaling	33437
APC anti-human CD31, FC (2ul/sample)	BioLegend	303115
APC anti-human CD326 (EpCAM), FC (1.5ul/sample)	BioLegend	369809
APC anti-human CD45, FC (2ul/sample)	BioLegend	368511
CD4, IHC (ready-to-use)	Zhong Shan -Golden Bridge	ZM-0418
CD8, IHC (ready-to-use)	Zhong Shan -Golden Bridge	ZA-0508
CD68, IHC (ready-to-use)	Zhong Shan -Golden Bridge	ZM-0464
Collagen Ⅰ, IHC (ready-to-use)	Zhong Shan -Golden Bridge	ZA-0616
MMP-9, IHC (ready-to-use)	Zhong Shan -Golden Bridge	ZA-0562
Anti-TSC-1 (CCL-26), WB (0.5 μg/ml)	abcam	ab217328
**Bacterial and virus strains**		
CMV-hTERT (Puro) Lentifect™ Purified Lentiviral Particles	GeneCopoeia	LPP-Q0450-Lv105-100-C
**Biological samples**		
Human OSCC tissue and serum samples	Nanjing Stomatological Hospital	N/A
**Chemicals, peptides, and recombinant proteins**		
Fibroblast Medium	Sciencell	2301
CELLTRACKER GREEN CMF 20 X 50 UG	life	C7025

CellTracker CM-DiI Dye	life	C7000
Protein A/G Magnetic Beads	MCE	HY-K0202
Erlotinib HCl (OSI-744)	selleckchem	S1023
SB239063	selleckchem	S7741
XMD8-92	selleckchem	S7525
U73122	selleckchem	S8011
MK-8353 (SCH900353)	selleck	S8701
Ravoxertinib (GDC-0994)	selleck	S7554
SCH772984	selleck	S7101
Human recombinant Eotaxin-3/TSC (CCL26)	Peprotech	300-48-20
Human recombinant MCP-3 (CCL7)	Peprotech	300-17-10
Human recombinant IL-1beta	Peprotech	200-01B-10
Human recombinant LIGHT (insect cells)	Peprotech	310-09B-15
Human recombinant GCP-2 (CXCL6)	Peprotech	300-41-20
Human recombinant RANTES (CCL5)	Peprotech	300-06-20
Human recombinant MIP-4/PARC (CCL18)	Peprotech	300-34-10
JNK-IN-8	selleckchem	S4901
SCH772984	selleckchem	S7101
SB239063	selleckchem	S7741
XMD8-92	selleckchem	S7525
**Critical commercial assays**		
3D invasion via PrimeSurface 96U plate	Primesurface	MS-9096UZ
3D invasion via PrimeSurface 96V plate	Primesurface	MS-9096VZ
Millicell® Hanging 24-well Cell Culture Insert,8.0 μm	Merck&Millipore	MCEP24H48
NE-PER Nuclear and Cytoplasmic Extraction Reagents	Thermo	78833
APC-Antibody conjugation Kit / APC	Fcmacs	FMS-ABAPC0002
R-PE-Antibody conjugation Kit / R-PE	Fcmacs	FMS-ABPE0001
Alexa Fluor 488?-Antibody?conjμgation?Kit	Fcmacs	FMS-ABAF4880004
Dual-Luciferase Reporter Assay System	Promega	E1910
DynaMag™-2Magnet	life	12321D
Human oxytocin,OT ELISA Kit	cusabio	CSB-E08994h
Human antidiuretic hormone/vasopressin/arginine vasopressin,ADH/VP/AVP ELISA Kit	cusabio	CSB-E09080h
MATRIGEL MATRIX GFR NO PHENOL RD	Corning BioCoat	356231
COLLAGEN I RAT TAIL HI PROTEIN	Corning BioCoat	354249
Tumor Dissociation Kit	miltenyi	130-096-730
**Deposited data**		
RNA-suq of WPOI 1-3 and WPOI 4-5-derived CAFs	This paper	PRJNA741552
RNA-suq of OXTR^{low} and OXTR^{high} CAFs	This paper	PRJNA741553
ATAC-seq of saRNA-NC and saRNA-OXTR-expressed CAFs	This paper	PRJNA741554

**Tumor Cell lines**		
Human HSC3	Michigan University	N/A
Human HN6	Shanghai Jiao Tong University	N/A
Mice SCC-7	Shanghai Jiao Tong University	N/A
**Experimental models: Organisms/strains**		
NCG	GemPharmatech	T001475
B6/JGpt-*Oxtr*^{em1Cflox}/Gpt	GemPharmatech	T005928
B6/JGpt-Tg(S100a4-CreERT2-PolyA)3/Gpt	GemPharmatech	T006589
**Oligonucleotides**		
OXTR sgRNA-1: 5'- CAGGGCCTCGTTGCGCCGCG-3'	OBiO Technology	Y11044
OXTR sgRNA-2: 5'- AGGGCGCGCTCGCAGCCAAC-3'	OBiO Technology	Y11045
OXTR sgRNA-3: 5'- ACCGCACCGCCGGACCCCCG-3'	OBiO Technology	Y11046
shRNA targeting human CXCL6, CCL26, CCL18, IL-1β, CCL7, CCL5 and TNFSF14/LIGHT gene (see below)	OBiO Technology	Y12003-Y12009
siRNA targeting human JNK, ERK1/2, p38, ERK5 gene	OBiO Technology	RX016255
OXTR saRNA-1: 5'-CTGGGTTTCCTTAATCCACAA-3'	Genechem	GIDE0252476
OXTR saRNA-2: 5'-AAGCACAACACTGTCTTCATT-3'	Genechem	GIDE0252477
OXTR saRNA-3: 5'-CGGGTAGATAGTGATGAAGTT-3'	Genechem	GIDE0252478
OXTR saRNA-4: 5'-TGGCAGGCAGTATTAATCAAT-3'	Genechem	GIDE0252479
**Recombinant DNA**		
pSLenti-U6-shRNA-CMV-EGFP-F2A-Puro-WPRE	OBiO Technology	N/A
pLenti-U6-sgRNA v2.0-CMV-sfGFP-P2A-3Flag-spCas9	OBiO Technology	N/A
pGL4.10	OBiO Technology	N/A
**Software and algorithms**		
Graphpad Prism 8	Graphpad	https://www.graphpad.com/scientificsoftware/prism/
ImageJ	NIH	https://imagej.nih.gov/ij/
FlowJo 10.0	Flowjo	https://www.flowjo.com/
SPSS	IBM	https://www.ibm.com/cn-zh/products/spss-statistics

Supplementary Table 2. The primers of q-pcr.

Primers for q-PCR		
**Gene**	**Primer**	**Species**
**OXTR**	F: TACCTGCTGCTGCTCATGT	Human
	R: TCGCGCAGAGAGAAGATGT	
**GAPDH**	F:GGAGCGAGATCCCTCCAAAAT	Human
	R:GGCTGTTGTCATACTTCTCATGG	
**S100A4**	F:GATGAGCAACTTGGACAGCAA	Human
	R:CTGGGCTGCTTATCTGGGAAG	
**PDGFRB**	F:AGCACCTTCGTTCTGACCTG	Human
	R:TATTCTCCCGTGTCTAGCCCA	
**CDH2**	F:TCAGGCGTCTGTAGAGGCTT	Human
	R:ATGCACATCCTTCGATAAGACTG	
**FAP**	F:ATGAGCTTCCTCGTCCAATTCA	Human
	R:AGACCACCAGAGAGCATATTTTG	
**ACTA2**	F:AAAAGACAGCTACGTGGGTGA	Human
	R:GCCATGTTCTATCGGGTACTTC	
**VIM**	F:GACGCCATCAACACCGAGTT	Human
	R:CTTTGTCGTTGGTTAGCTGGT	
**CCL5**	F:CCAGCAGTCGTCTTTGTCAC	Human
	R:CTCTGGGTTGGCACACACTT	
**IL-1β**	F:ATGATGGCTTATTACAGTGGCAA	Human
	R:GTCGGAGATTCGTAGCTGGA	
**CCL7**	F:TGCTCAGCCAGTTGGGATTA	Human
	R:TGGCTACTGGTGGTCCTTCT	
**CXCL6**	F:AGAGCTGCGTTGCACTTGTT	Human
	R:GCAGTTTACCAATCGTTTTGGGG	
**CCL18**	F:AAGCCAGGTGTCATCCTCCT	Human
	R:GGGCATAGCAGATGGGACT	
**CCL26**	F:GGGAGATCCACACAGAGGAA	Human
	R:TGTGGCTGTATTGGAAGCAG	
**TNFSF14**	F:ATACAAGAGCGAAGGTCTCACG	Human
	R:CTGAGTCTCCCATAACAGCGG	
**CAV-1**	F:GCGACCCTAAACACCTCAAC	Human
	R:ATGCCGTCAAAACTGTGTGTC	
**CD29**	F:CCTACTTCTGCACGATGTGATG	Human
	R:CCTTTGCTACGGTTGGTTACATT	
**MAPK7**	F:TTTGCCTTACTTCCCACCTG	Human
	R:CCCATGTCGAAAGACTGGTT	
**MAPK8**	F:TGTGTGGAATCAAGCACCTTC	Human
	R:AGGCGTCATCATAAAACTCGTTC	
**MAPK1**	F:TACACCAACCTCTCGTACATCG	Human
	R:CATGTCTGAAGCGCAGTAAGATT	
**MAPK14**	F:TCAGTCCATCATTCATGCGAAA	Human
	R:AACGTCCAACAGACCAATCAC	
**JUN**	F:TCCAAGTGCCGAAAAAGGAAG	Human
	R:CGAGTTCTGAGCTTTCAAGGT	
**FOS**	F:CCGGGGATAGCCTCTCTTACT	Human
	R:CCAGGTCCGTGCAGAAGTC	
**MYC**	F:GGCTCCTGGCAAAAGGTCA	Human
	R:CTGCGTAGTTGTGCTGATGT	
**MEF2C**	R:GAACGTAACAGACAGGTGACAT	Human
	F:CGGCTCGTTGTACTCCGTG	

Supplementary Table 3. The siRNA sequence used in this study.

siRNA/shRNA sequence		
**Gene**	**Primer (5'-3')**	**Species**
**CCL5**	CGAAAGAACCGCCAAGTGT	Human
**IL-1β**	GCGUGUUGAAAGAUGAUAATT	Human
**CCL7**	UCAAGACCAAACUGGACAATT	Human
**CXCL6**	GCTGCGTTGCACTTGTTTA	Human
**CCL18**	CCUGGCAGAUUCCACAAAATT	Human
**CCL26**	CCAAAAGAGGCAAGAAAGUTT	Human
**TNFSF14**	GGCCCUCAGUGUUUGUGGUTT	Human
**MAPK7**	CCGAGCUCAUGCUCUCUUUTT	Human
**MAPK8**	CCUUCAUUCUGCUGGAAUUTT	Human
**MAPK1**	CCCACACAAGAGGAUUGAATT	Human
**MAPK14**	GGCACACAGAUGAUGAAAUTT	Human
**JUN**	AAGTCATGAACCACGTTAA	Human
**CCR3**	GCTCCGAATTATGACCAACAT	Human
**FOS**	GCAAGGTGGAACAGTTATC	Human
**MYC**	GGGTCAAGTTGGACAGTGT	Human
**MEF2C**	CGTAGCAACTCCTACTTTA	Human
**GNAQ**	CGGTTATTCTGTTCTTAAA	Human
**CDC37**	CGATGAGAAGGATGTCAGT	Human
