# Supplementary material for: OXTRHigh stroma fibroblasts control the invasion pattern of oral squamous cell carcinoma via ERK5 signaling
Source: Nat Commun. 2022 Aug 31;13:5124. doi: 10.1038/s41467-022-32787-y (PMC9433374; doi:10.1038/s41467-022-32787-y)
Supplement: Supplementary file 3 — Reporting Summary [file 41467_2022_32787_MOESM3_ESM.pdf]

nature portfolio

Corresponding author(s): Dr. Yanhong Ni

Last updated by author(s): Aug 16, 2022

Reporting Summary

Nature Portfolio wishes to improve the reproducibility of the work that we publish. This form provides structure for consistency and transparency in reporting. For further information on Nature Portfolio policies, see our Editorial Policies and the Editorial Policy Checklist.

Statistics

For all statistical analyses, confirm that the following items are present in the figure legend, table legend, main text, or Methods section.

n/a	Confirmed	
☐	☒	The exact sample size (*n*) for each experimental group/condition, given as a discrete number and unit of measurement
☐	☒	A statement on whether measurements were taken from distinct samples or whether the same sample was measured repeatedly
☐	☒	The statistical test(s) used AND whether they are one- or two-sided<br>*Only common tests should be described solely by name; describe more complex techniques in the Methods section.*
☒	☐	A description of all covariates tested
☒	☐	A description of any assumptions or corrections, such as tests of normality and adjustment for multiple comparisons
☐	☒	A full description of the statistical parameters including central tendency (e.g. means) or other basic estimates (e.g. regression coefficient) AND variation (e.g. standard deviation) or associated estimates of uncertainty (e.g. confidence intervals)
☐	☒	For null hypothesis testing, the test statistic (e.g. *F*, *t*, *r*) with confidence intervals, effect sizes, degrees of freedom and *P* value noted<br>*Give P values as exact values whenever suitable.*
☒	☐	For Bayesian analysis, information on the choice of priors and Markov chain Monte Carlo settings
☒	☐	For hierarchical and complex designs, identification of the appropriate level for tests and full reporting of outcomes
☐	☒	Estimates of effect sizes (e.g. Cohen's *d*, Pearson's *r*), indicating how they were calculated

Our web collection on statistics for biologists contains articles on many of the points above.

Software and code

Policy information about availability of computer code

Data collection	1. mRNA data was obtained from RNA-Seq Kit v2.0 (Life Technologies)； 2. Fluorescence microscopy images were obtained from Nikon Confocal Microscope Ti; 3. Flow cytometry (sony SH800) was used for analysis of protein expression and cell sorting.
Data analysis	1. h.all.v7.0.symbols (Hallmarks)；2. c5.all.v7.0.symbols (C5)；3.Graphpad Prism 8；4.FlowJo 10.0；5.ImageJ 1.8.0；6. SPSS software program 18.0 (SPSS Inc).

For manuscripts utilizing custom algorithms or software that are central to the research but not yet described in published literature, software must be made available to editors and reviewers. We strongly encourage code deposition in a community repository (e.g. GitHub). See the Nature Portfolio guidelines for submitting code & software for further information.

Data

Policy information about availability of data

All manuscripts must include a data availability statement. This statement should provide the following information, where applicable:

- Accession codes, unique identifiers, or web links for publicly available datasets
- A description of any restrictions on data availability
- For clinical datasets or third party data, please ensure that the statement adheres to our policy

The mRNA expression profile data and ATAC reported generated in this study have been deposited in the Sequence Read Archive (SRA) database with accession number PRJNA741552 (https://www.ncbi.nlm.nih.gov/bioproject/PRJNA741552/), PRJNA741553 (https://www.ncbi.nlm.nih.gov/bioproject/?term=PRJNA741553) and PRJNA741554 (https://www.ncbi.nlm.nih.gov/bioproject/?term=PRJNA741554). The publicly available data in Figure 2c and 4a (TCGA, Firehose Legacy) are available in the cBioPortal database (http://www.cbioportal.org/study/summary?id=hnsc_tcga) and are used to perform GSEA analysis by MSigDB database (http://www.broadinstitute.org/gsea/msigdb/index.jsp). The publicly available data in Figure 5O and supplementary Figure 4g-i are available in the KM-Plotter pan-Cancer

database (https://kmplot.com/analysis/). The publicly available data in supplementary Figure 4e is available in TIMER 2.0 database (http://timer.cistrome.org/). The publicly available data in Figure 2d, 2i, supplementary Figure 2g-j is available in the GEO database with accession number GSE68241 (https://www.ncbi.nlm.nih.gov/geo/query/acc.cgi?acc=GSE68241), GSE81850 (https://www.ncbi.nlm.nih.gov/geo/query/acc.cgi?acc=GSE81850), GSE103322 (https://www.ncbi.nlm.nih.gov/geo/query/acc.cgi?acc=GSE103322), GSE154778 (https://www.ncbi.nlm.nih.gov/geo/query/acc.cgi?acc=GSE154778) and GSE129455 (https://www.ncbi.nlm.nih.gov/geo/query/acc.cgi?acc=GSE129455). The remaining data are available within the Article, Supplementary Information or Source Data file

Field-specific reporting

Please select the one below that is the best fit for your research. If you are not sure, read the appropriate sections before making your selection.

☒ Life sciences ☐ Behavioural & social sciences ☐ Ecological, evolutionary & environmental sciences

For a reference copy of the document with all sections, see nature.com/documents/nr-reporting-summary-flat.pdf

Life sciences study design

All studies must disclose on these points even when the disclosure is negative.

Sample size

No statistical methods were used to pre-determine sample size. Sample size of all experiments are determined based on common practices of the fields.
1. a cohort of 880 OSCC patients with early stage or advanced stage was used to assess the proportions of WPOI subtypes;
2. Immunofluorescence staining of EMT-related markers from OSCC tumor samples of WPOI 1-3 and 4-5 types (n=8/WPOI type);
3. Relapse-free survival analysis on early-stage (n=523) and late stage-stage (n=126) OSCC patients classified as WPOI types 1-3 or 4-5;
4. Stromal components were estimated in 20 WPOI 1-3 and 4-5 type OSCC samples by IHC analysis;
5. Graphical summary from IHC analysis of α-SMA+ stained stromal fibroblasts from tumor centers or at the invasion front of OSCC (patients n=150);
6. RNA sequencing on CAF-WPOI 1-3 and CAF-WPOI 4-5 types (n=12);
7. IHC analysis on OXTR+ CAFs from OSCC patients, n=196.;
8. NCG mice used in each model (n>=6/group);
9. Representative IHC images showing nuclear ERK5 or CCL26 expression in OSCC patient tissue (n=106);
10. Graphical representation of analysis of Serum OXT and AVP levels from patients with WPOI 1-3 and 4-5 types as indicated and as determined by ELISA (n=180+96).

Data exclusions

Patients diagnosed with autoimmune or other malignant diseases as well as pregnant or lactating individuals were excluded from this study. No patient included in this study had undergone preoperative chemotherapy and/or radiotherapy interventions.

Replication

Reproducibility was assessed by replicas and repeated experiments. All experiments were performed at least two times with similar results.

Randomization

All patients in this study were collected randomly.

Blinding

Investigators were blinded to group allocation during data collection

Behavioural & social sciences study design

All studies must disclose on these points even when the disclosure is negative.

Study description

Briefly describe the study type including whether data are quantitative, qualitative, or mixed-methods (e.g. qualitative cross-sectional, quantitative experimental, mixed-methods case study).

Research sample

State the research sample (e.g. Harvard university undergraduates, villagers in rural India) and provide relevant demographic information (e.g. age, sex) and indicate whether the sample is representative. Provide a rationale for the study sample chosen. For studies involving existing datasets, please describe the dataset and source.

Sampling strategy

Describe the sampling procedure (e.g. random, snowball, stratified, convenience). Describe the statistical methods that were used to predetermine sample size OR if no sample-size calculation was performed, describe how sample sizes were chosen and provide a rationale for why these sample sizes are sufficient. For qualitative data, please indicate whether data saturation was considered, and what criteria were used to decide that no further sampling was needed.

Data collection

Provide details about the data collection procedure, including the instruments or devices used to record the data (e.g. pen and paper, computer, eye tracker, video or audio equipment) whether anyone was present besides the participant(s) and the researcher, and whether the researcher was blind to experimental condition and/or the study hypothesis during data collection.

Timing

Indicate the start and stop dates of data collection. If there is a gap between collection periods, state the dates for each sample cohort.

Data exclusions	*If no data were excluded from the analyses, state so OR if data were excluded, provide the exact number of exclusions and the rationale behind them, indicating whether exclusion criteria were pre-established.*
Non-participation	*State how many participants dropped out/declined participation and the reason(s) given OR provide response rate OR state that no participants dropped out/declined participation.*
Randomization	*If participants were not allocated into experimental groups, state so OR describe how participants were allocated to groups, and if allocation was not random, describe how covariates were controlled.*

Ecological, evolutionary & environmental sciences study design

All studies must disclose on these points even when the disclosure is negative.

Study description	*Briefly describe the study. For quantitative data include treatment factors and interactions, design structure (e.g. factorial, nested, hierarchical), nature and number of experimental units and replicates.*
Research sample	*Describe the research sample (e.g. a group of tagged Passer domesticus, all Stenocereus thurberi within Organ Pipe Cactus National Monument), and provide a rationale for the sample choice. When relevant, describe the organism taxa, source, sex, age range and any manipulations. State what population the sample is meant to represent when applicable. For studies involving existing datasets, describe the data and its source.*
Sampling strategy	*Note the sampling procedure. Describe the statistical methods that were used to predetermine sample size OR if no sample-size calculation was performed, describe how sample sizes were chosen and provide a rationale for why these sample sizes are sufficient.*
Data collection	*Describe the data collection procedure, including who recorded the data and how.*
Timing and spatial scale	*Indicate the start and stop dates of data collection, noting the frequency and periodicity of sampling and providing a rationale for these choices. If there is a gap between collection periods, state the dates for each sample cohort. Specify the spatial scale from which the data are taken*
Data exclusions	*If no data were excluded from the analyses, state so OR if data were excluded, describe the exclusions and the rationale behind them, indicating whether exclusion criteria were pre-established.*
Reproducibility	*Describe the measures taken to verify the reproducibility of experimental findings. For each experiment, note whether any attempts to repeat the experiment failed OR state that all attempts to repeat the experiment were successful.*
Randomization	*Describe how samples/organisms/participants were allocated into groups. If allocation was not random, describe how covariates were controlled. If this is not relevant to your study, explain why.*
Blinding	*Describe the extent of blinding used during data acquisition and analysis. If blinding was not possible, describe why OR explain why blinding was not relevant to your study.*

Did the study involve field work? ☐ Yes ☐ No

Field work, collection and transport

Field conditions	*Describe the study conditions for field work, providing relevant parameters (e.g. temperature, rainfall).*
Location	*State the location of the sampling or experiment, providing relevant parameters (e.g. latitude and longitude, elevation, water depth).*
Access & import/export	*Describe the efforts you have made to access habitats and to collect and import/export your samples in a responsible manner and in compliance with local, national and international laws, noting any permits that were obtained (give the name of the issuing authority, the date of issue, and any identifying information).*
Disturbance	*Describe any disturbance caused by the study and how it was minimized.*

Reporting for specific materials, systems and methods

We require information from authors about some types of materials, experimental systems and methods used in many studies. Here, indicate whether each material, system or method listed is relevant to your study. If you are not sure if a list item applies to your research, read the appropriate section before selecting a response.

Materials & experimental systems

n/a	Involved in the study
☐	☒ Antibodies
☐	☒ Eukaryotic cell lines
☒	☐ Palaeontology and archaeology
☐	☒ Animals and other organisms
☐	☒ Human research participants
☒	☐ Clinical data
☒	☐ Dual use research of concern

Methods

n/a	Involved in the study
☒	☐ ChIP-seq
☐	☒ Flow cytometry
☒	☐ MRI-based neuroimaging

Antibodies

Antibodies used

Abbreviation: Immunohistochemistry (IHC), Weston blot (WB), Immunofluorescence (IF), Flow Cytometry (FC), Immunoprecipitation (IP). The antibodies' supplier name, catalog number etc. and dilution of each application were listed as below:

Anti-alpha smooth muscle Actin[1A4], abcam, ab7817, IHC-P (0.034 µg/ml), WB (0.341 µg/ml), IF (1:100)
Anti-wide spectrum Cytokeratin , abcam, ab9377, IHC-P (1:100), WB (1:1000)
Donkey anti-Rabbit IgG (H+L) Highly Cross-Adsorbed Secondary Antibody, Alexa Fluor 488 , life , A-21206 , IF (1:300)
Donkey anti-Mouse IgG (H+L) Highly Cross-Adsorbed Secondary Antibody, Alexa Fluor Plus 594, life, A32744, IF (1:300)
Anti-Oxytocin Receptor , abcam , ab87312, WB (1:500)
Oxytocin Receptor Antibody, proteintech, 23045-1-AP, FC (0.2 ug/sample)
Anti-Caveolin-1[7C8], abcam, ab17052, WB (1:50)
OXTR Polyclonal Antibody (Lot:B7201, B72RC9) , ImmunoWay , YN2672, WB (1:1000), IHC (1:10000)
Rabbit Anti-OXTR, NT (OXTR, Oxytocin receptor) (APC), Usbio, 039556-APC, FC (2ul/sample)
Anti-Oxytocin Receptor[EPR12789], abcam, ab181077, IF (1:200)
Anti-Caveolin-1[E249] - Caveolae Marker, abcam, ab32577, WB (1:1000)
Phospho-p44/42 MAPK (Erk1/2) (Thr202/Tyr204) (D13.14.4E) XP Rabbit mAb, CST, 4370T, IF (1:200), WB (1:1000)
Alexa Fluor 647-AffiniPure Goat Anti-Rabbit IgG (H+L), Jackson, 111-605-003, IF (1:500)
Anti-GNAQ, abcam, ab75825, WB (1 µg/ml), IP (1:50)
Anti-CD146[EPR3208], abcam, ab75769, IHC (1:250)
Anti-FOXP3[EPR22102-37] , abcam, ab215206, IHC (1:250)
Anti-CCR3[Y31], abcam, ab32512, IHC (1:100), WB (1:500)
Phospho-c-Jun (Ser73) (D47G9) XP Rabbit mAb , CST, 3270T, IF (1:500), WB (1:1000)
Erk5 (D3I5V) Rabbit mAb , CST, 12950S, IP (1:50), WB (1:1000)
Phospho-Erk5 (Thr218/Tyr220) Antibody , CST , 3371S, WB (1:1000)
Phospho-c-Fos (Ser32) (D82C12) XP Rabbit mAb, CST, 5348T, IF (1:200), WB (1:1000)
Anti-MEK5 (phospho S311 + T315), abcam, ab254134, WB (1:1000)
Anti-MEK5, abcam, ab210748, IF (1:100), WB (1:1000)
c-Myc Antibody, CST, 9402S, WB (1:1000)
PDGF Receptor β (28E1) Rabbit mAb, CST, 3169S, IF (1:100), WB (1:1000)
Anti-MEF2C[EPR19089-202] - ChIP Grade, abcam , ab211493, WB (1:1000)
CD29-PE, human 30 tests, miltenyi, 130-101-275, FC (1ul/sample)
Lamin A/C (4C11) Mouse mAb, CST, 4777T, WB (1:1000)
Phospho-c-Fos (Ser32) (D82C12) XP Rabbit mAb, CST, 5348S, IF (1:200), WB (1:1000)
ERK 5 antibody (C-7): Santacruz s, c-398015, IF (1:50)
β-Arrestin 1/2 (D24H9) Rabbit mAb, CST, 4674s, WB (1:1000), IP (1:100)
Anti-Beta Arrestin 1 and 2, abcam , ab32099 and 54790, WB (1:1000)
SUMO-2/3 (18H8) Rabbit mAb , CST , 4971T, WB (1:1000)
HSP90 (C45G5) Rabbit mAb, CST, 4877T, WB (1:1000)
CDC37 (D11A3) XP Rabbit mAb , CST , 4793S, WB (1:1000)
c-Fos (phospho Ser374) Polyclonal Antibody, ImmunoWay, YP1095, WB (1:1000)
Human FAP Phycoerythrin MAb (Clone 427819) (25 TESTS), R&D , FAB3715P-025, FC (2ul/sample)
AVP Receptor V3 Polyclonal Antibody , ImmunoWay, YT6001, WB (1:1000)
AVP Receptor V2 Polyclonal Antibody, ImmunoWay, YT0423, WB (1:1000)
AVPR1A Polyclonal Antibody, ImmunoWay, YT6097, WB (1:1000)
PKC ζ Polyclonal Antibody, ImmunoWay , YT3765, WB (1:1000)
PKC ζ (phospho Thr560) Polyclonal Antibody, ImmunoWay, YP0230, WB (1:1000)
Epithelial-Mesenchymal Transition (EMT) IF Antibody Sampler Kit , Cell Signaling , 49398, WB (1:500-1000)
ECM Profiling Sampler Kit, Cell Signaling , 33437, IF (1:100-400)
APC anti-human CD31, BioLegend, 303115, FC (2ul/sample)
APC anti-human CD326 (EpCAM), BioLegend , 369809, FC (1.5ul/sample)
APC anti-human CD45, BioLegend, 368511, FC (2ul/sample)
CD4 , Zhong Shan -Golden Bridge , ZM-0418, IHC (ready-to-use)
CD8 , Zhong Shan -Golden Bridge, ZA-0508, IHC (ready-to-use)

CD68, Zhong Shan -Golden Bridge, ZM-0464, IHC (ready-to-use)
Collagen Ⅰ, Zhong Shan -Golden Bridge , ZA-0616, IHC (ready-to-use)
MMP-9, Zhong Shan -Golden Bridge , ZA-0562, IHC (ready-to-use)
Anti-TSC-1 (CCL-26) , abcam , ab217328, WB (0.5 µg/ml)

Validation

All antibodies were validated by the supplier and their validation data are available on the manufacturer's website:

Anti-alpha smooth muscle Actin[1A4], abcam, ab7817, https://www.abcam.cn/alpha-smooth-muscle-actin-antibody-1a4-ab7817.html.

Anti-wide spectrum Cytokeratin , abcam, ab9377, https://www.abcam.cn/wide-spectrum-cytokeratin-antibody-ab9377.html.

Donkey anti-Rabbit IgG (H+L) Highly Cross-Adsorbed Secondary Antibody, Alexa Fluor 488 , life , A-21206 ,https://www.thermofisher.cn/cn/zh/antibody/product/Donkey-anti-Rabbit-IgG-H-L-Highly-Cross-Adsorbed-Secondary-Antibody-Polyclonal/A-21206

Donkey anti-Mouse IgG (H+L) Highly Cross-Adsorbed Secondary Antibody, Alexa Fluor Plus 594, life, A32744, https://www.thermofisher.cn/cn/zh/antibody/product/Donkey-anti-Mouse-IgG-H-L-Highly-Cross-Adsorbed-Secondary-Antibody-Polyclonal/A32744

Anti-Oxytocin Receptor , abcam , ab87312, https://www.abcam.cn/oxytocin-receptor-antibody-ab87312.html

Oxytocin Receptor Antibody, proteintech, 23045-1-AP, https://www.ptglab.com/Products/OXTR-Antibody-23045-1-AP.htm

Anti-Caveolin-1[7C8], abcam, ab17052, https://www.abcam.cn/caveolin-1-antibody-7c8-ab17052.html

OXTR Polyclonal Antibody (Lot:B7201, B72RC9) , ImmunoWay , YN2672, http://www.immunoway.com/Home/22/YN2672

Rabbit Anti-OXTR, NT (OXTR, Oxytocin receptor) (APC), Usbio, 039556-APC, https://www.usbio.net/antibodies/039556-APC/oxtr-nt-oxtr-oxytocin-receptor-apc

Anti-Oxytocin Receptor[EPR12789], abcam, ab181077, https://www.abcam.cn/oxytocin-receptor-antibody-epr12789-ab181077.html

Anti-Caveolin-1[E249] - Caveolae Marker, abcam, ab32577, https://www.abcam.cn/caveolin-1-antibody-e249-caveolae-marker-ab32577.html

Phospho-p44/42 MAPK (Erk1/2) (Thr202/Tyr204) (D13.14.4E) XP Rabbit mAb, CST, 4370T, https://www.cellsignal.cn/products/primary-antibodies/phospho-p44-42-mapk-erk1-2-thr202-tyr204-d13-14-4e-xp-rabbit-mab/4370?site-search-type=Products&N=4294956287&Ntt=4370t&fromPage=plp&_requestid=1162934

Alexa Fluor 647-AffiniPure Goat Anti-Rabbit IgG (H+L), Jackson, 111-605-003, https://www.jacksonimmuno.com/catalog/products/111-605-003

Anti-GNAQ, abcam, ab75825, https://www.abcam.cn/gnaq-antibody-ab75825.html

Anti-CD146[EPR3208], abcam, ab75769, https://www.abcam.cn/cd146-antibody-epr3208-ab75769.html

Anti-FOXP3[EPR22102-37] , abcam, ab215206, https://www.abcam.cn/foxp3-antibody-epr22102-37-ab215206.html

Anti-CCR3[Y31], abcam, ab32512, https://www.abcam.cn/ccr3-antibody-y31-ab32512.html

Phospho-c-Jun (Ser73) (D47G9) XP Rabbit mAb , CST, 3270T, https://www.cellsignal.cn/products/primary-antibodies/phospho-c-jun-ser73-d47g9-xp-rabbit-mab/3270?site-search-type=Products&N=4294956287&Ntt=3270&fromPage=plp&_requestid=1163612

Erk5 (D3I5V) Rabbit mAb , CST, 12950S, https://www.cellsignal.cn/products/primary-antibodies/erk5-d3i5v-rabbit-mab/12950?site-search-type=Products&N=4294956287&Ntt=12950s&fromPage=plp&_requestid=1163686

Phospho-Erk5 (Thr218/Tyr220) Antibody , CST , 3371S, https://www.cellsignal.cn/products/primary-antibodies/phospho-erk5-thr218-tyr220-antibody/3371?site-search-type=Products&N=4294956287&Ntt=3371s&fromPage=plp&_requestid=1163783

Phospho-c-Fos (Ser32) (D82C12) XP Rabbit mAb, CST, 5348T, https://www.cellsignal.cn/products/primary-antibodies/phospho-c-fos-ser32-d82c12-xp-rabbit-mab/5348?site-search-type=Products&N=4294956287&Ntt=5348t&fromPage=plp&_requestid=1163849

Anti-MEK5 (phospho S311 + T315), abcam, ab254134, https://www.abcam.cn/mek5-phospho-s311--t315-antibody-ab254134.html

Anti-MEK5, abcam, ab210748, https://www.abcam.cn/mek5-antibody-ab210748.html

c-Myc Antibody, CST, 9402S, https://www.cellsignal.cn/products/primary-antibodies/c-myc-antibody/9402?site-search-type=Products&N=4294956287&Ntt=9402s&fromPage=plp&_requestid=1164112

PDGF Receptor β (28E1) Rabbit mAb, CST, 3169S, https://www.cellsignal.cn/products/primary-antibodies/pdgf-receptor-b-28e1-rabbit-mab/3169?site-search-type=Products&N=4294956287&Ntt=3169s&fromPage=plp&_requestid=1164185

Anti-MEF2C[EPR19089-202] - ChIP Grade, abcam , ab211493, https://www.abcam.cn/mef2c-antibody-epr19089-202-chip-grade-ab211493.html

CD29-PE, human 30 tests, miltenyi, 130-101-275, https://www.miltenyibiotec.com/CN-en/search.html?search=130-101-275

Lamin A/C (4C11) Mouse mAb, CST, 4777T, https://www.cellsignal.cn/products/primary-antibodies/lamin-a-c-4c11-mouse-mab/4777?site-search-type=Products&N=4294956287&Ntt=4777t&fromPage=plp&_requestid=1164587

Phospho-c-Fos (Ser32) (D82C12) XP Rabbit mAb, CST, 5348, https://www.cellsignal.cn/products/primary-antibodies/phospho-c-fos-ser32-d82c12-xp-rabbit-mab/5348?site-search-type=Products&N=4294956287&Ntt=5348s&fromPage=plp&_requestid=1164674

ERK 5 antibody (C-7): Santacruz s, c-398015 ,https://www.scbt.com/zh/p/erk-5-antibody-c-7;jsessionid=wpJYQYdowTN6BOIaEkhOsdVCdr-ldedjJhUAeHMmRzVZMPoWENZZ!-2089528274

β-Arrestin 1/2 (D24H9) Rabbit mAb, CST, 4674s, https://www.cellsignal.cn/products/primary-antibodies/b-arrestin-1-2-d24h9-rabbit-mab/4674?site-search-type=Products&N=4294956287&Ntt=4674s&fromPage=plp&_requestid=1164881

Anti-Beta Arrestin 1, abcam , ab32099, https://www.abcam.cn/nav/primary-antibodies/rabbit-monoclonal-antibodies/beta-arrestin-1-antibody-e274-ab32099.html

Anti-Beta Arrestin 2, abcam , ab54790, https://www.abcam.cn/beta-arrestin-2-antibody-3g1-ab54790.html

SUMO-2/3 (18H8) Rabbit mAb , CST , 4971T, https://www.cellsignal.cn/products/primary-antibodies/sumo-2-3-18h8-rabbit-mab/4971?site-search-type=Products&N=4294956287&Ntt=4971t&fromPage=plp&_requestid=1165883

HSP90 (C45G5) Rabbit mAb, CST, 4877T, https://www.cellsignal.cn/products/primary-antibodies/hsp90-c45g5-rabbit-mab/4877?site-search-type=Products&N=4294956287&Ntt=4877t&fromPage=plp&_requestid=1165946

CDC37 (D11A3) XP Rabbit mAb , CST , 4793S, https://www.cellsignal.cn/products/primary-antibodies/cdc37-d11a3-xp-rabbit-mab/4793?site-search-type=Products&N=4294956287&Ntt=4793s&fromPage=plp&_requestid=1165997

c-Fos (phospho Ser374) Polyclonal Antibody, ImmunoWay, YP1095, http://www.immunoway.com/Home/22/YP1095

Human FAP Phycoerythrin MAb (Clone 427819) (25 TESTS), R&D , FAB3715P-025, https://www.rndsystems.com/cn/products/human-fibroblast-activation-protein-alpha-fap-pe-conjugated-antibody-427819_fab3715p

AVP Receptor V3 Polyclonal Antibody , ImmunoWay, YT6001, http://www.immunoway.com/Home/22/YT6001

AVP Receptor V2 Polyclonal Antibody, ImmunoWay, YT0423, http://www.immunoway.com/Home/22/YT0423

AVPR1A Polyclonal Antibody, ImmunoWay, YT6097, http://www.immunoway.com/Home/22/YT6097

PKC ζ Polyclonal Antibody, ImmunoWay , YT3765, http://www.immunoway.com/Home/22/YT3765

PKC ζ (phospho Thr560) Polyclonal Antibody, ImmunoWay, YP0230, http://www.immunoway.com/Home/22/YP0230

Epithelial-Mesenchymal Transition (EMT) IF Antibody Sampler Kit , Cell Signaling , 49398, https://www.cellsignal.cn/products/primary-antibodies/epithelial-mesenchymal-transition-emt-if-antibody-sampler-kit/49398?site-search-type=Products&N=4294956287&Ntt=49398&fromPage=plp&_requestid=1166610

ECM Profiling Sampler Kit, Cell Signaling , 33437, https://www.cellsignal.cn/products/primary-antibodies/ecm-profiling-antibody-sampler-kit/33437?site-search-type=Products&N=4294956287&Ntt=33437&fromPage=plp&_requestid=1166681

APC anti-human CD31, BioLegend, 303115, https://www.biolegend.com/en-us/products/apc-anti-human-cd31-antibody-6123

APC anti-human CD326 (EpCAM), BioLegend , 369809, https://www.biolegend.com/en-us/products/apc-anti-human-

cd326-epcam-antibody-14168

APC anti-human CD45, BioLegend, 368511, https://www.biolegend.com/en-us/products/apc-anti-human-cd45-antibody-12397

CD4 , Zhong Shan -Golden Bridge , ZM-0418, http://www.zsbio.com/product/ZM-0418

CD8 , Zhong Shan -Golden Bridge, ZA-0508, http://www.zsbio.com/product/ZM-0508

CD68, Zhong Shan -Golden Bridge, ZM-0464, http://www.zsbio.com/product/ZM-0464

Collagen Ⅰ, Zhong Shan -Golden Bridge , ZA-0616,http://www.zsbio.com/product/ZA-0616

MMP-9, Zhong Shan -Golden Bridge , ZA-0562, http://www.zsbio.com/product/ZA-0562

Anti-TSC-1 (CCL-26) , abcam , ab217328, https://www.abcam.cn/tsc-1-antibody-ab217328.html

Eukaryotic cell lines

Policy information about cell lines

Cell line source(s)	1. Patient-Derived CAF cell lines were derived from primary OSCC patients through a discarded tissue protocol, in accordance with Ethics Committee of Nanjing Stomatology Hospital (No.2019NL-009(KS)).<br>2. The human OSCC cell line HSC-3 (JCRB0623) was obtained from the Human Science Research Resources Bank, Japan Health Sciences Foundation (Tokyo, Japan). The SCC7 cell line (RRID: CVCL_V412) was obtained from a squamous carcinoma that arose spontaneously in the abdominal wall of a C3H mouse. HN6 was obtained from tongue squamous cell carcinoma patients. HN6 and SCC7 were gifted by Professor Laiping Zhong (Shanghai Jiao Tong University, SJTU).
Authentication	Both cell lines were authenticated using Short Tandem Repeat (STR) analysis as described (2012 in ANSI Standard (ASN-0002)) by the ATCC Standards Development Organization (SDO).
Mycoplasma contamination	The cells had been characterized for mycoplasma detection, DNA fingerprinting, isozyme detection and cell viability by the providers. No presence of mycoplasma was found according to Mycoplasma Detection Kit-QuickTest from Biomake (Houston, TX, USA)
Commonly misidentified lines<br>(See ICLAC register)	None

Palaeontology and Archaeology

Specimen provenance	*Provide provenance information for specimens and describe permits that were obtained for the work (including the name of the issuing authority, the date of issue, and any identifying information). Permits should encompass collection and, where applicable, export.*
Specimen deposition	*Indicate where the specimens have been deposited to permit free access by other researchers.*
Dating methods	*If new dates are provided, describe how they were obtained (e.g. collection, storage, sample pretreatment and measurement), where they were obtained (i.e. lab name), the calibration program and the protocol for quality assurance OR state that no new dates are provided.*

☐ Tick this box to confirm that the raw and calibrated dates are available in the paper or in Supplementary Information.

Ethics oversight	*Identify the organization(s) that approved or provided guidance on the study protocol, OR state that no ethical approval or guidance was required and explain why not.*

Note that full information on the approval of the study protocol must also be provided in the manuscript.

Animals and other organisms

Policy information about studies involving animals; ARRIVE guidelines recommended for reporting animal research

Laboratory animals	NCG triple immunodeficient mice, lacking T, B and NK cells (NOD/ShiLtJGpt-Prkdcem26Cd52Il2rgem26Cd22/Gpt, from GemPharmatech Co. Ltd., Nanjing, China)were used (both genders) for the orthotopic xenograft at 4-6 weeks of age and housed in ultraclean barrier facilities. Both gender of Fsp1-Cre mice and the Oxtr-flox/flox mouse strain was established in the Genetically Engineered Mouse Facility at GemPharmatech Co. Ltd. n(Nanjing, China). The Oxtrfl/fl S100a4cre mouse strain was generated by crossing the Fsp1-Cre (B6/JGpt-Tg(S100a4-CreERT2-PolyA)3/Gpt) and Oxtrflox/flox mouse strains (B6/JGpt-Oxtrem1Cflox/Gpt) with genotypic and phenotypic characterization. The housing conditions for the mice were as follows: 12h light/12 h darkness; temperature was 72 degrees Fahrenheit; and humidity was 40-50%.<br>Mice were sacrificed by cervical dislocation at the indicated time points or when the largest tumor exceeded 1500 mm3 in subcutaneous models or 40 mm3 in orthotopic model (no mice exceeded these limits). In some cases, this limit has been reached the

	last day of measurement and the mice were immediately euthanized. All animal experiments were carried out following animal protocols approved by the Laboratory Animal Welfare and Ethics Committee of NanJing university.
Wild animals	None
Field-collected samples	None
Ethics oversight	All animal experiments were performed in accordance with Jiangsu Association for Laboratory Animal Science (Authorization Number: 220195073) and were subject to review by the animal welfare and ethical review board of the University of NanJing university.

Note that full information on the approval of the study protocol must also be provided in the manuscript.

Human research participants

Policy information about studies involving human research participants

Population characteristics	All male and female patients (26-88 year old, Asian) in this study diagnosed with primary OSCC were confirmed by hematoxylin and eosin staining of tumour biopsy.
Recruitment	There were no self-selection bias or other biases. Participants were recruited for blood and tissue donation for research purpose, under informed consent.
Ethics oversight	Ethical approval for this study including tumor biopsy and serum collection was obtained from the Research Ethics Committee of Nanjing Stomatology Hospital (No.2019NL-009(KS)). All patients gave informed consents for collection of tissue collection. The study was conducted in accordance with the criteria set by the Declaration of Helsinki.

Note that full information on the approval of the study protocol must also be provided in the manuscript.

Clinical data

Policy information about clinical studies

All manuscripts should comply with the ICMJEguidelines for publication of clinical research and a completedCONSORT checklist must be included with all submissions.

Clinical trial registration	*Provide the trial registration number from ClinicalTrials.gov or an equivalent agency.*
Study protocol	*Note where the full trial protocol can be accessed OR if not available, explain why.*
Data collection	*Describe the settings and locales of data collection, noting the time periods of recruitment and data collection.*
Outcomes	*Describe how you pre-defined primary and secondary outcome measures and how you assessed these measures.*

Dual use research of concern

Policy information about dual use research of concern

Hazards

Could the accidental, deliberate or reckless misuse of agents or technologies generated in the work, or the application of information presented in the manuscript, pose a threat to:

No	Yes	
☐	☐	Public health
☐	☐	National security
☐	☐	Crops and/or livestock
☐	☐	Ecosystems
☐	☐	Any other significant area

Experiments of concern

Does the work involve any of these experiments of concern:

No	Yes	
☐	☐	Demonstrate how to render a vaccine ineffective
☐	☐	Confer resistance to therapeutically useful antibiotics or antiviral agents
☐	☐	Enhance the virulence of a pathogen or render a nonpathogen virulent
☐	☐	Increase transmissibility of a pathogen
☐	☐	Alter the host range of a pathogen
☐	☐	Enable evasion of diagnostic/detection modalities
☐	☐	Enable the weaponization of a biological agent or toxin
☐	☐	Any other potentially harmful combination of experiments and agents

ChIP-seq

Data deposition

☐ Confirm that both raw and final processed data have been deposited in a public database such as GEO.

☐ Confirm that you have deposited or provided access to graph files (e.g. BED files) for the called peaks.

Data access links<br>*May remain private before publication.*	*For "Initial submission" or "Revised version" documents, provide reviewer access links. For your "Final submission" document, provide a link to the deposited data.*
Files in database submission	*Provide a list of all files available in the database submission.*
Genome browser session<br>(e.g. UCSC)	*Provide a link to an anonymized genome browser session for "Initial submission" and "Revised version" documents only, to enable peer review. Write "no longer applicable" for "Final submission" documents.*

Methodology

Replicates	*Describe the experimental replicates, specifying number, type and replicate agreement.*
Sequencing depth	*Describe the sequencing depth for each experiment, providing the total number of reads, uniquely mapped reads, length of reads and whether they were paired- or single-end.*
Antibodies	*Describe the antibodies used for the ChIP-seq experiments; as applicable, provide supplier name, catalog number, clone name, and lot number.*
Peak calling parameters	*Specify the command line program and parameters used for read mapping and peak calling, including the ChIP, control and index files used.*
Data quality	*Describe the methods used to ensure data quality in full detail, including how many peaks are at FDR 5% and above 5-fold enrichment.*
Software	*Describe the software used to collect and analyze the ChIP-seq data. For custom code that has been deposited into a community repository, provide accession details.*

Flow Cytometry

Plots

Confirm that:

☒ The axis labels state the marker and fluorochrome used (e.g. CD4-FITC).

☒ The axis scales are clearly visible. Include numbers along axes only for bottom left plot of group (a 'group' is an analysis of identical markers).

☒ All plots are contour plots with outliers or pseudocolor plots.

☒ A numerical value for number of cells or percentage (with statistics) is provided.

Methodology

Sample preparation	To compare CAF phenotypes in different groups, cells were resuspended in PBS containing 1% FBS and stained with fluorescent-conjugated antibodies to detect CD29, FAP or PDGFR-β. For cell sorting in OSCC tissues, single cell suspensions were prepared from OSCC tissues via brief trypsinization. This was followed by forward and side scatter flow cytometry gating to exclude cell debris and dead cells by DAPI positive staining. Cells were stained with fluorescent-conjugated antibodies to detect EpCAM, CD45, CD31, PDGFR-β or FAP positive cell subpopulations. To facilitate isolation of OXTR high or low-

	expressing CAFs, live single cells were labelled with APC/PE-conjugated anti-OXTR and sorted according to the OXTR fluorescence intensity, which was verified at the protein level by Western blot.
Instrument	SONY SH800, BD calibur
Software	FlowJo 10.0
Cell population abundance	1. Analysis on CAF phenotypes, all the CAFs show 100% positivity for CD29, FAP or PDGFR-β;<br>2. To facilitate sorting of OXTR high or low-expressing CAFs, OXTR expression <10% were isolated as OXTR-low and >90% as OXTR-high. All cells were sorted at >=90% purity.
Gating strategy	Gating strategy was devised manually and appropriate to experiments according to Isotype Control.

☒ Tick this box to confirm that a figure exemplifying the gating strategy is provided in the Supplementary Information.

Magnetic resonance imaging

Experimental design

Design type	*Indicate task or resting state; event-related or block design.*
Design specifications	*Specify the number of blocks, trials or experimental units per session and/or subject, and specify the length of each trial or block (if trials are blocked) and interval between trials.*
Behavioral performance measures	*State number and/or type of variables recorded (e.g. correct button press, response time) and what statistics were used to establish that the subjects were performing the task as expected (e.g. mean, range, and/or standard deviation across subjects).*

Acquisition

Imaging type(s)	*Specify: functional, structural, diffusion, perfusion.*
Field strength	*Specify in Tesla*
Sequence & imaging parameters	*Specify the pulse sequence type (gradient echo, spin echo, etc.), imaging type (EPI, spiral, etc.), field of view, matrix size, slice thickness, orientation and TE/TR/flip angle.*
Area of acquisition	*State whether a whole brain scan was used OR define the area of acquisition, describing how the region was determined.*

Diffusion MRI ☐ Used ☐ Not used

Preprocessing

Preprocessing software	*Provide detail on software version and revision number and on specific parameters (model/functions, brain extraction, segmentation, smoothing kernel size, etc.).*
Normalization	*If data were normalized/standardized, describe the approach(es): specify linear or non-linear and define image types used for transformation OR indicate that data were not normalized and explain rationale for lack of normalization.*
Normalization template	*Describe the template used for normalization/transformation, specifying subject space or group standardized space (e.g. original Talairach, MNI305, ICBM152) OR indicate that the data were not normalized.*
Noise and artifact removal	*Describe your procedure(s) for artifact and structured noise removal, specifying motion parameters, tissue signals and physiological signals (heart rate, respiration).*
Volume censoring	*Define your software and/or method and criteria for volume censoring, and state the extent of such censoring.*

Statistical modeling & inference

Model type and settings	*Specify type (mass univariate, multivariate, RSA, predictive, etc.) and describe essential details of the model at the first and second levels (e.g. fixed, random or mixed effects; drift or auto-correlation).*
Effect(s) tested	*Define precise effect in terms of the task or stimulus conditions instead of psychological concepts and indicate whether ANOVA or factorial designs were used.*

Specify type of analysis: ☐ Whole brain ☐ ROI-based ☐ Both

Statistic type for inference<br>(See Eklund et al. 2016)	*Specify voxel-wise or cluster-wise and report all relevant parameters for cluster-wise methods.*
Correction	*Describe the type of correction and how it is obtained for multiple comparisons (e.g. FWE, FDR, permutation or Monte Carlo).*

Models & analysis

n/a	Involved in the study
☐	☐ Functional and/or effective connectivity
☐	☐ Graph analysis
☐	☐ Multivariate modeling or predictive analysis

Functional and/or effective connectivity	*Report the measures of dependence used and the model details (e.g. Pearson correlation, partial correlation, mutual information).*
Graph analysis	*Report the dependent variable and connectivity measure, specifying weighted graph or binarized graph, subject- or group-level, and the global and/or node summaries used (e.g. clustering coefficient, efficiency, etc.).*
Multivariate modeling and predictive analysis	*Specify independent variables, features extraction and dimension reduction, model, training and evaluation metrics.*
