# Supplementary figures and images for: OXTRHigh stroma fibroblasts control the invasion pattern of oral squamous cell carcinoma via ERK5 signaling
Source: Nat Commun. 2022 Aug 31;13:5124. doi: 10.1038/s41467-022-32787-y (PMC9433374; doi:10.1038/s41467-022-32787-y)

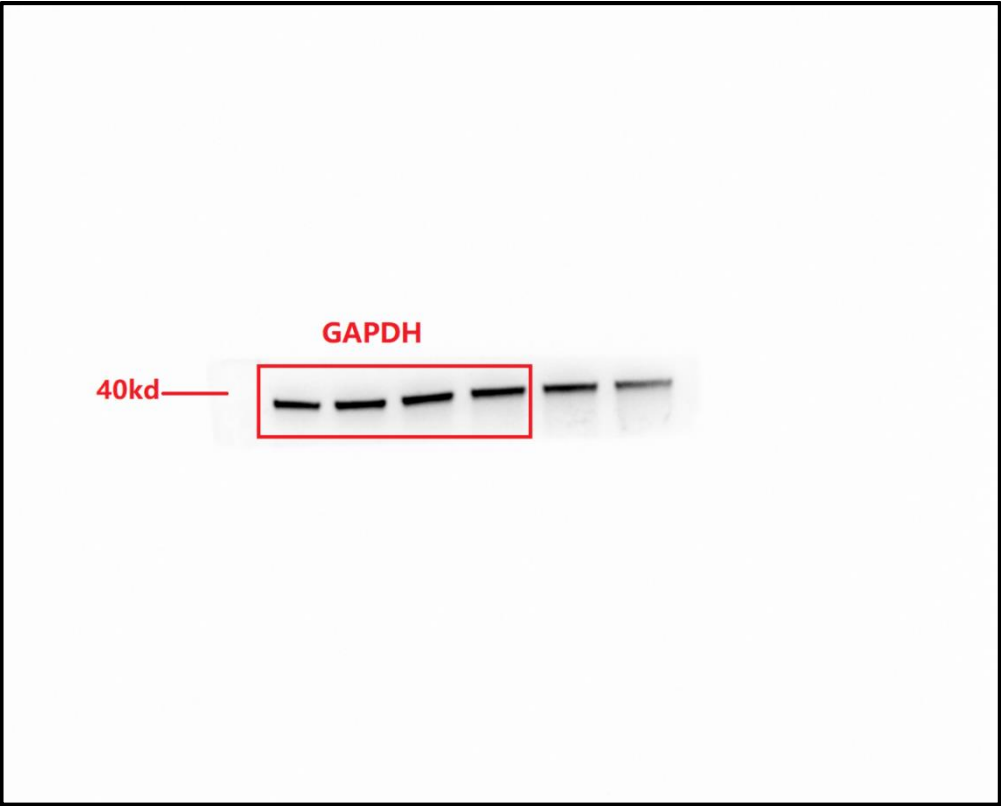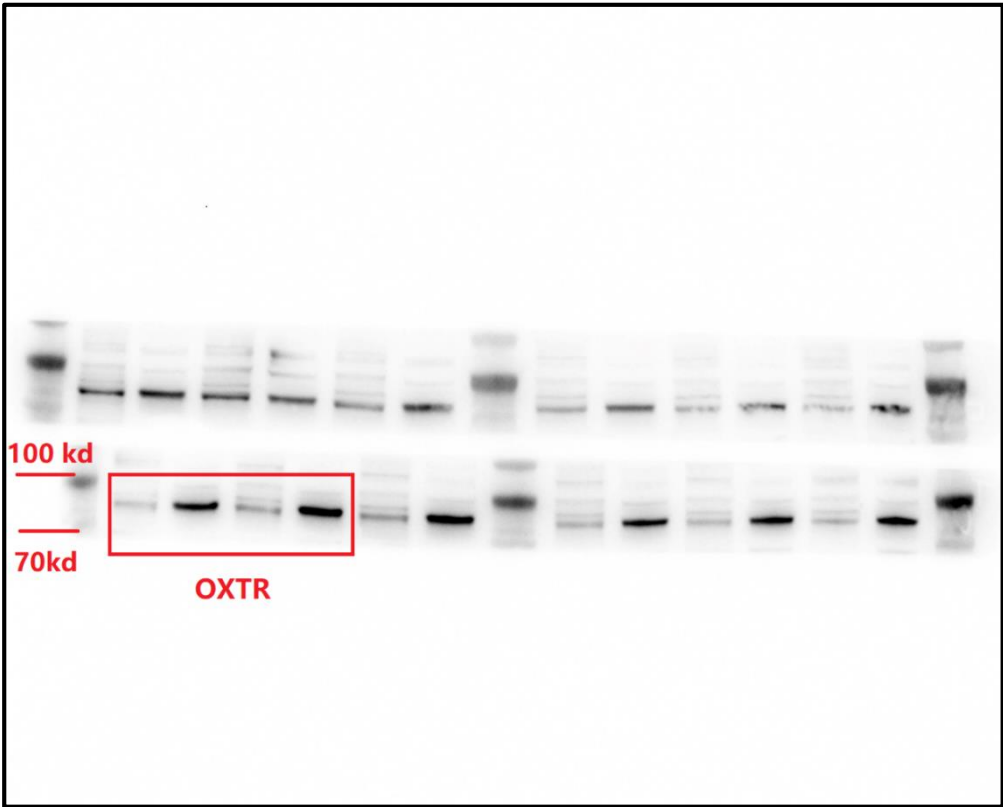

Supplement: Supplementary file 4 — Source Data [file 41467_2022_32787_MOESM4_ESM.zip › Source Data/Figure 3/Figure 3b.pdf]

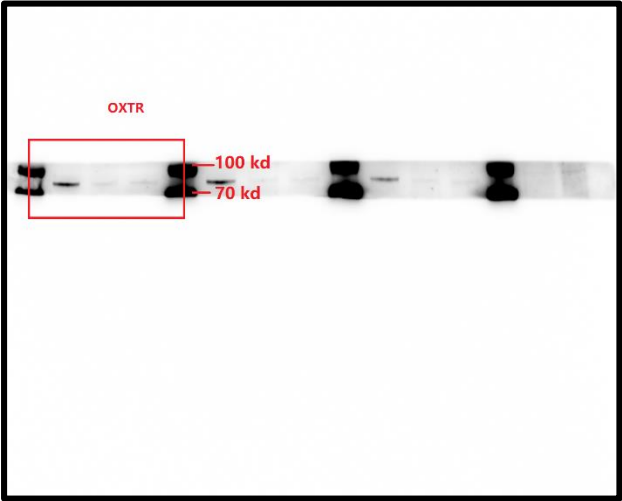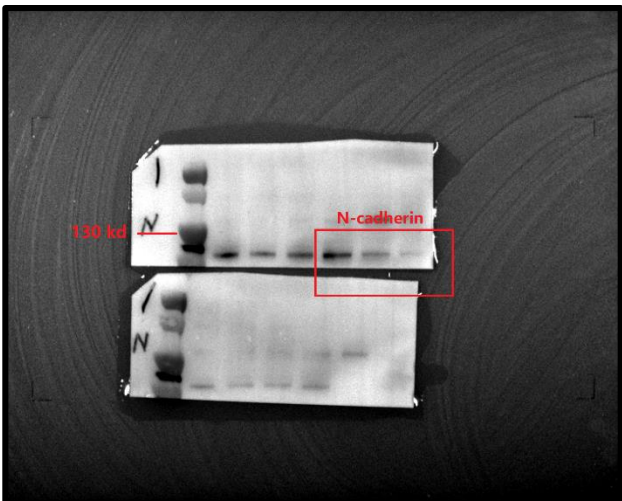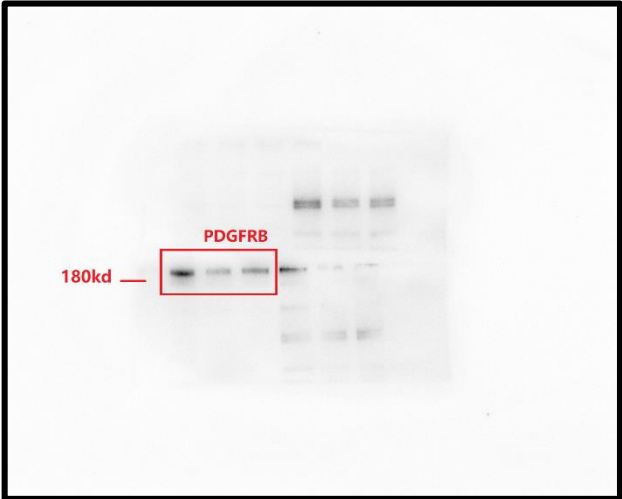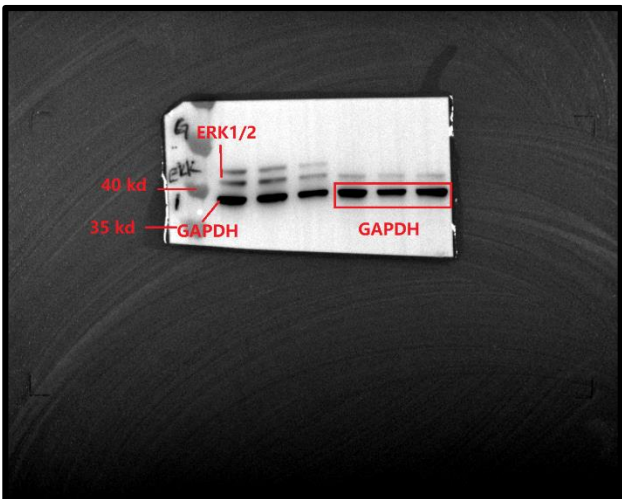

Supplement: Supplementary file 4 — Source Data [file 41467_2022_32787_MOESM4_ESM.zip › Source Data/Figure 4/Figure 4l.pdf]

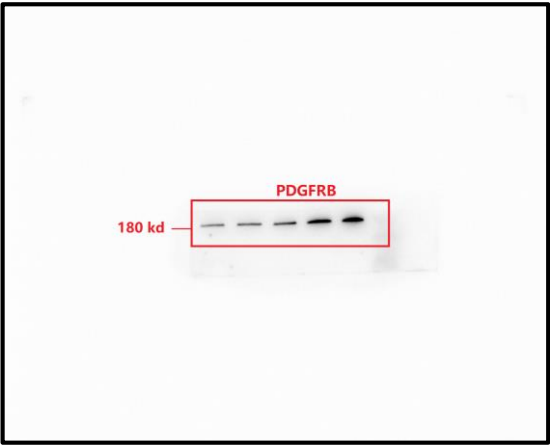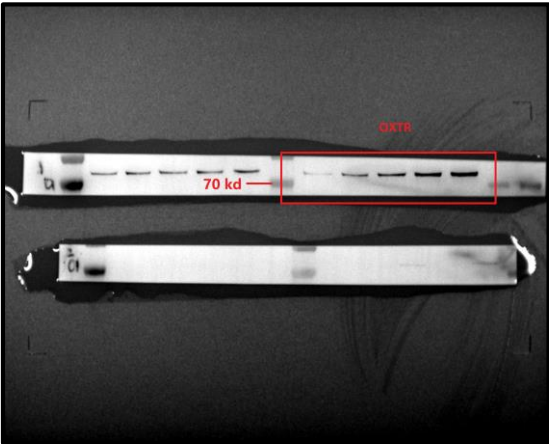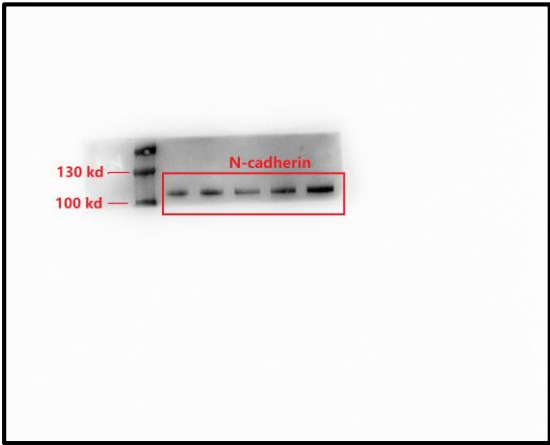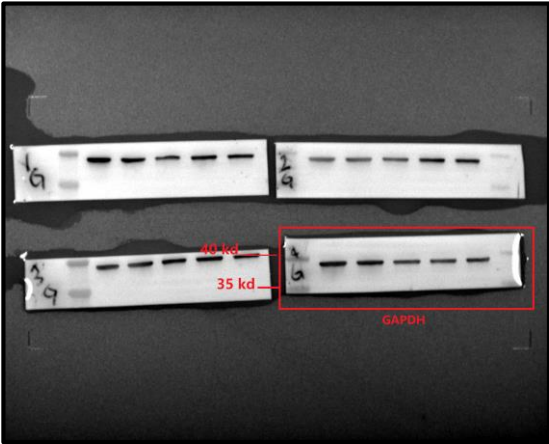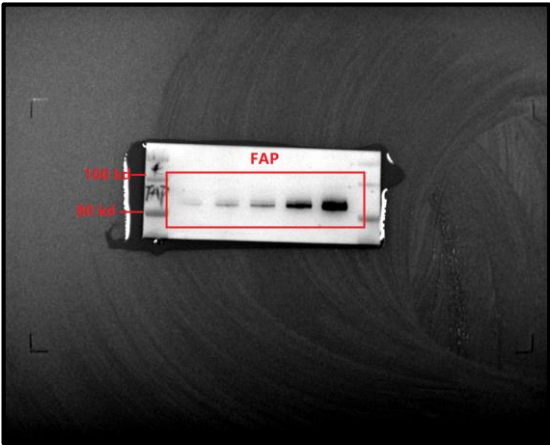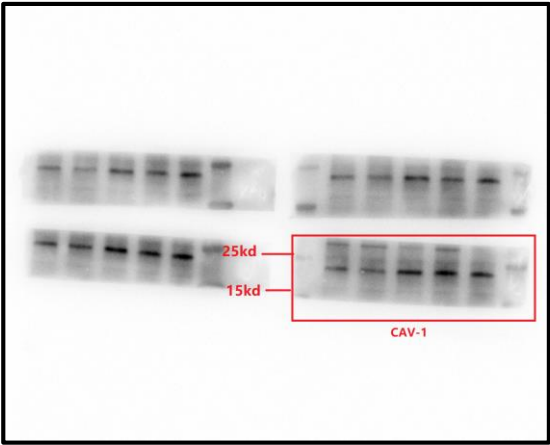

Supplement: Supplementary file 4 — Source Data [file 41467_2022_32787_MOESM4_ESM.zip › Source Data/Figure 6/Figure 6b.pdf]

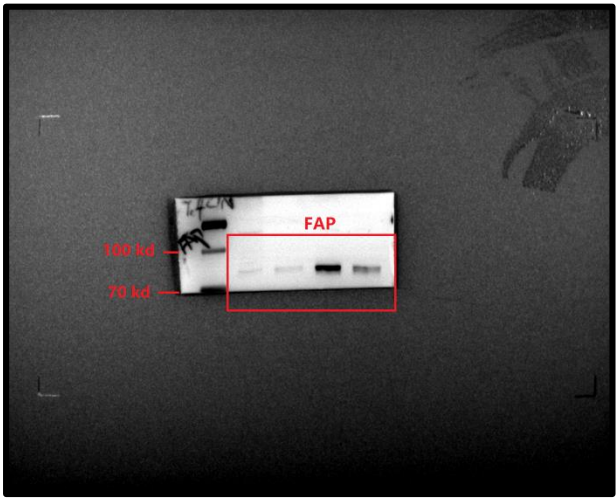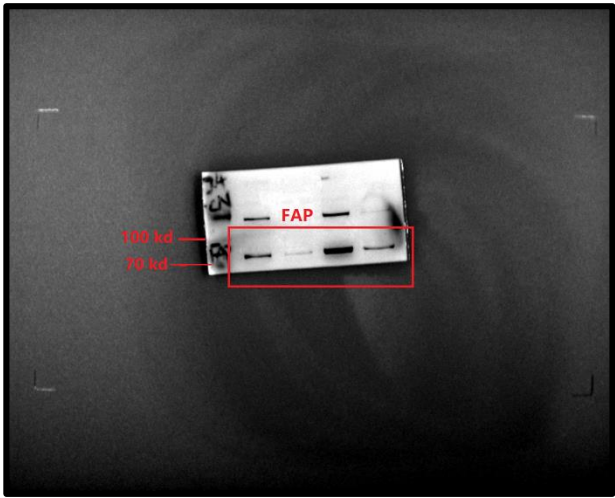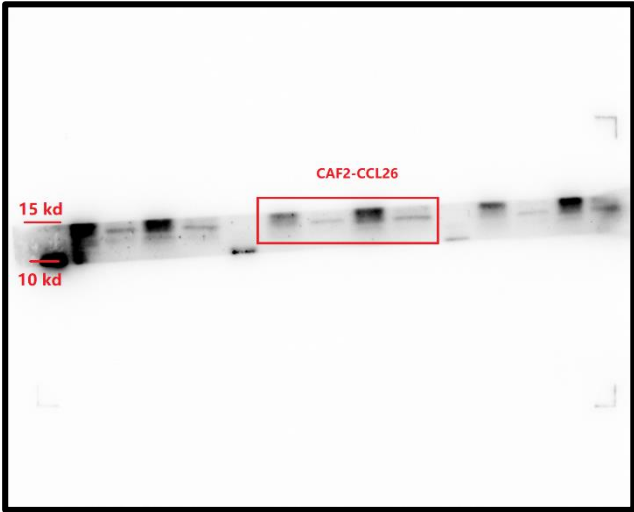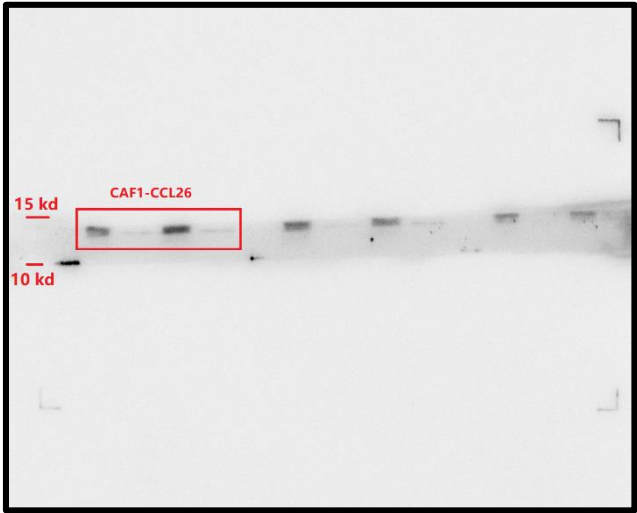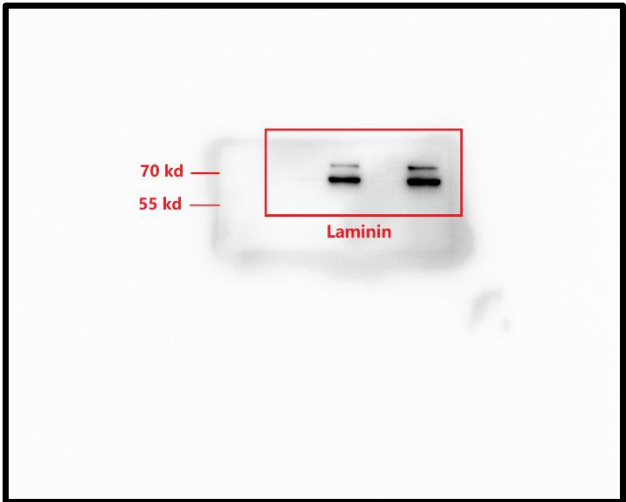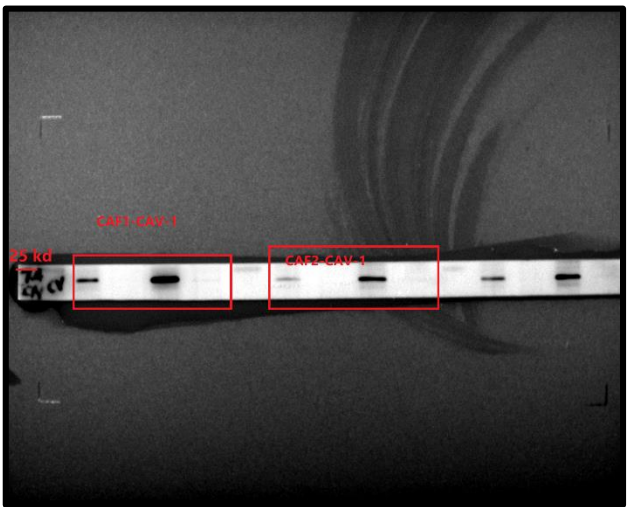

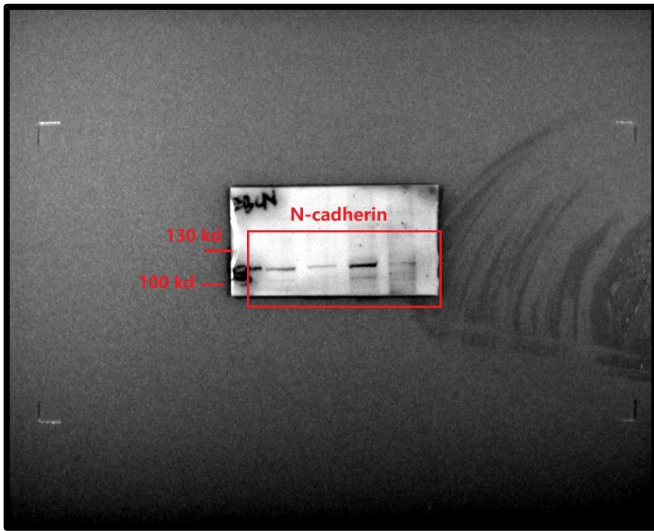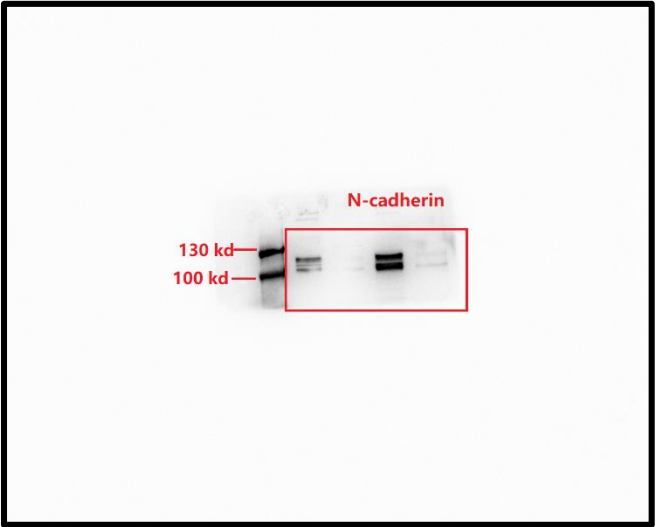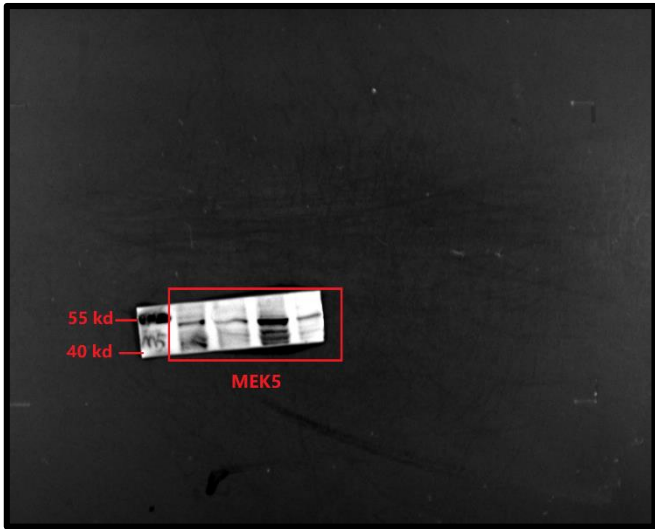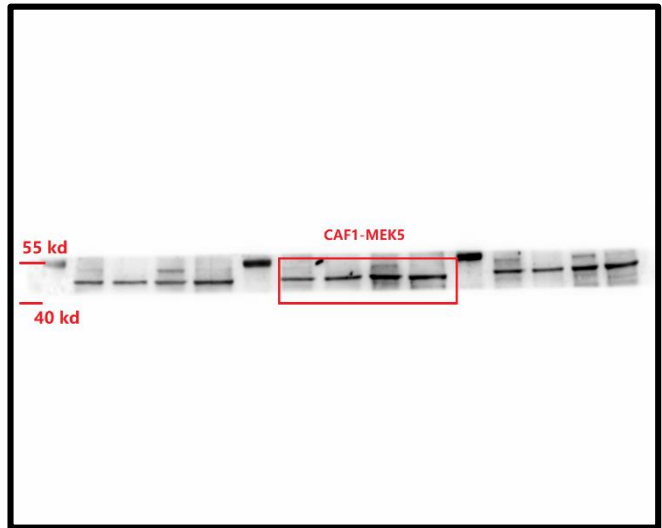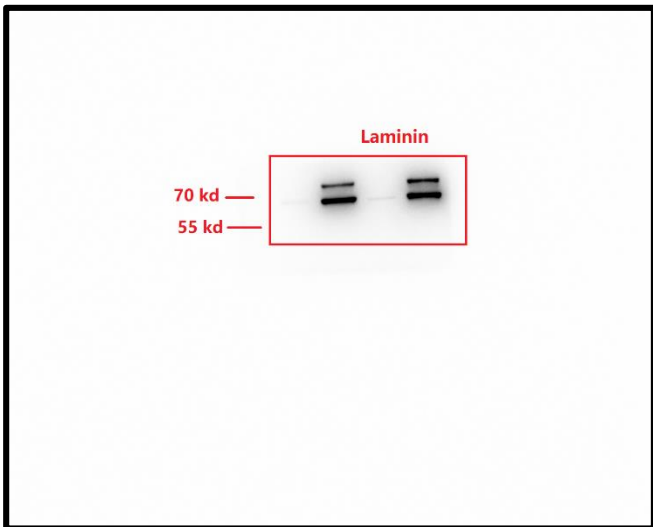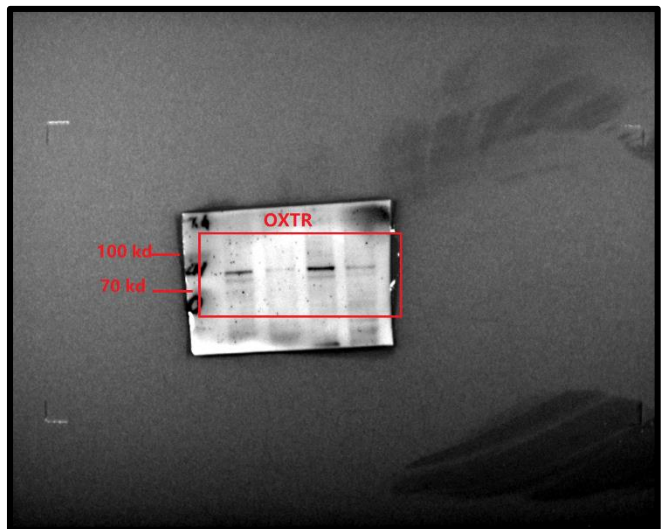

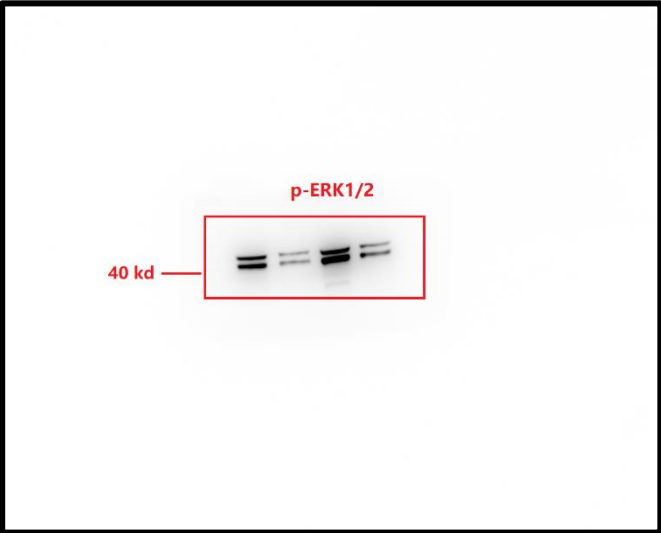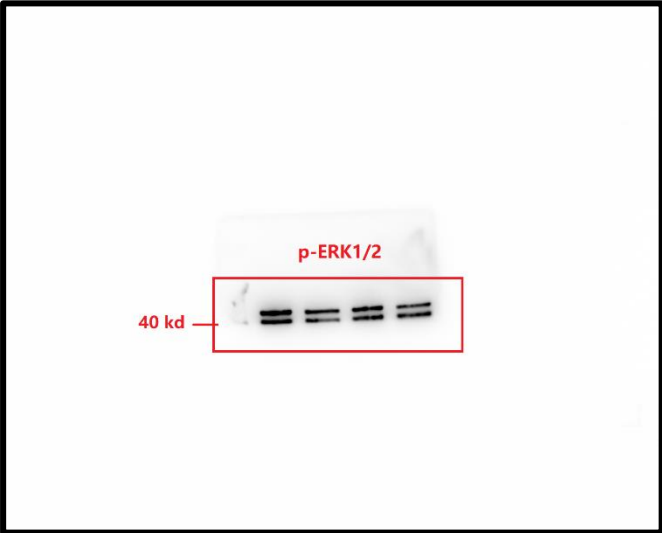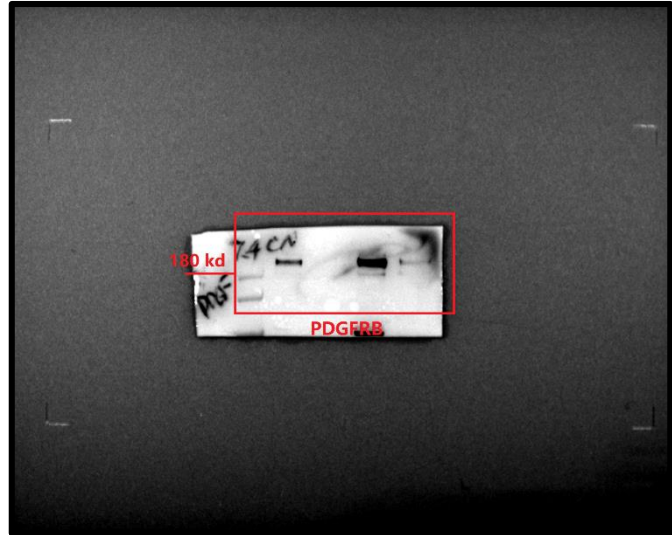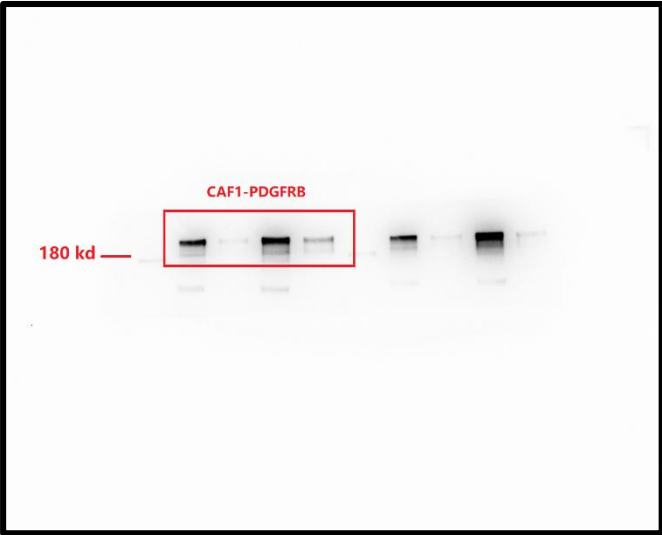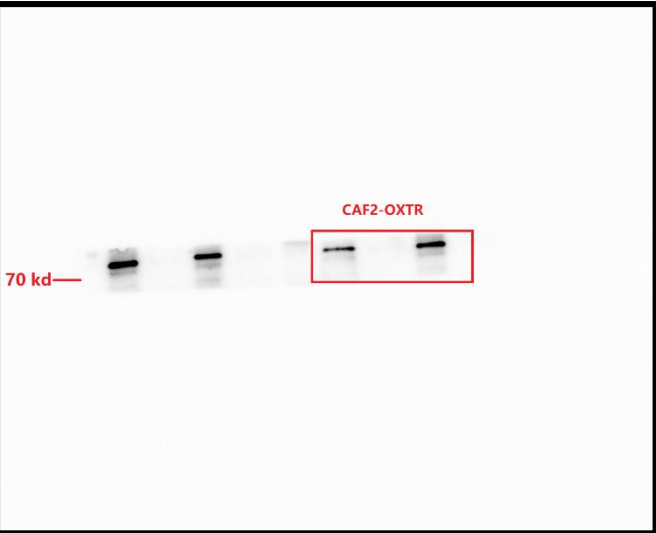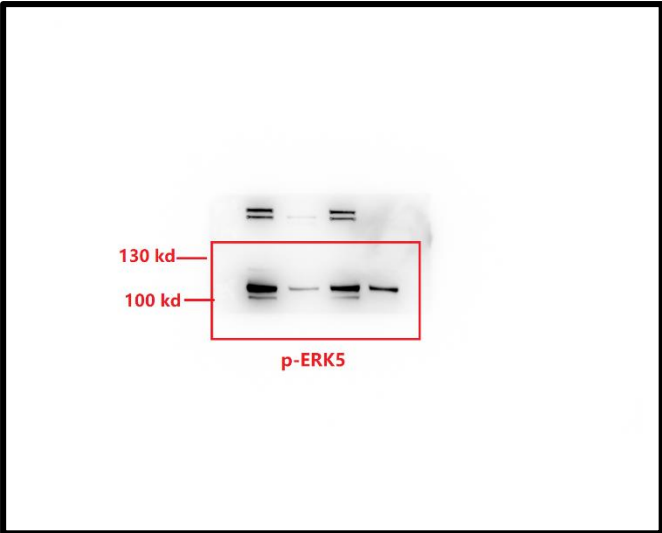

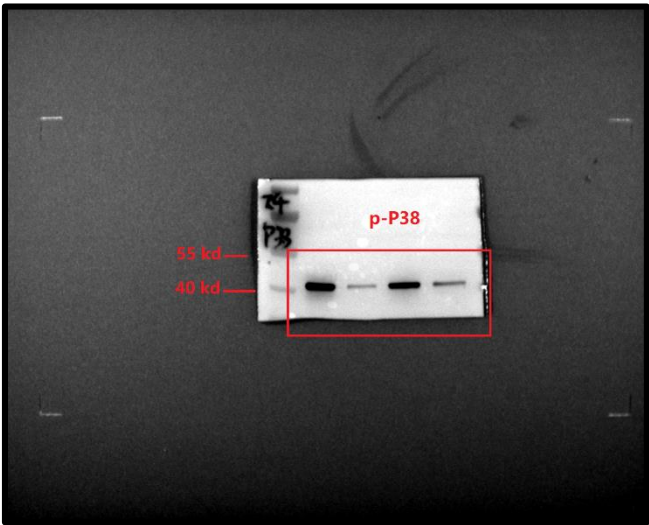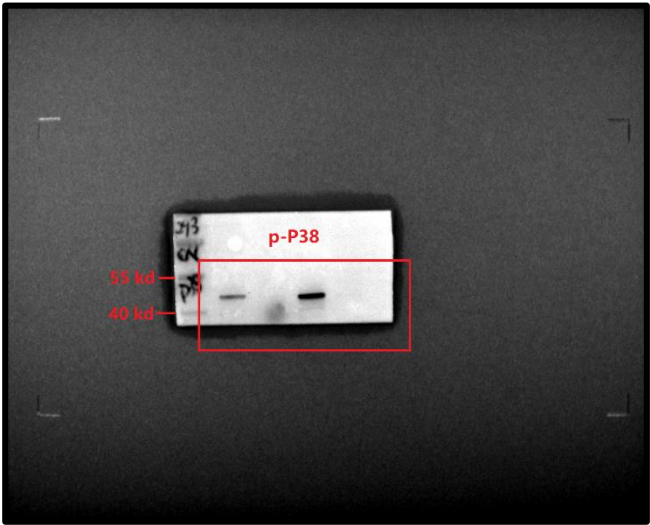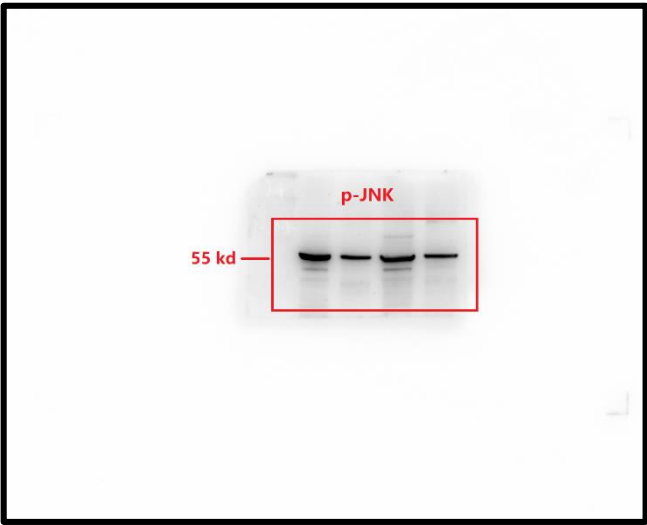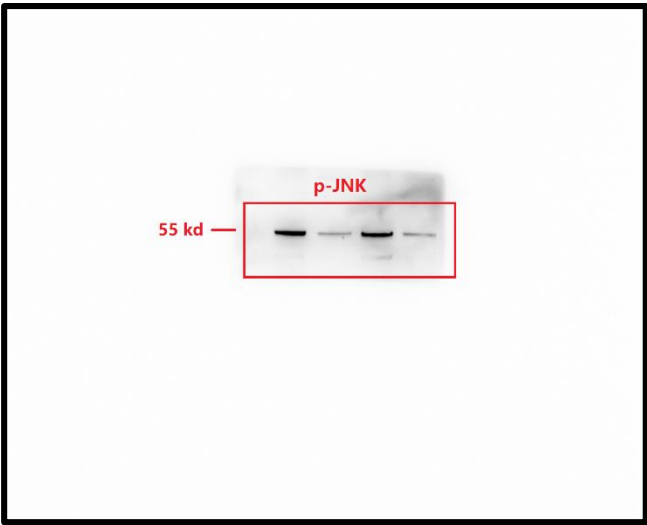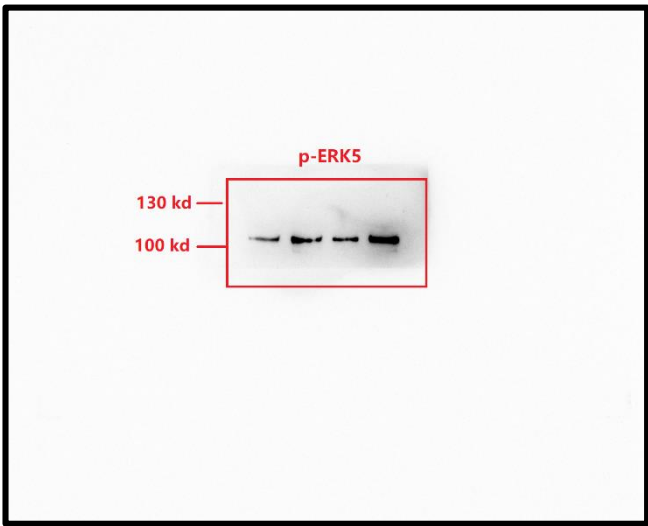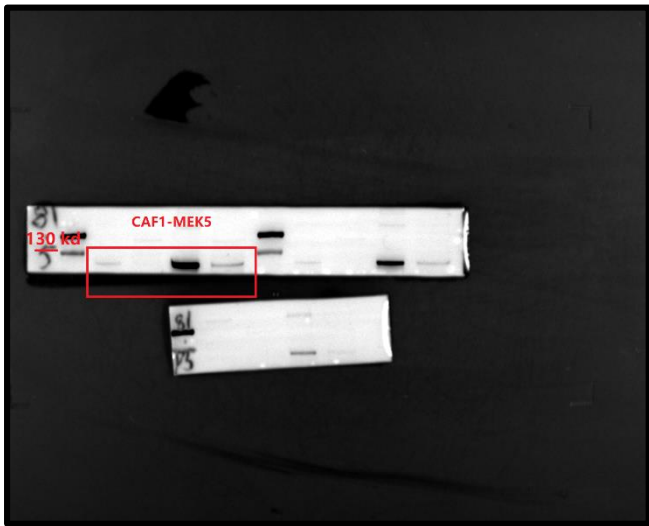

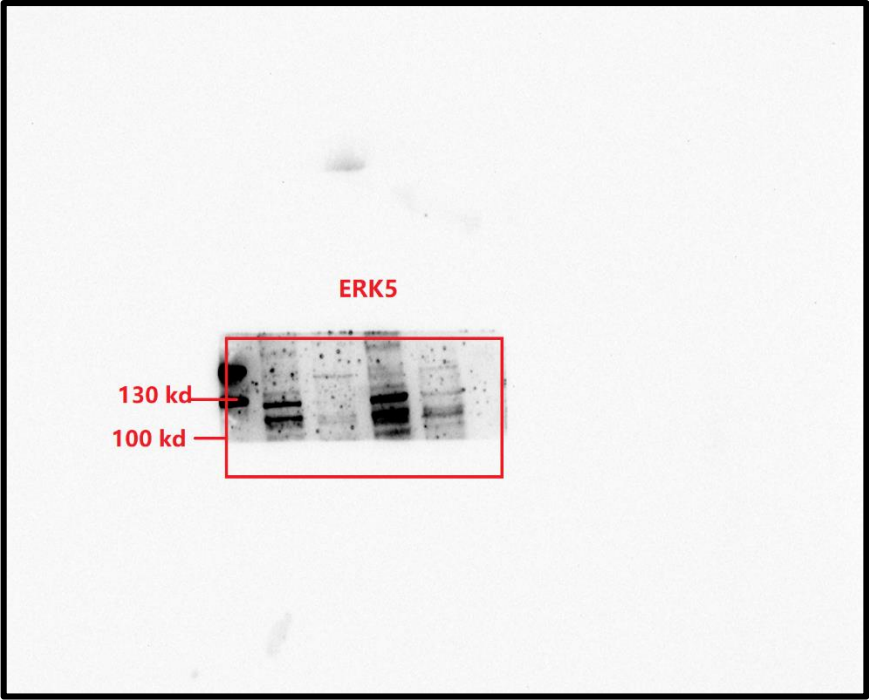

Supplement: Supplementary file 4 — Source Data [file 41467_2022_32787_MOESM4_ESM.zip › Source Data/Figure 6/Figure 6f.pdf]

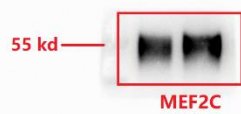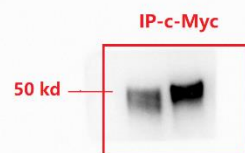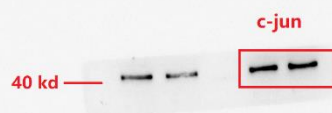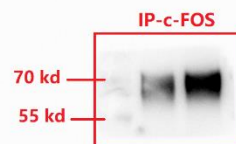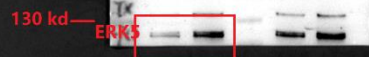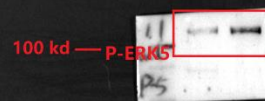

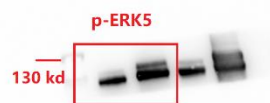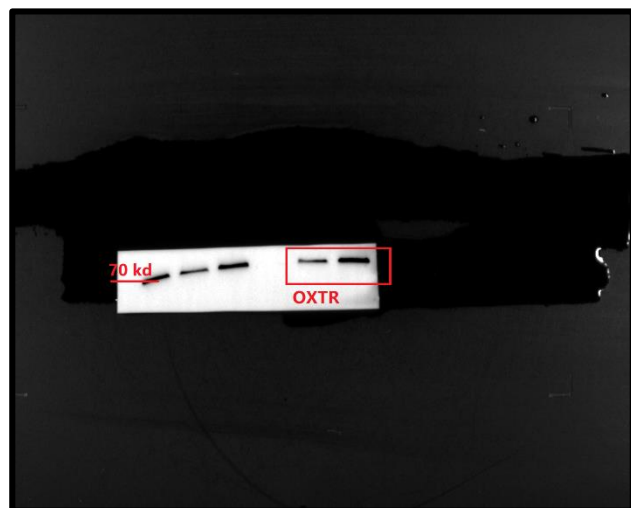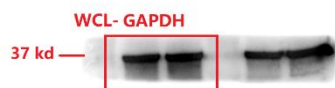

Supplement: Supplementary file 4 — Source Data [file 41467_2022_32787_MOESM4_ESM.zip › Source Data/Figure 6/Figure 6h.pdf]

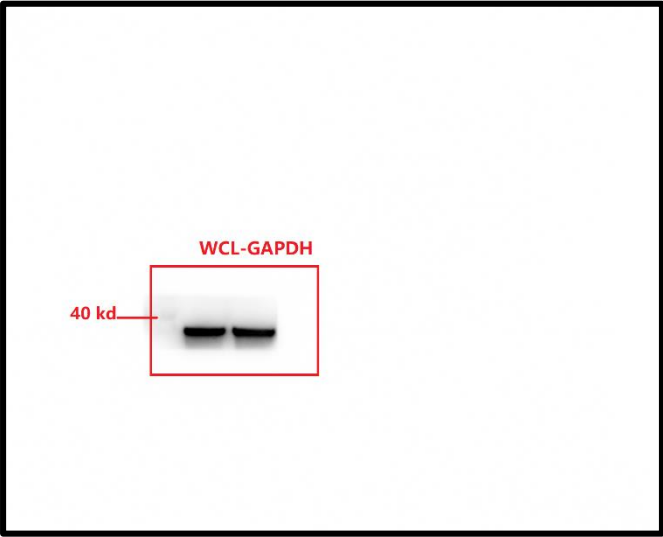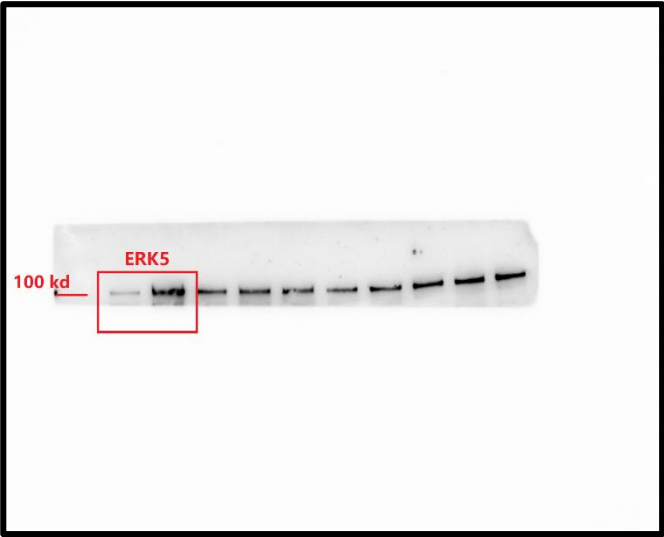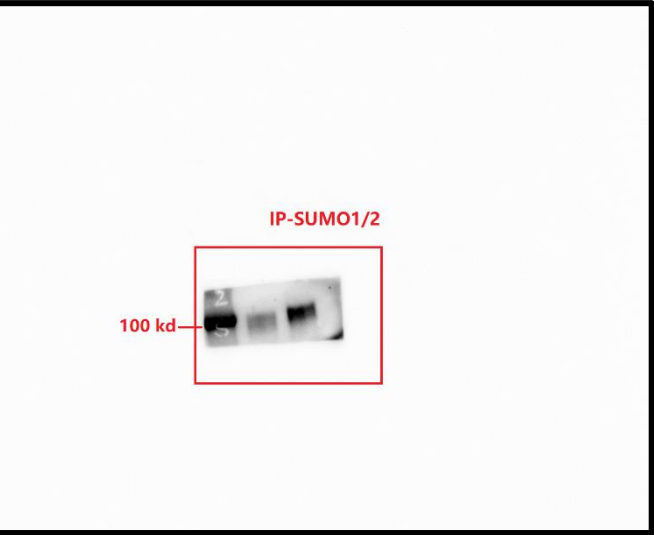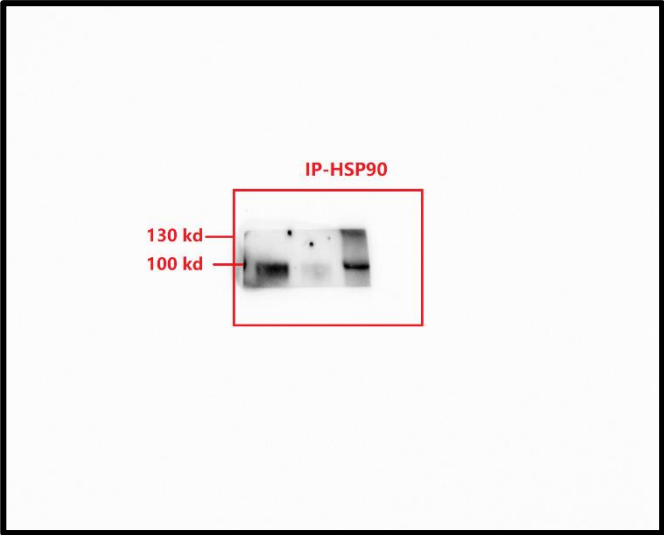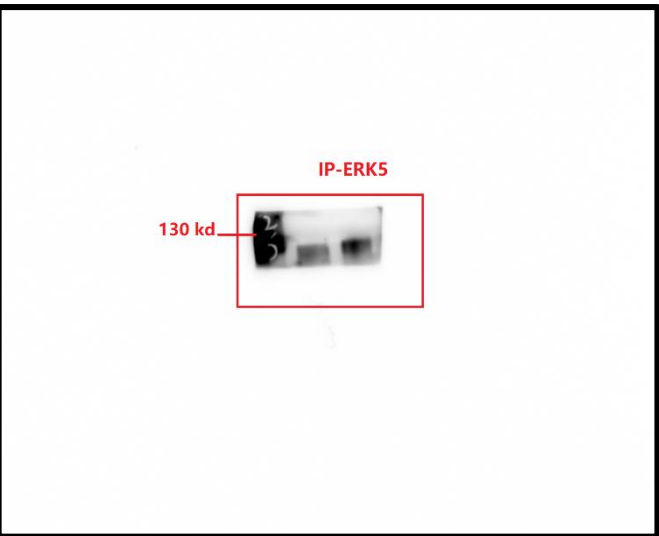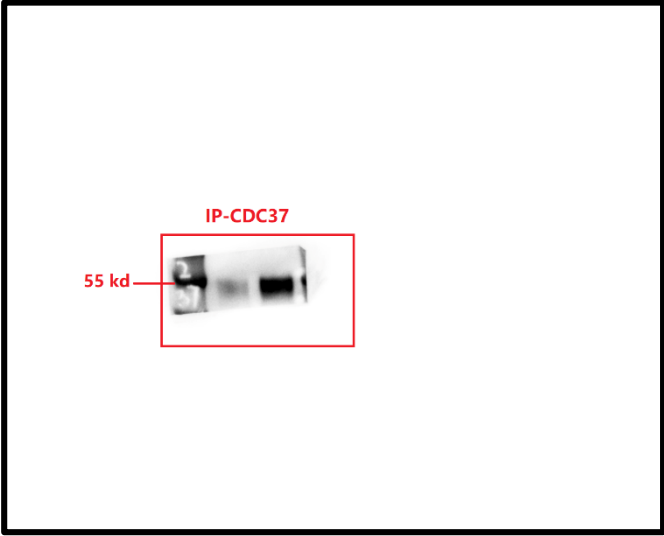

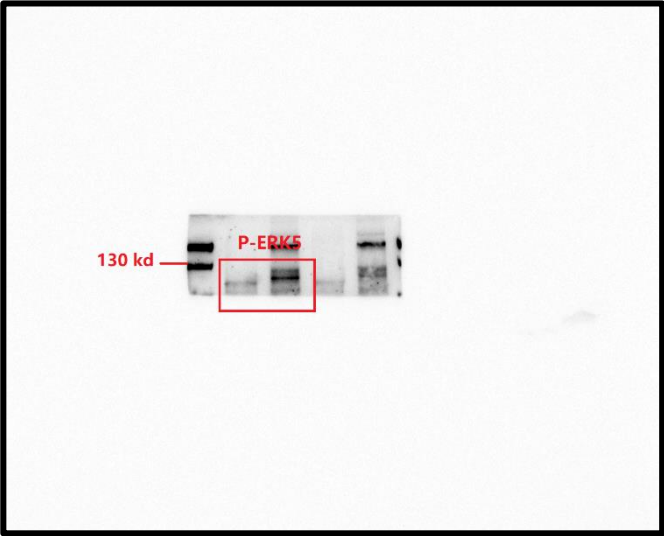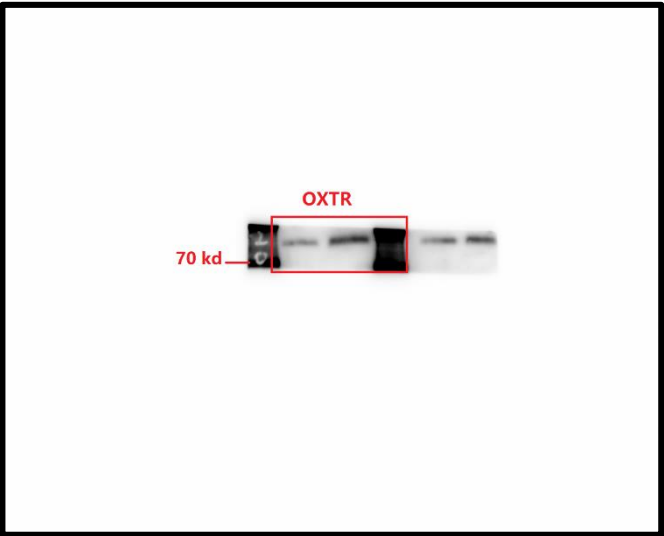

Supplement: Supplementary file 4 — Source Data [file 41467_2022_32787_MOESM4_ESM.zip › Source Data/Figure 6/Figure 6m.pdf]

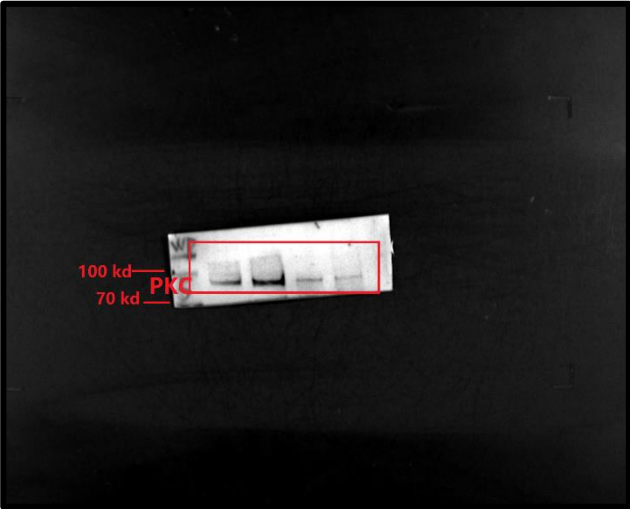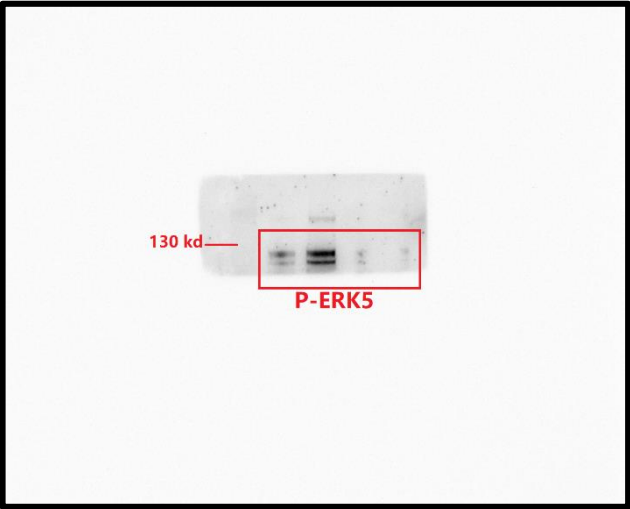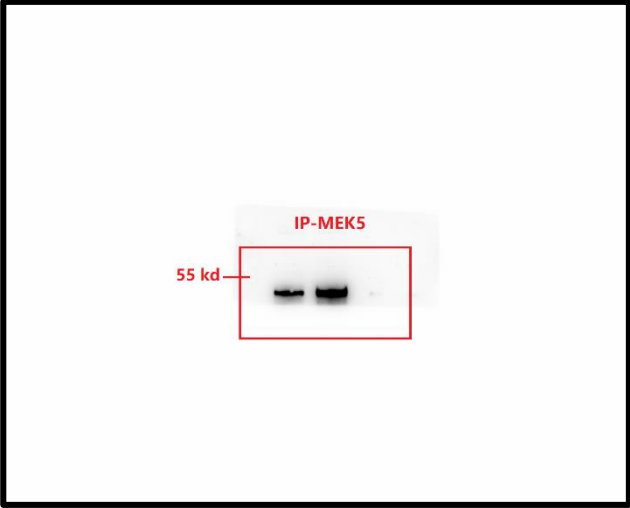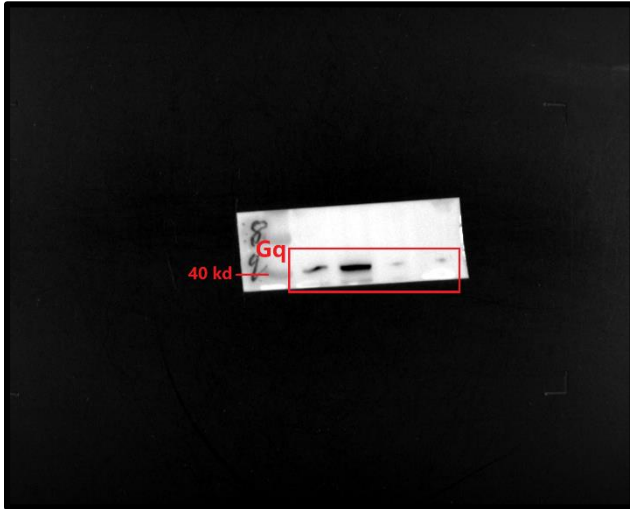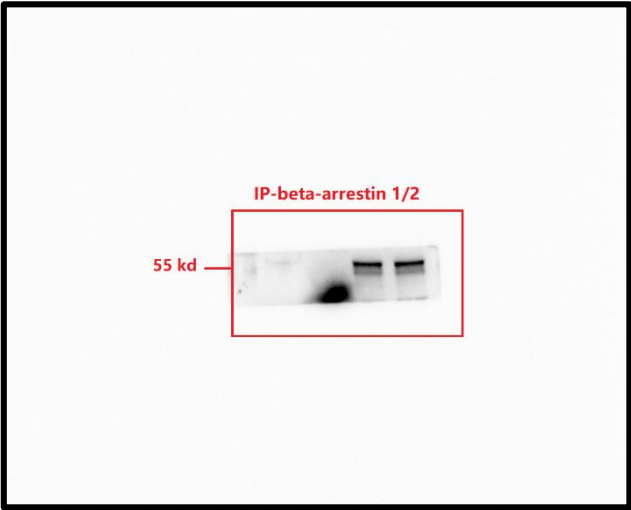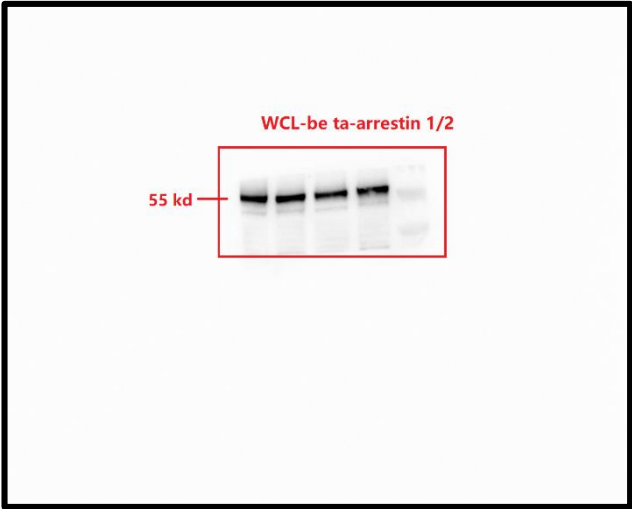

100 kd  
70 kd

OXTR

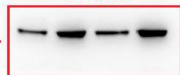

55 kd

MEK5

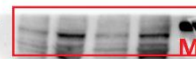

WCL-Gq

40 kd

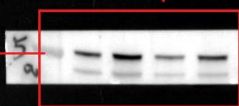

WCL-GAPDH

40 kd

35 kd

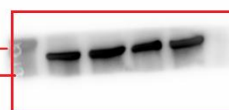

WCL-ERK5

130 kd

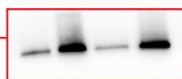

P-ERK5

100 kd

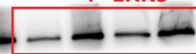

Supplement: Supplementary file 4 — Source Data [file 41467_2022_32787_MOESM4_ESM.zip › Source Data/Figure 6/Figure 6o.pdf]

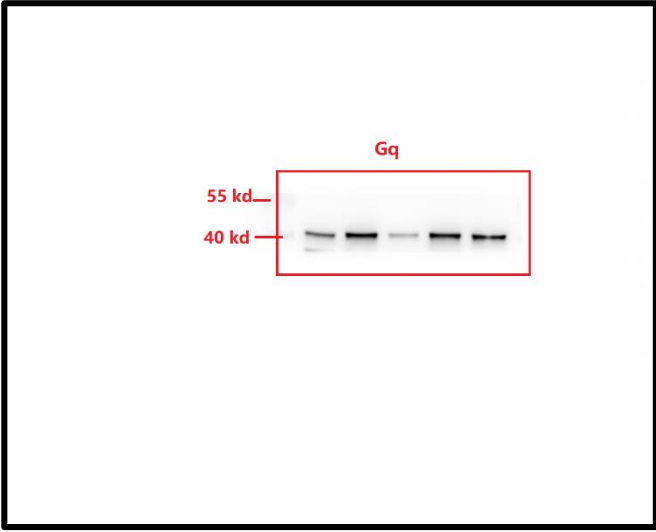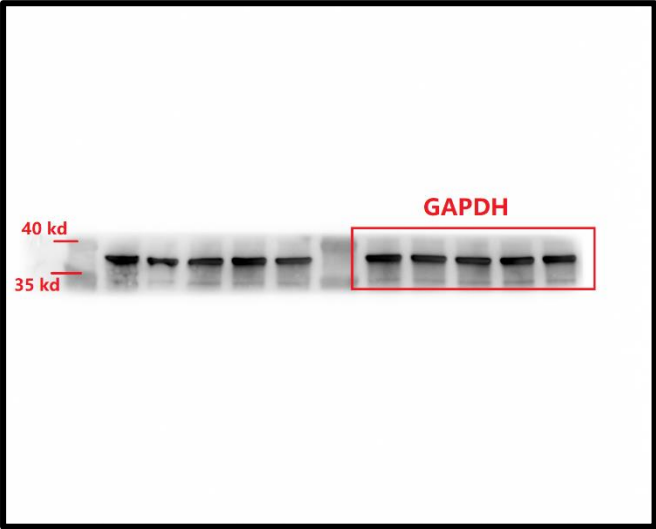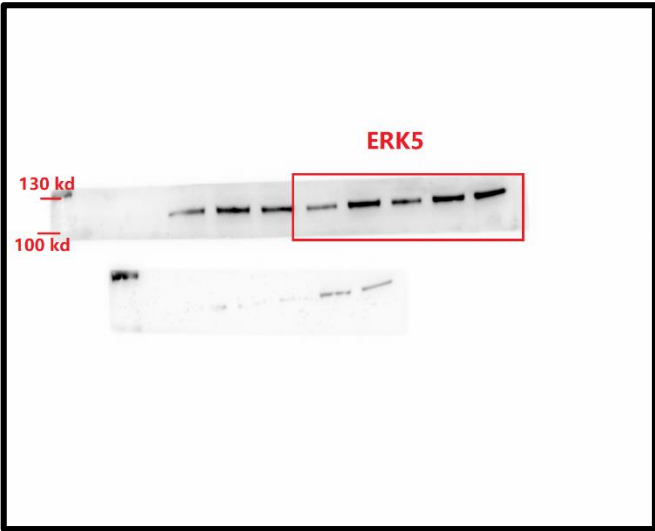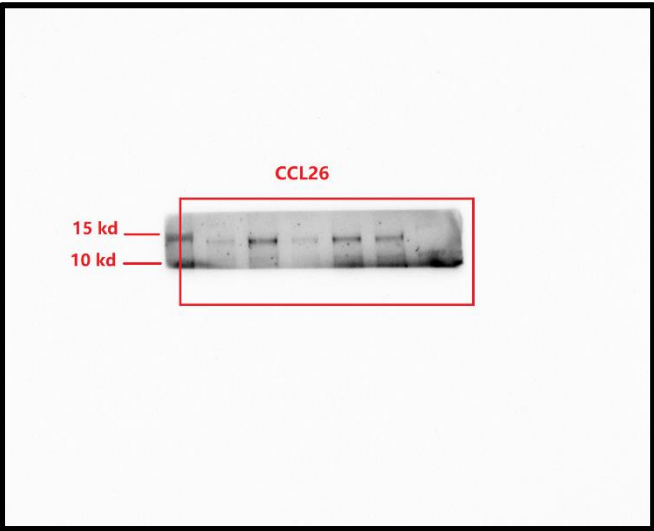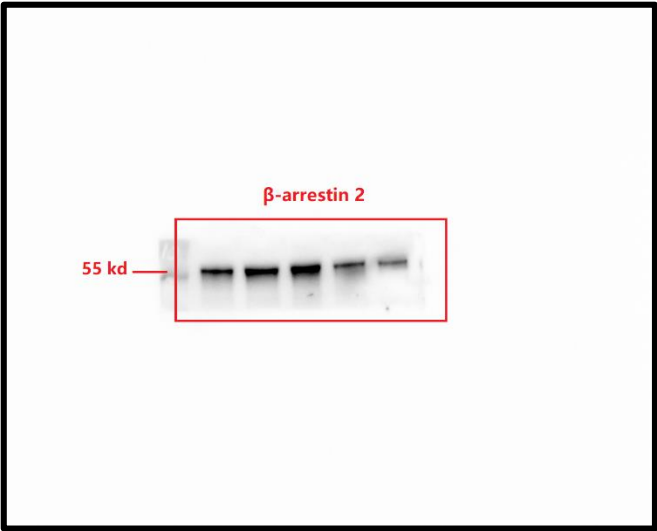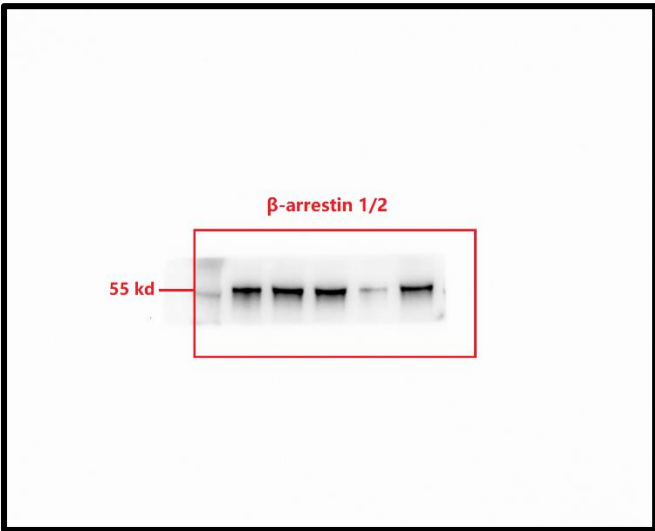

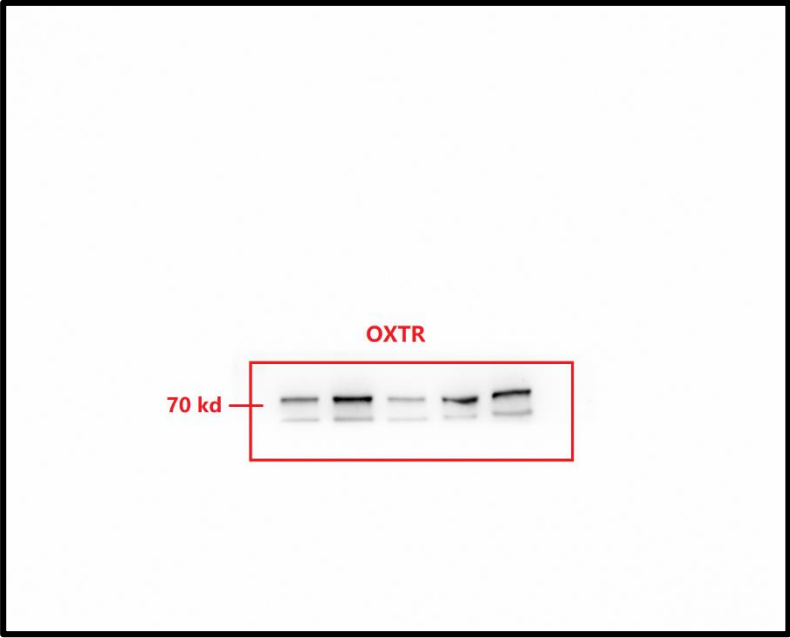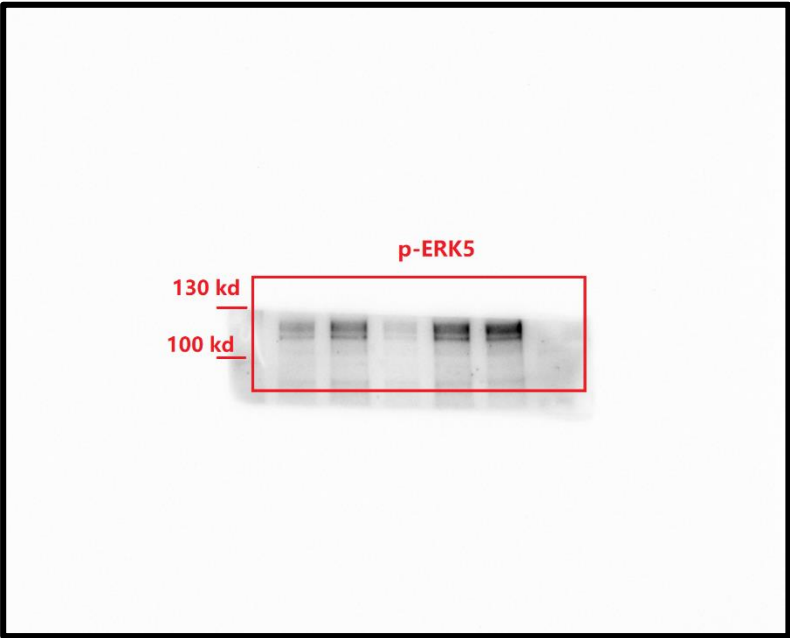

Supplement: Supplementary file 4 — Source Data [file 41467_2022_32787_MOESM4_ESM.zip › Source Data/Figure 6/Figure 6q.pdf]

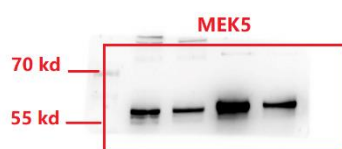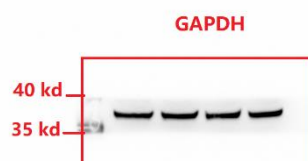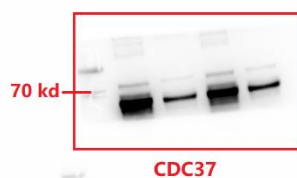

Supplement: Supplementary file 4 — Source Data [file 41467_2022_32787_MOESM4_ESM.zip › Source Data/Figure 6/Figure 6t.pdf]

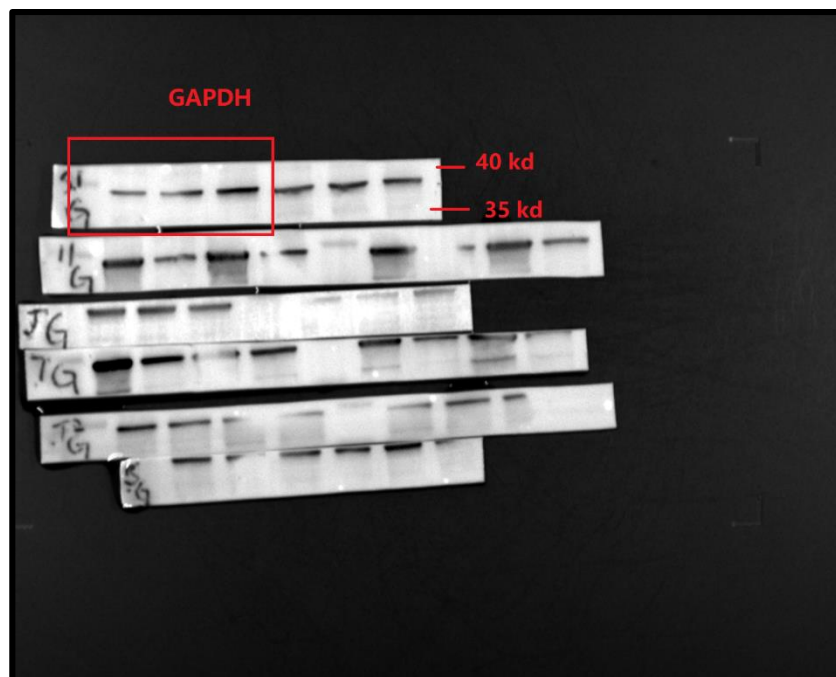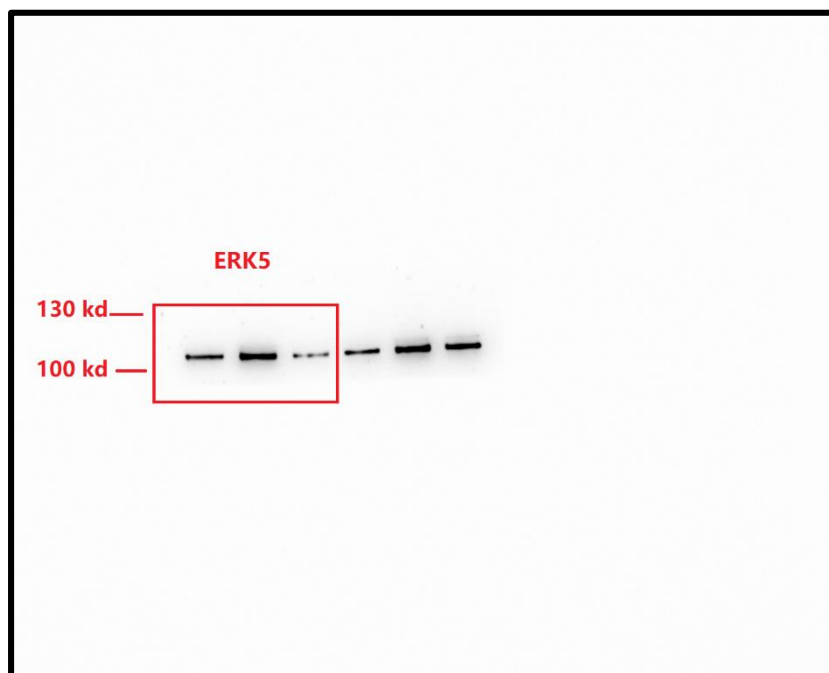

Supplement: Supplementary file 4 — Source Data [file 41467_2022_32787_MOESM4_ESM.zip › Source Data/Figure 7/Figure 7a.pdf]

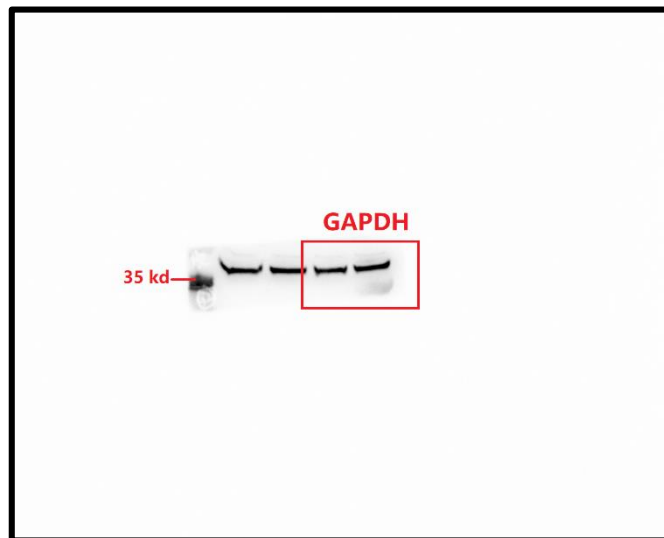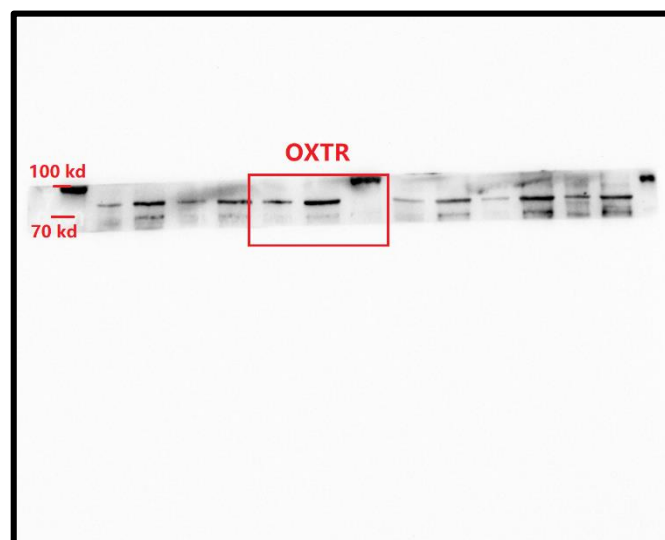

Supplement: Supplementary file 4 — Source Data [file 41467_2022_32787_MOESM4_ESM.zip › Source Data/Supplementary Figure 3/Sup Figure 3b.pdf]

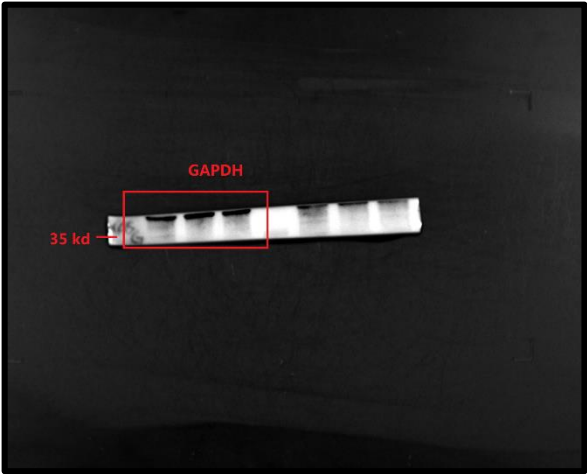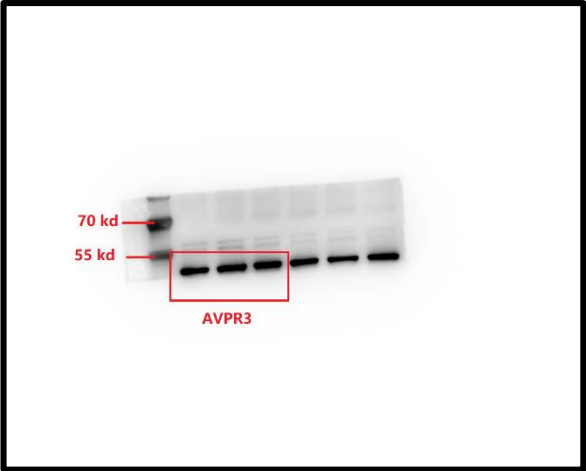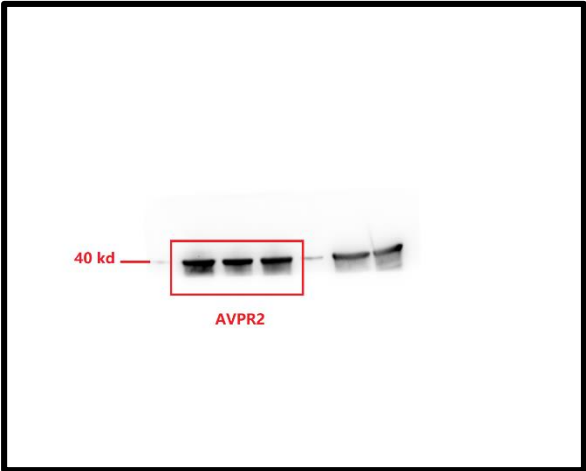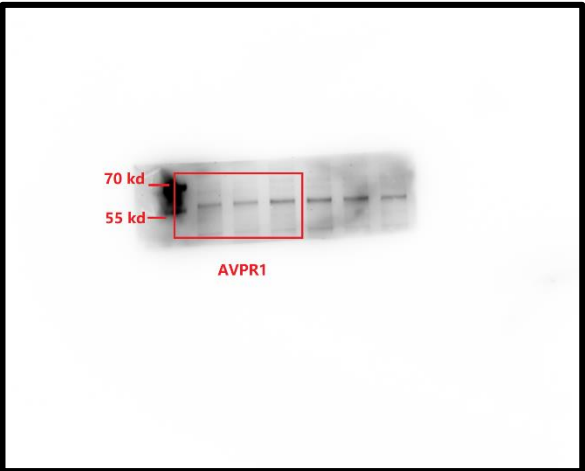

Supplement: Supplementary file 4 — Source Data [file 41467_2022_32787_MOESM4_ESM.zip › Source Data/Supplementary Figure 3/Sup Figure 3g.pdf]

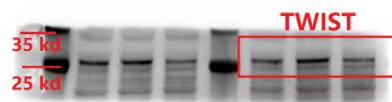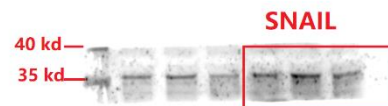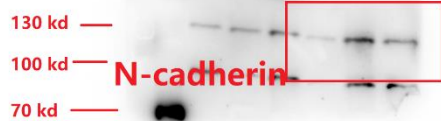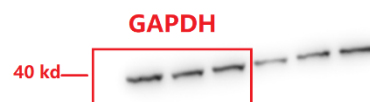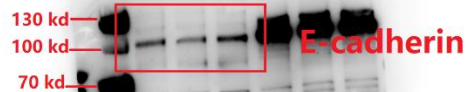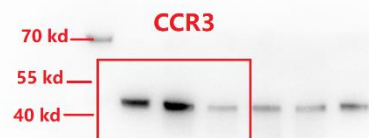

Supplement: Supplementary file 4 — Source Data [file 41467_2022_32787_MOESM4_ESM.zip › Source Data/Supplementary Figure 4/Sup Figure 4f.pdf]

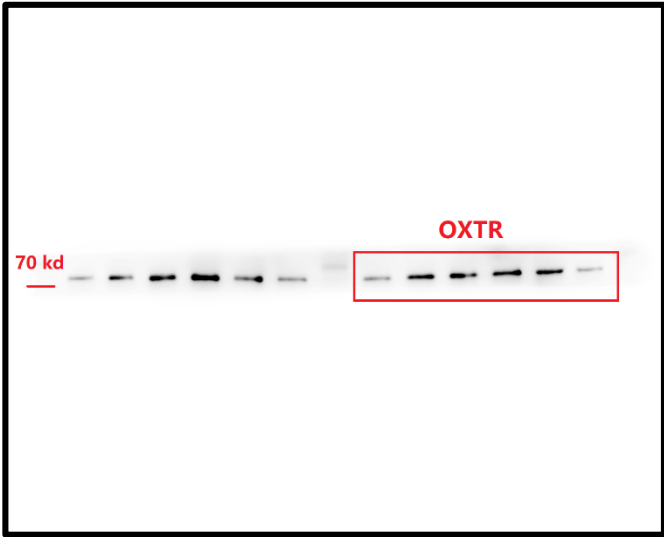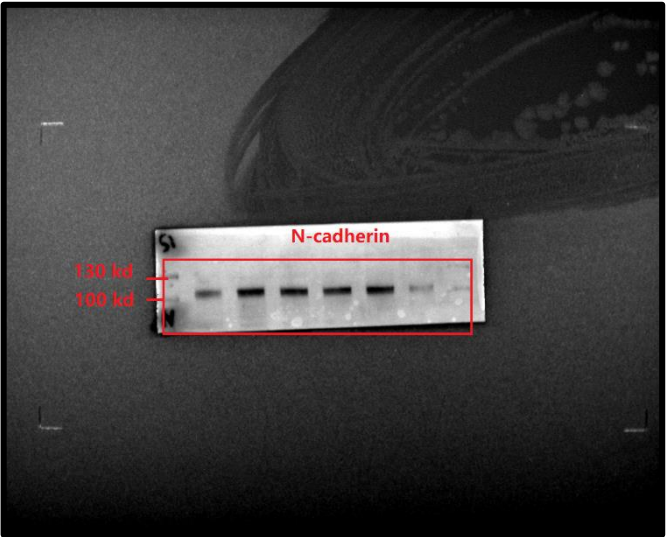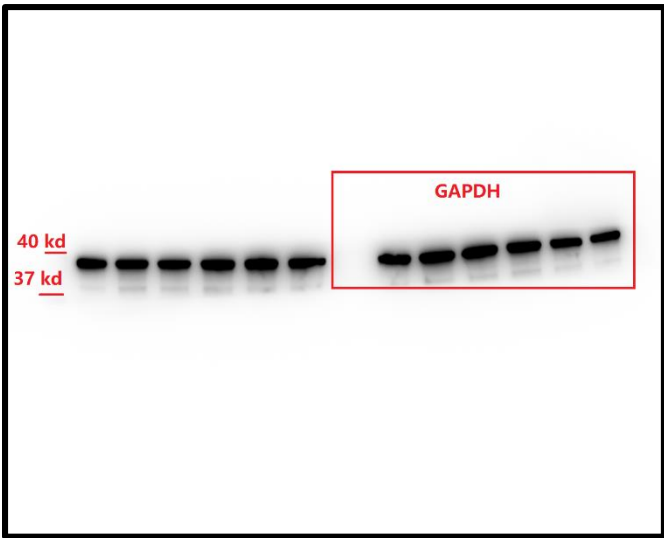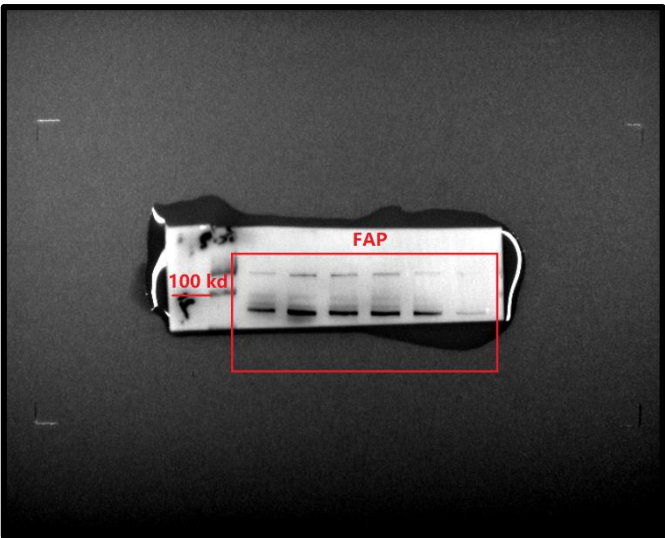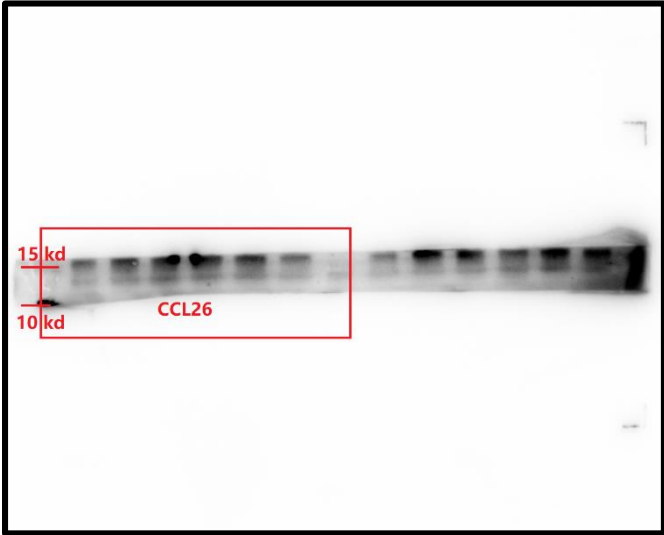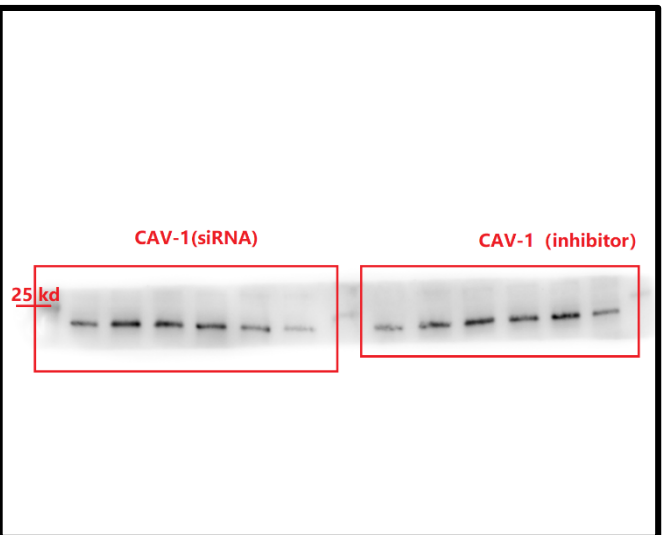

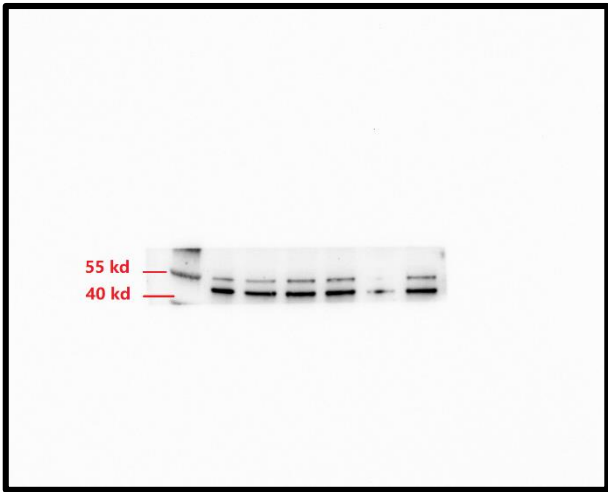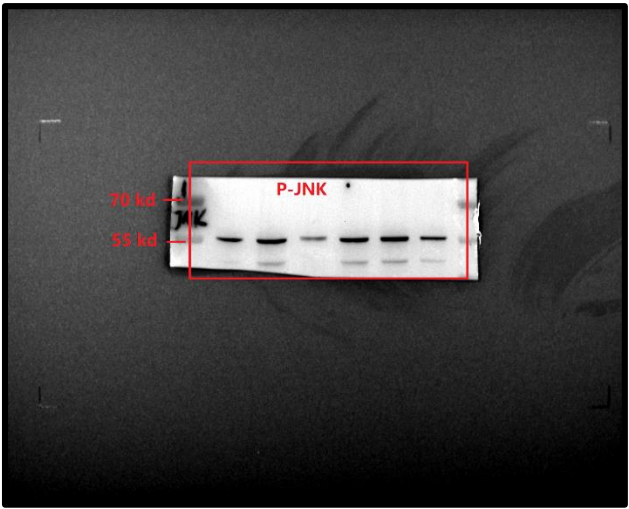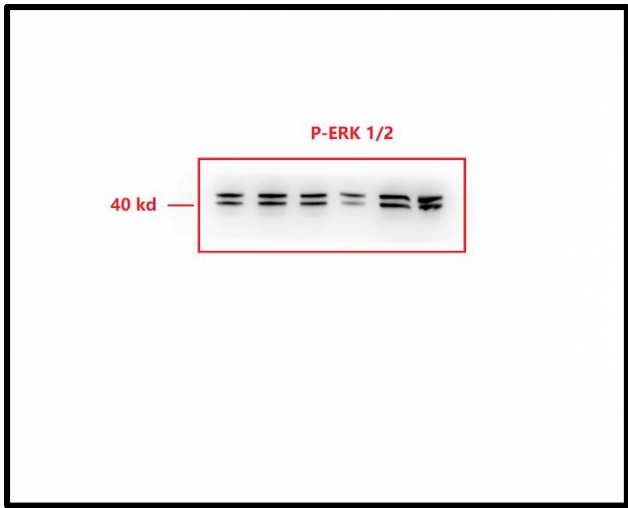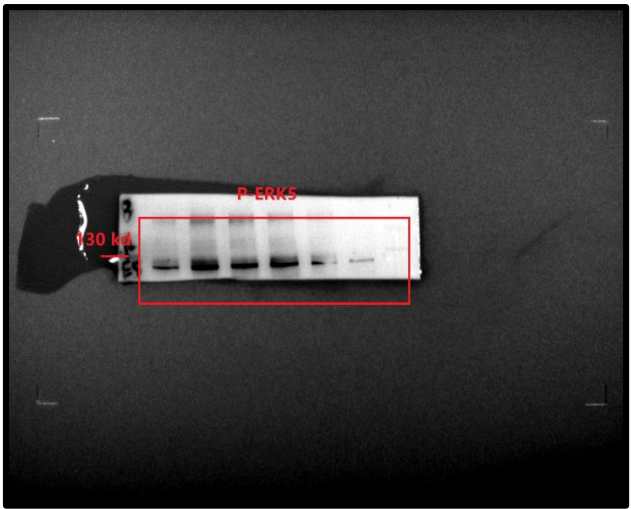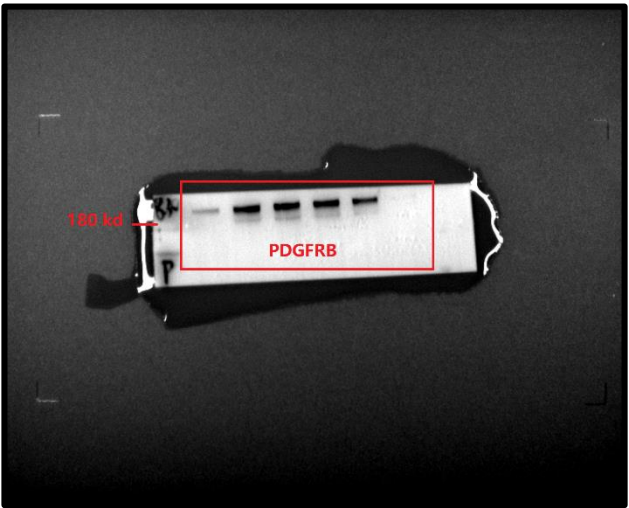

Supplement: Supplementary file 4 — Source Data [file 41467_2022_32787_MOESM4_ESM.zip › Source Data/Supplementary Figure 5/Sup Figure 5b.pdf]

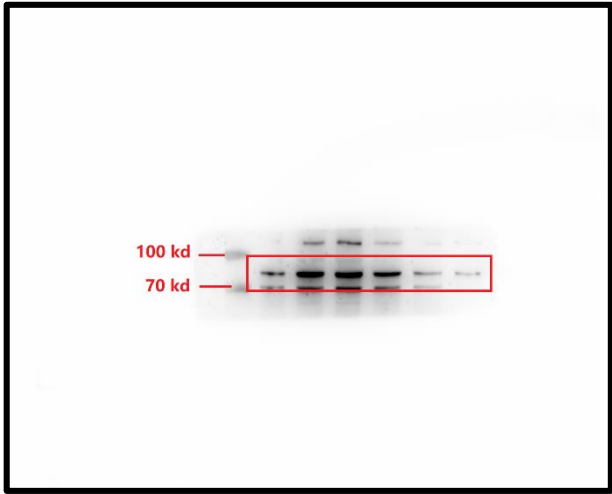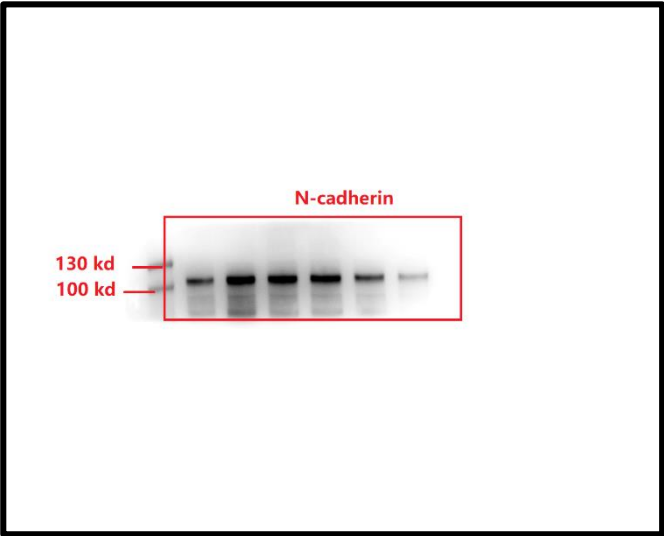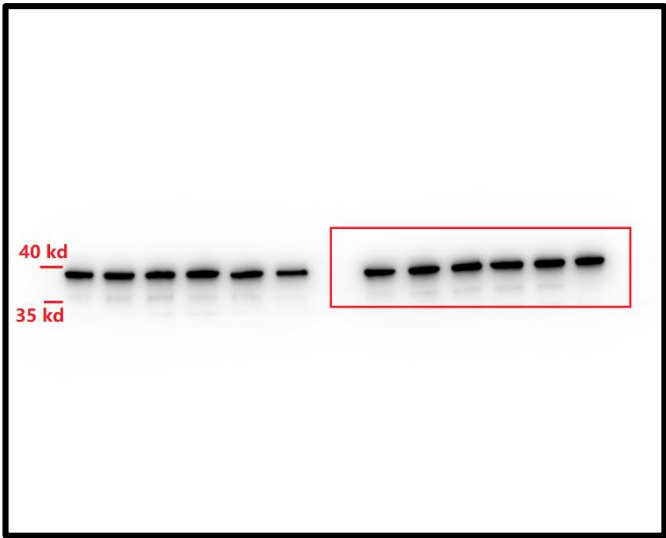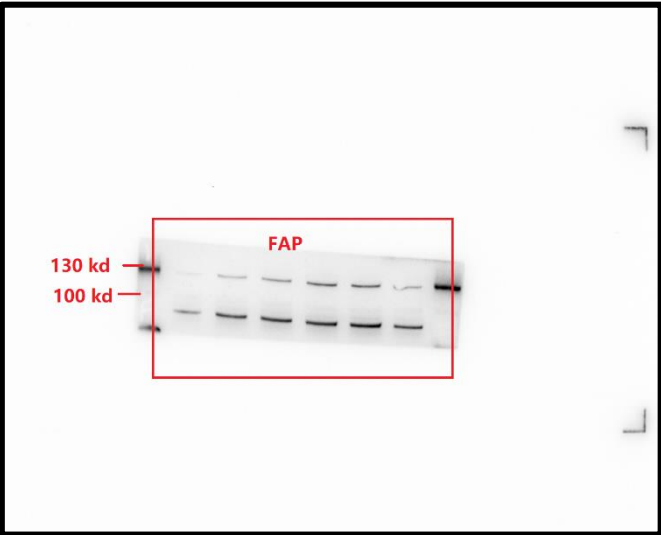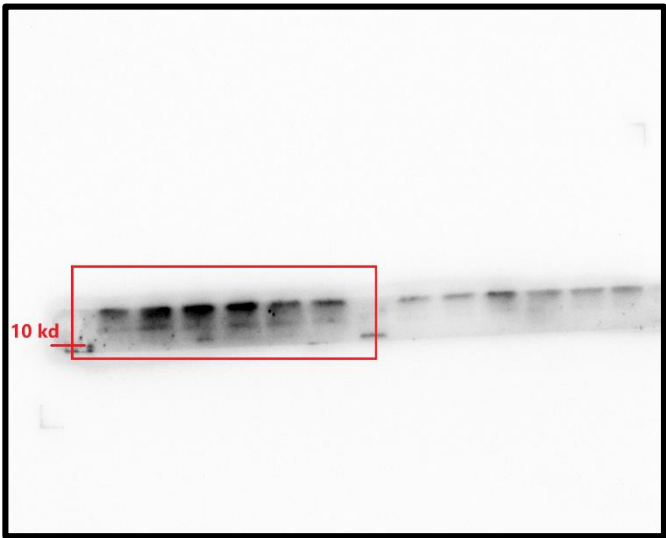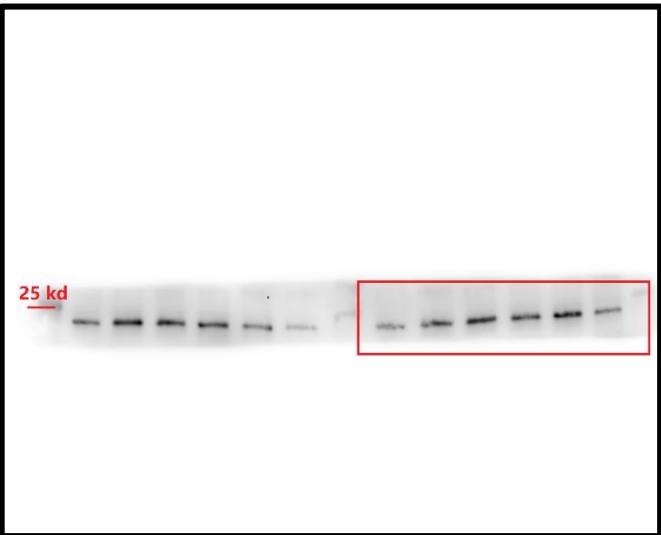

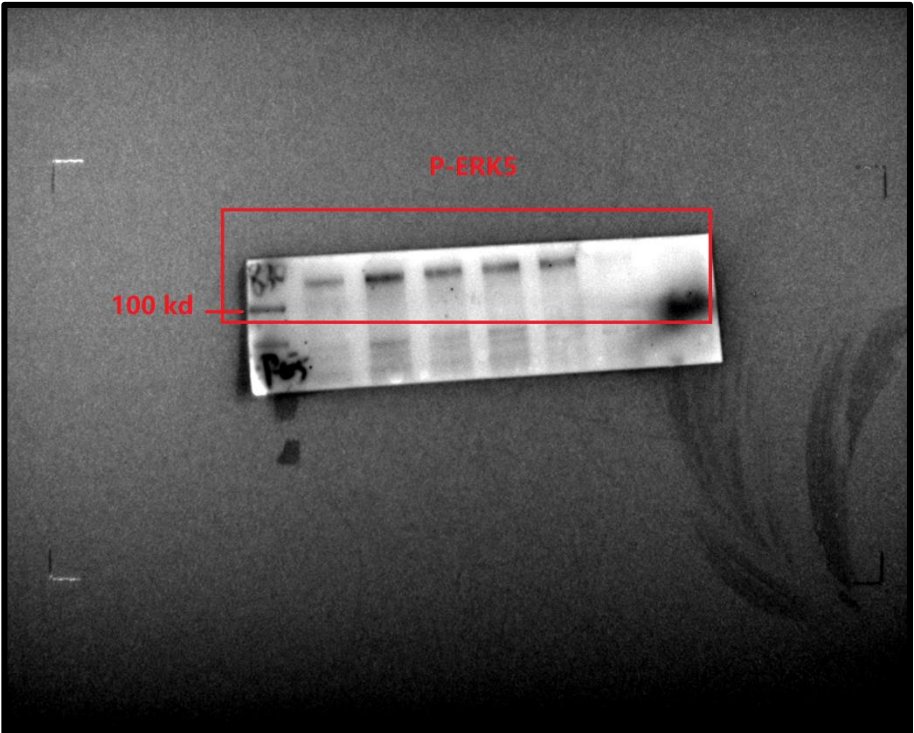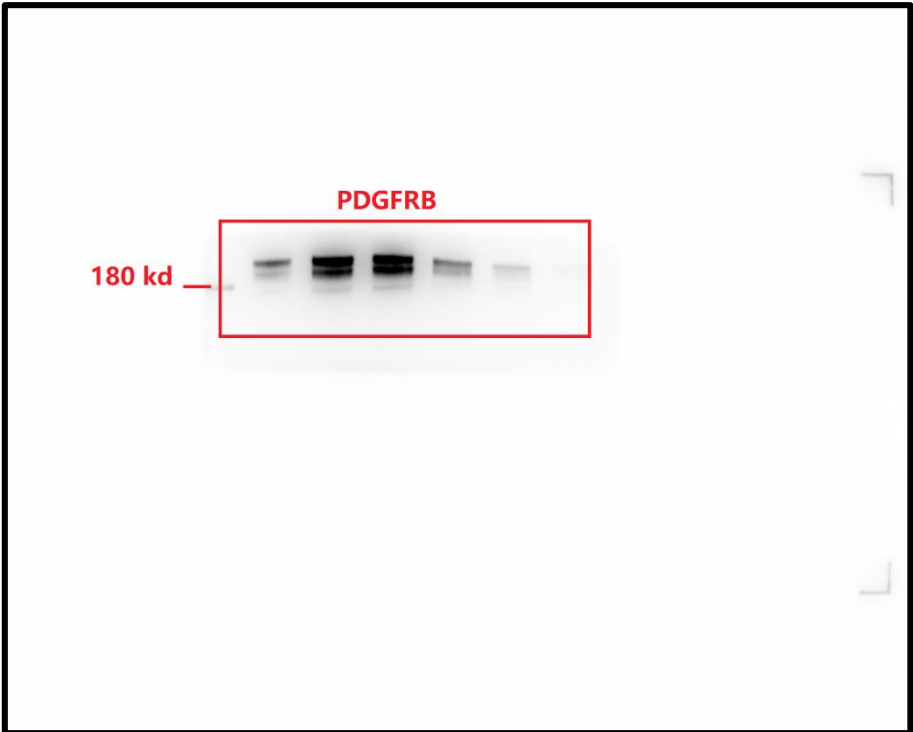

Supplement: Supplementary file 4 — Source Data [file 41467_2022_32787_MOESM4_ESM.zip › Source Data/Supplementary Figure 5/Sup Figure 5c.pdf]

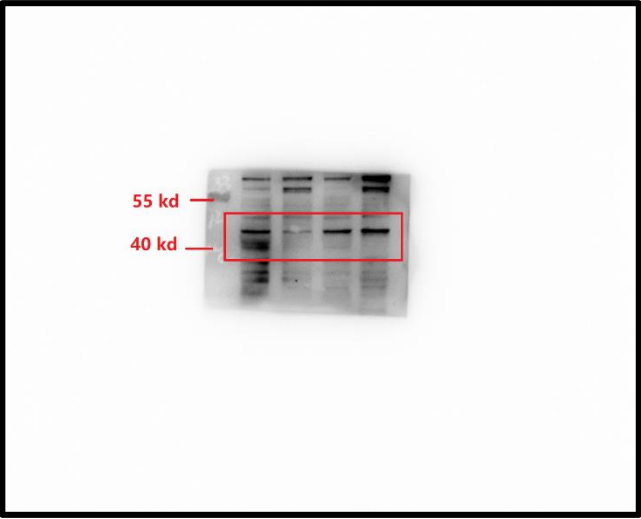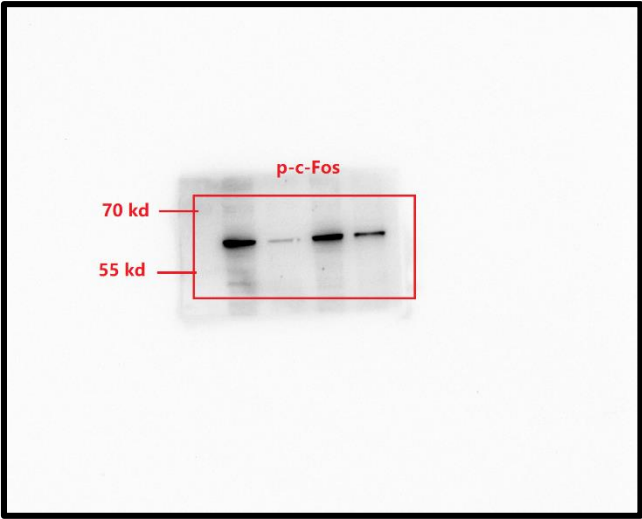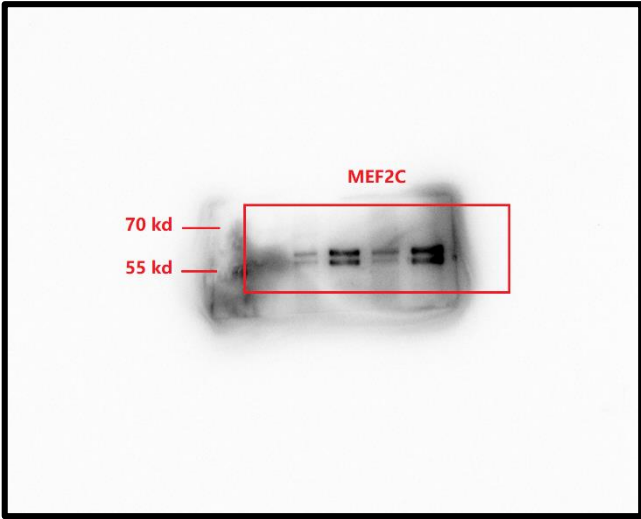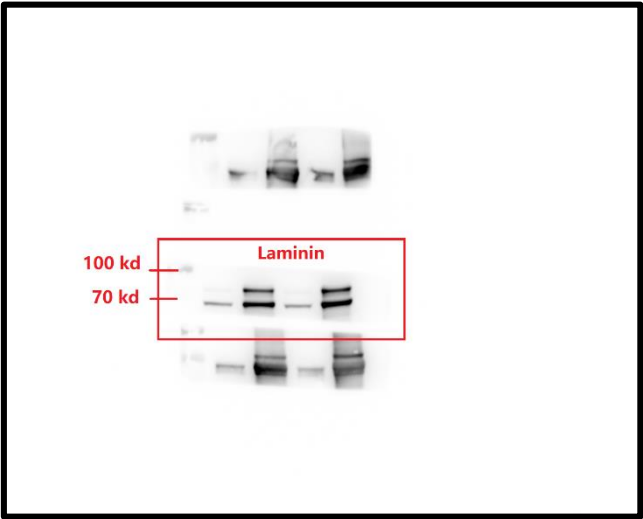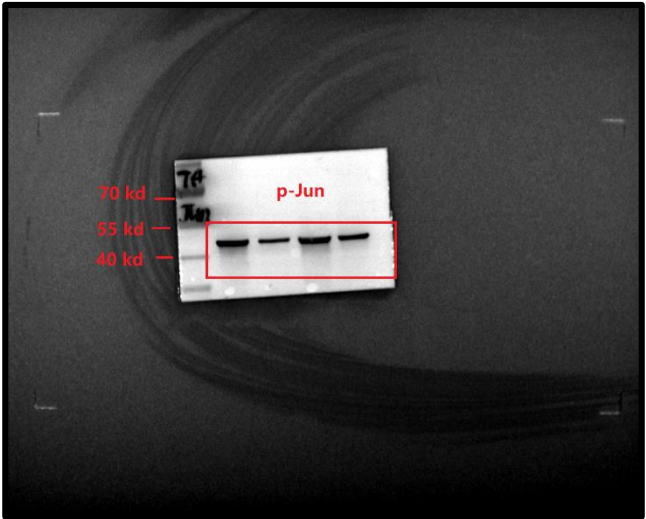

Supplement: Supplementary file 4 — Source Data [file 41467_2022_32787_MOESM4_ESM.zip › Source Data/Supplementary Figure 5/Sup Figure 5e.pdf]

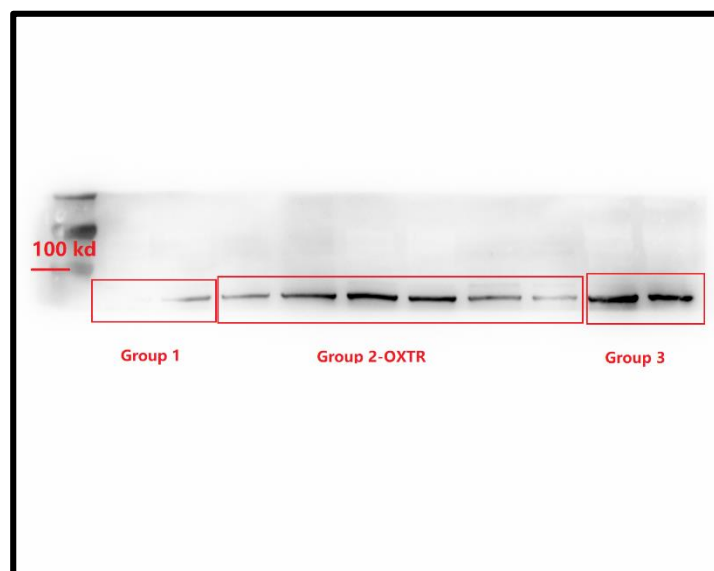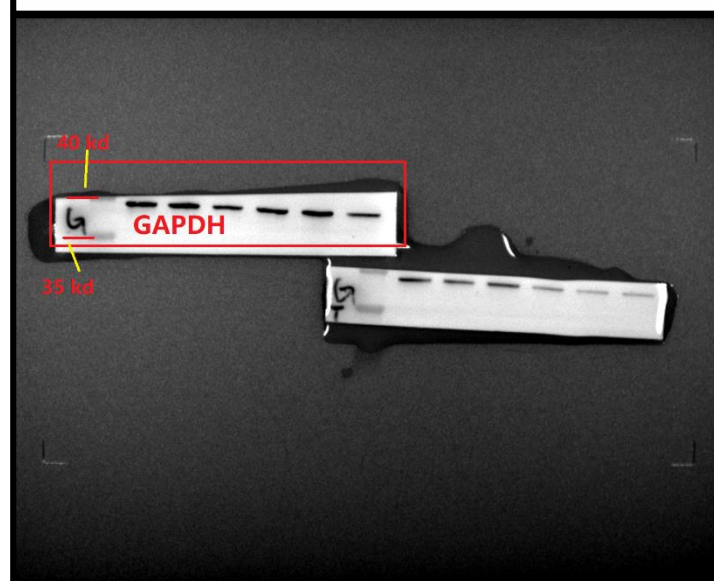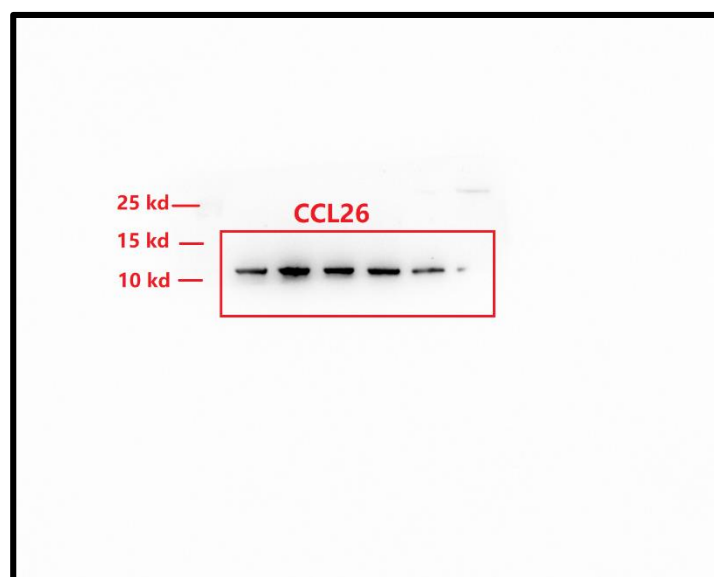

Supplement: Supplementary file 4 — Source Data [file 41467_2022_32787_MOESM4_ESM.zip › Source Data/Supplementary Figure 5/Sup Figure 5g.pdf]

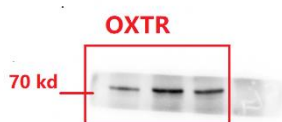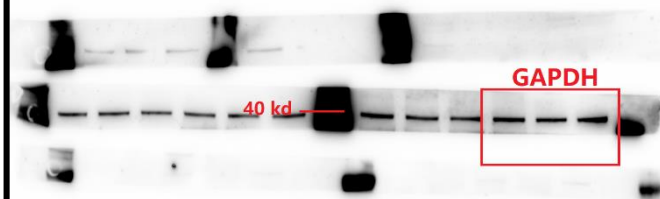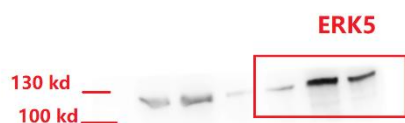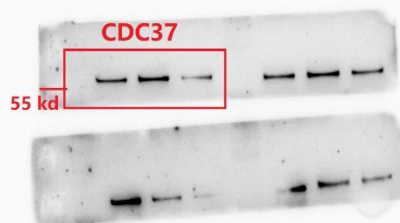

Supplement: Supplementary file 4 — Source Data [file 41467_2022_32787_MOESM4_ESM.zip › Source Data/Supplementary Figure 5/Sup Figure 5i.pdf]

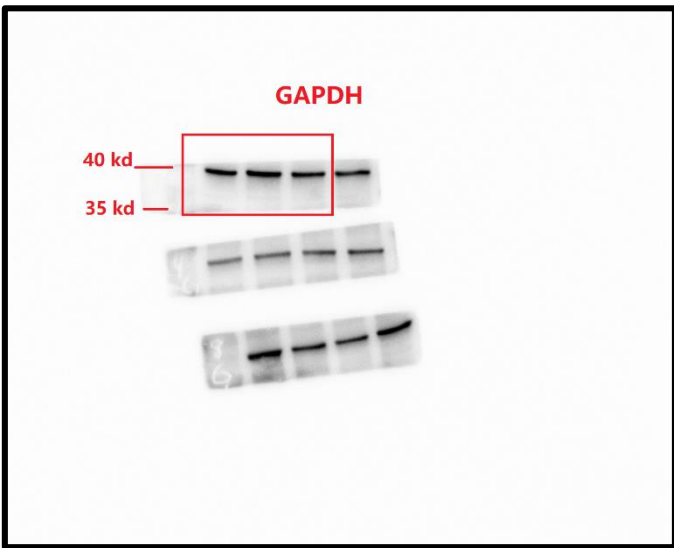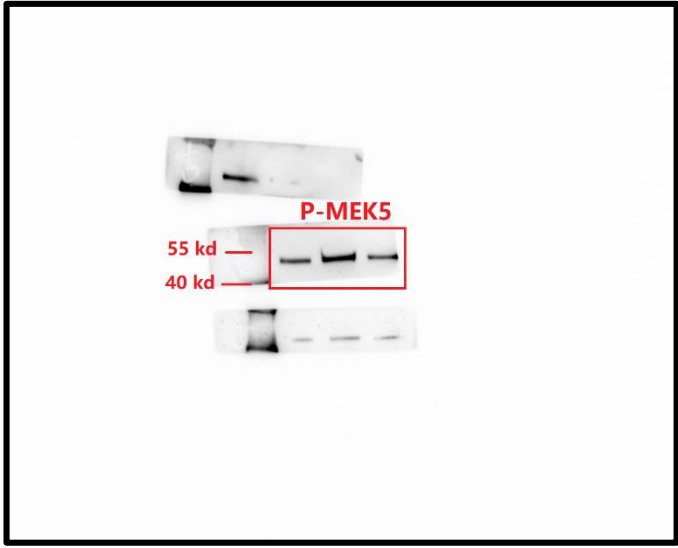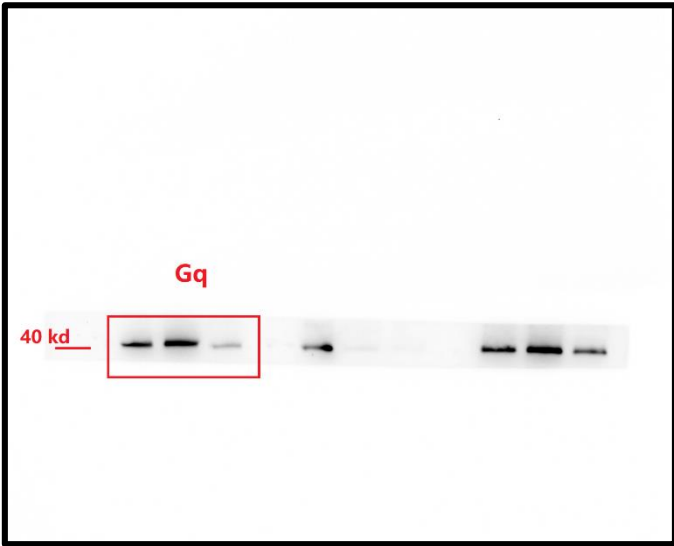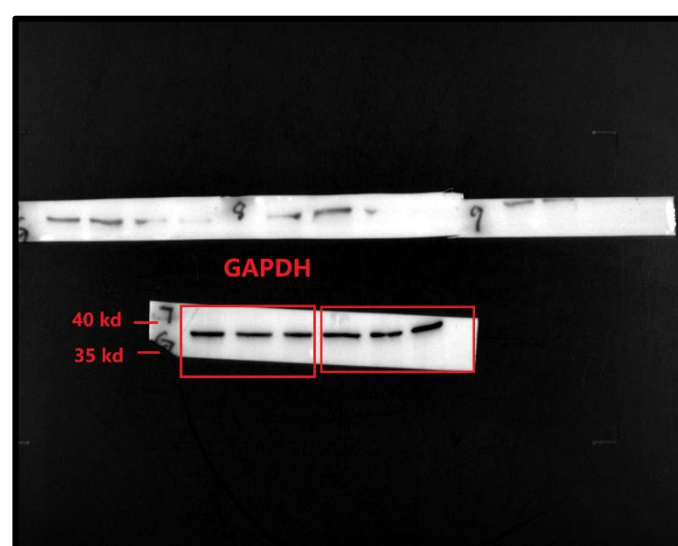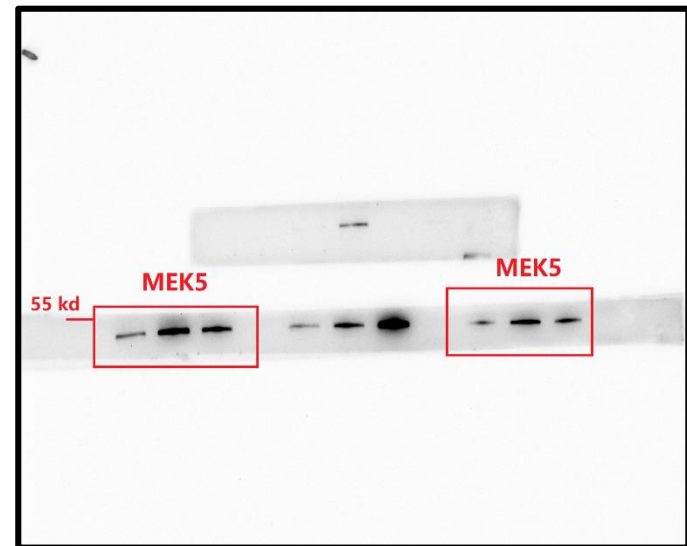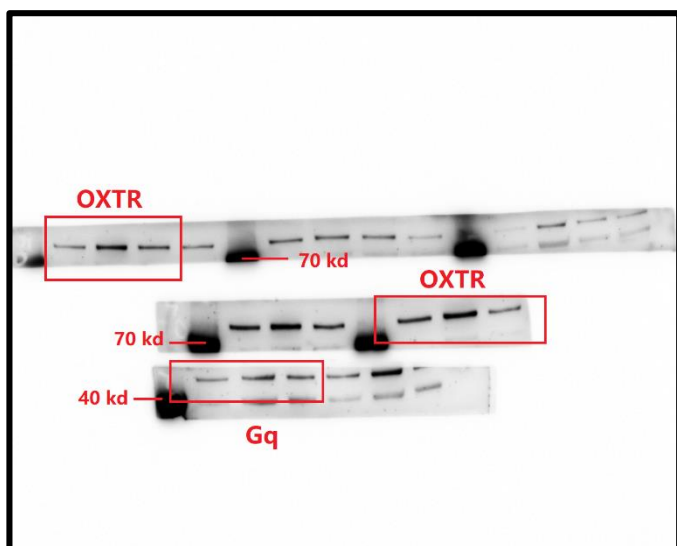

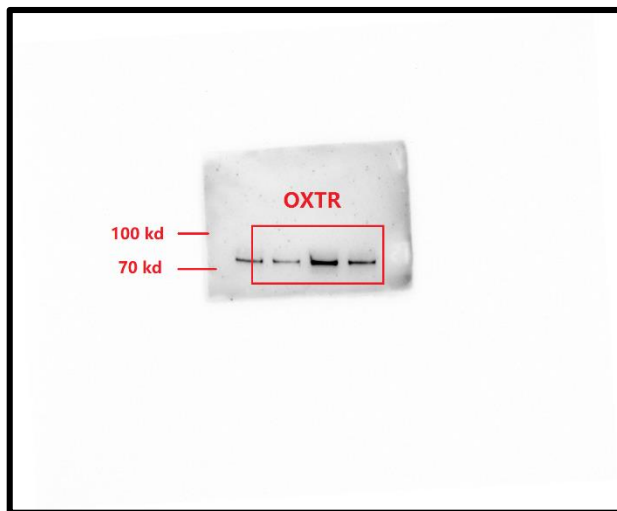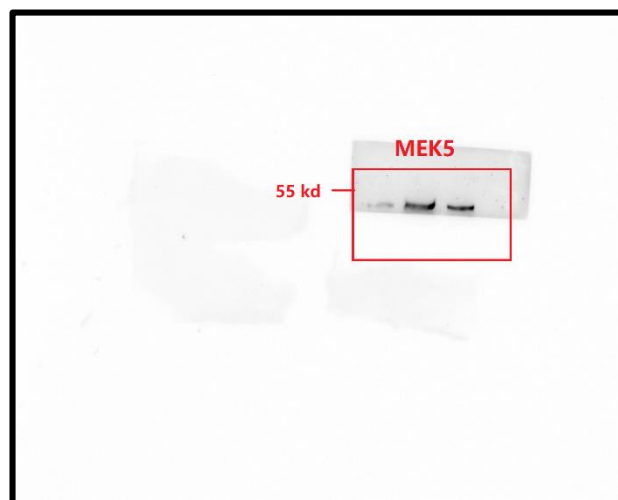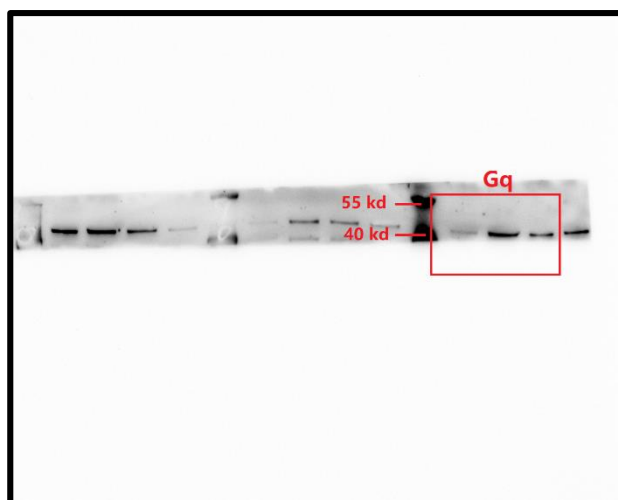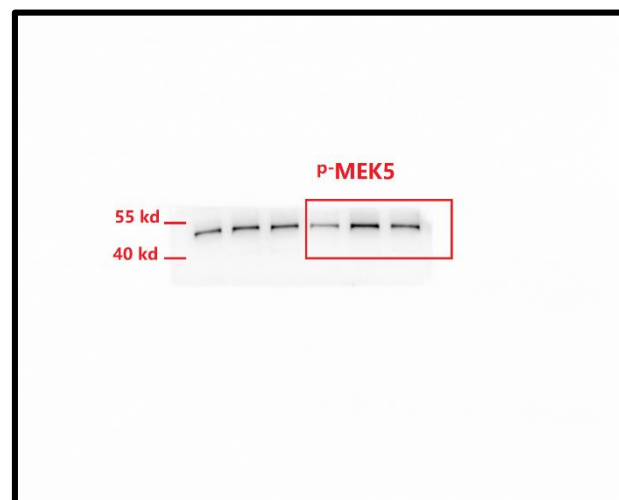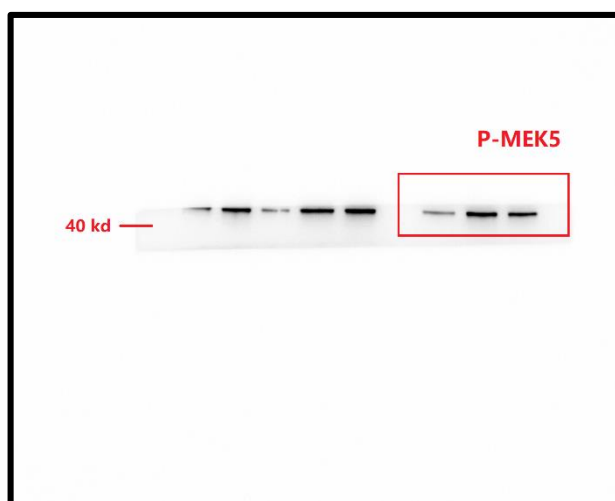

Supplement: Supplementary file 4 — Source Data [file 41467_2022_32787_MOESM4_ESM.zip › Source Data/Supplementary Figure 5/Sup Figure 5k.pdf]

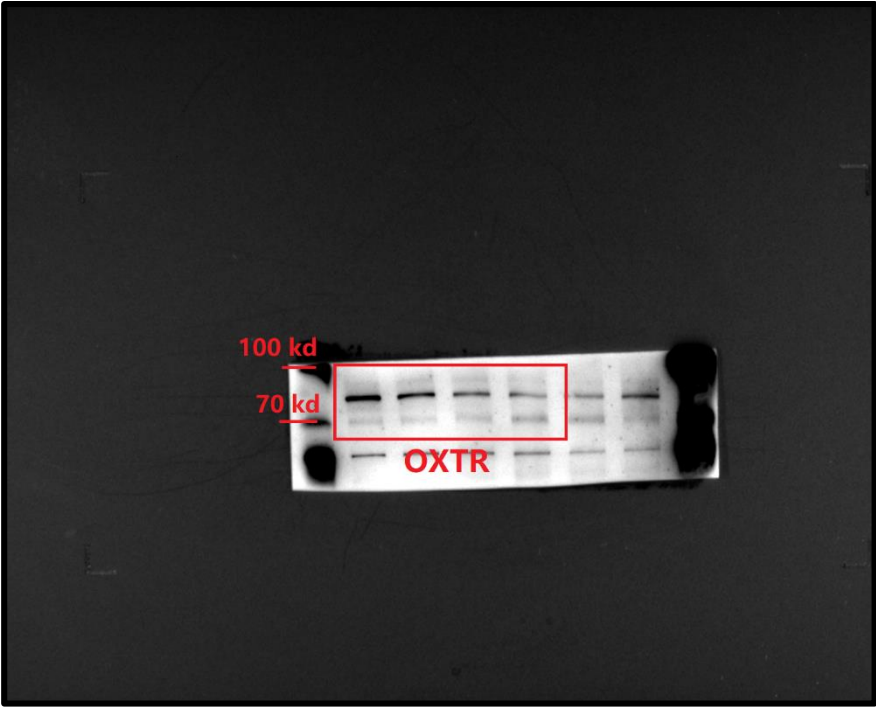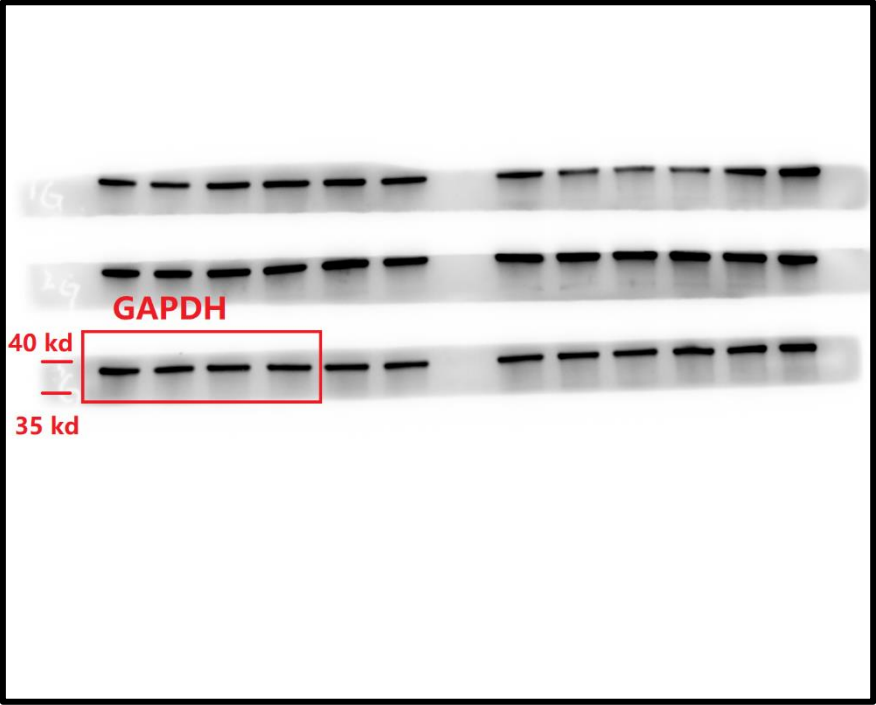

Supplement: Supplementary file 4 — Source Data [file 41467_2022_32787_MOESM4_ESM.zip › Source Data/Supplementary Figure 5/Sup Figure 5l.pdf]

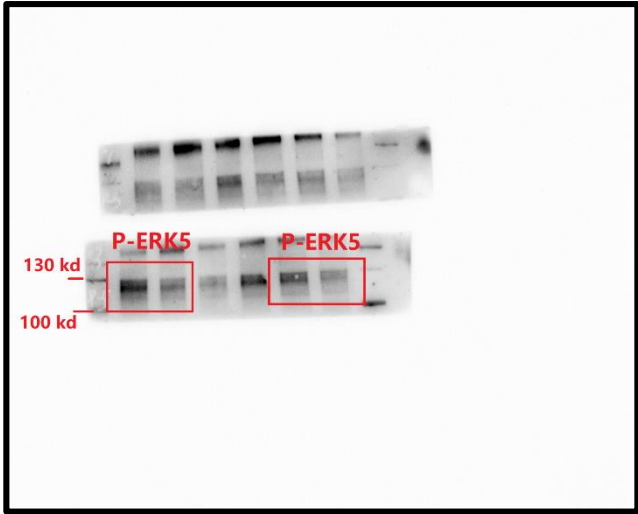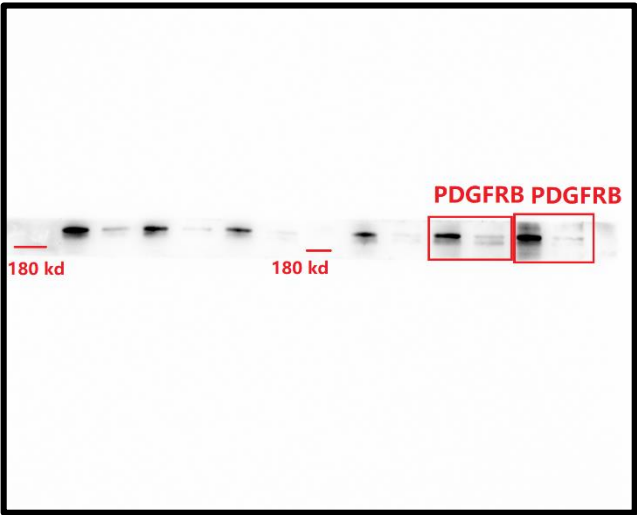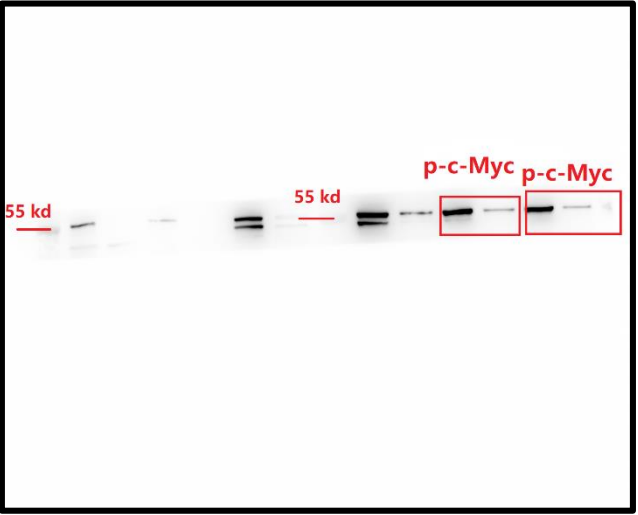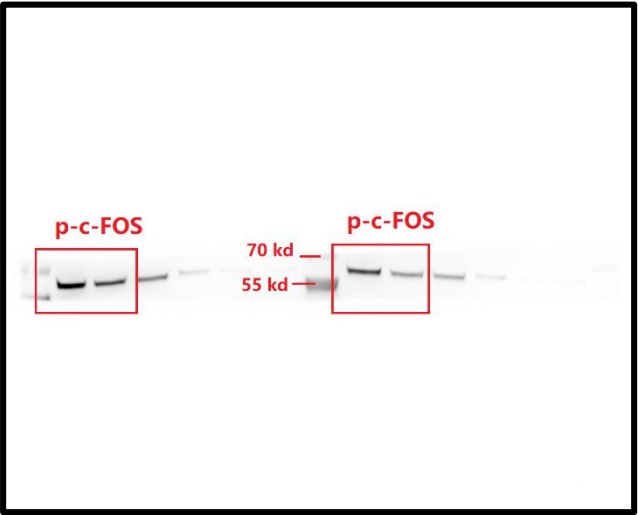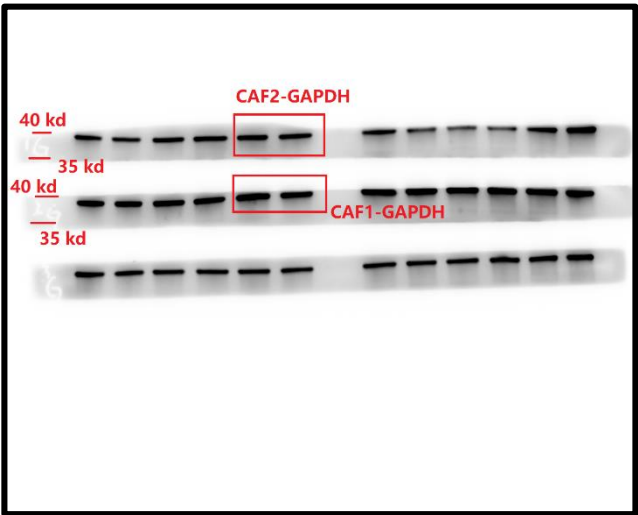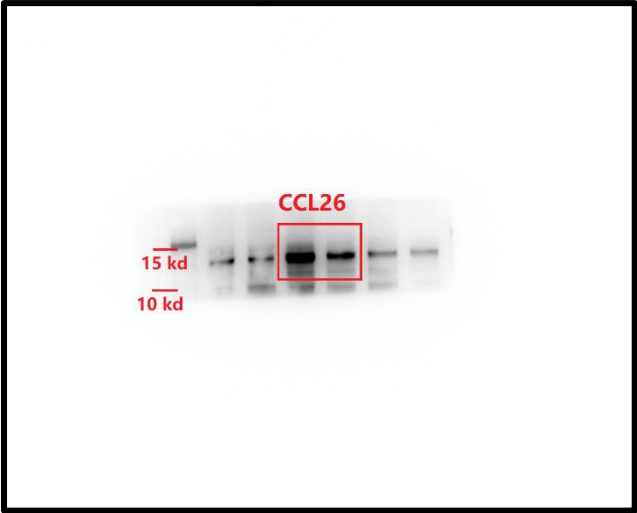

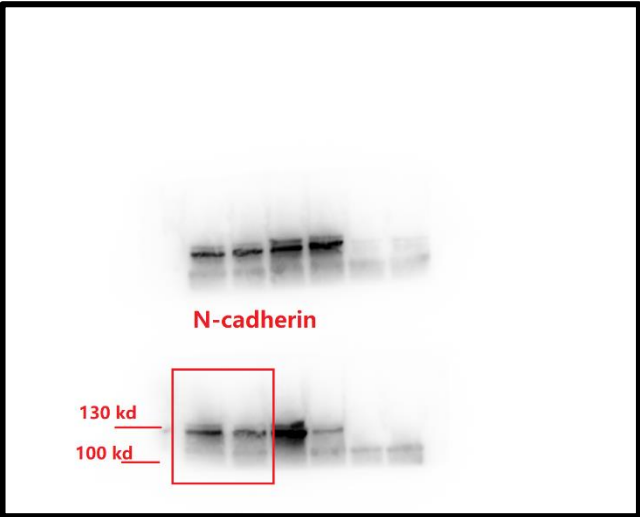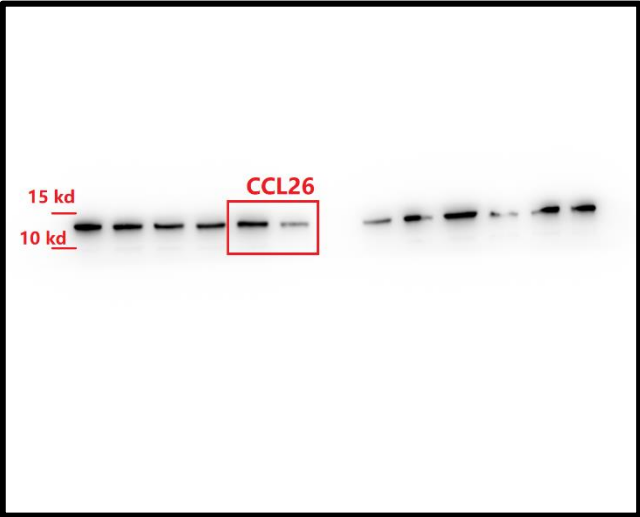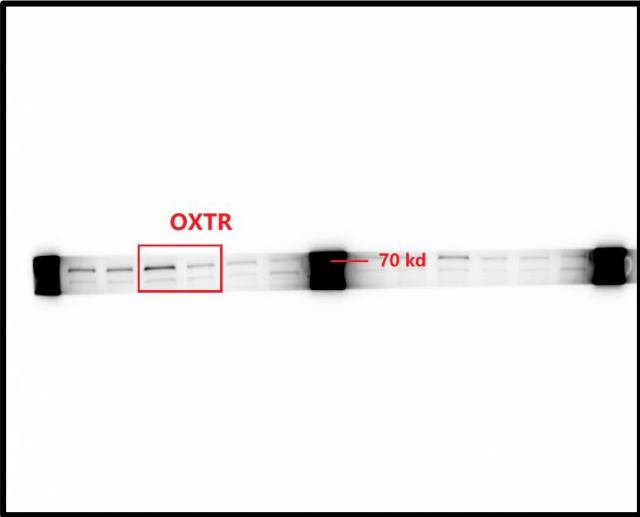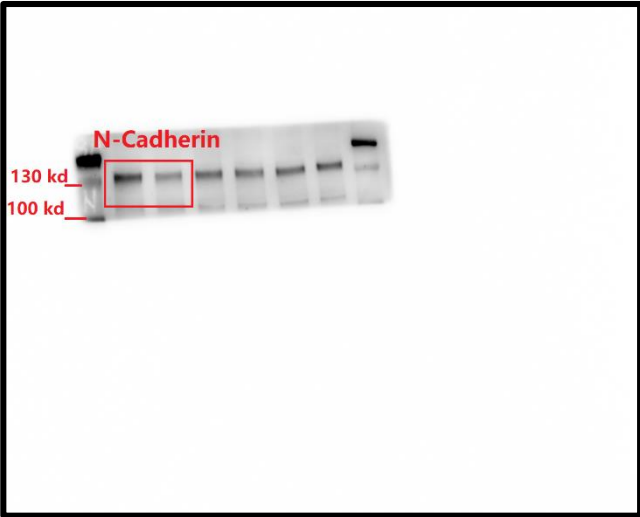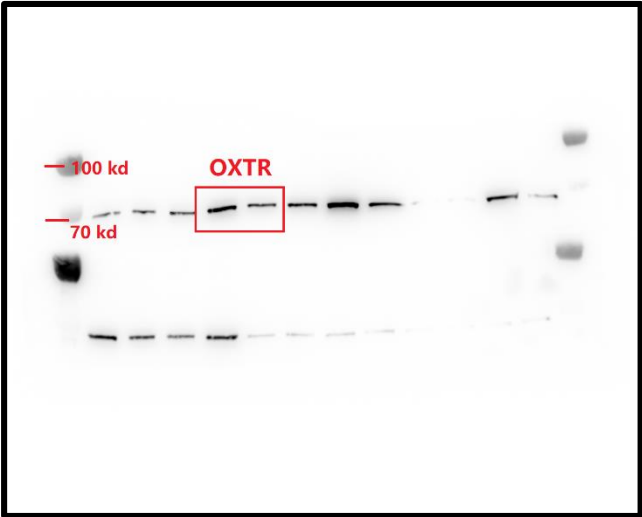

Supplement: Supplementary file 4 — Source Data [file 41467_2022_32787_MOESM4_ESM.zip › Source Data/Supplementary Figure 5/Sup Figure 5o.pdf]

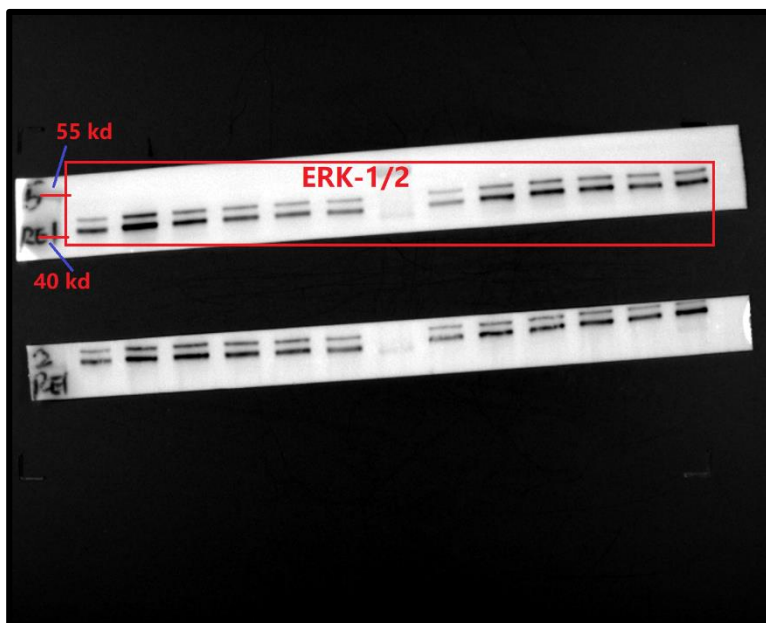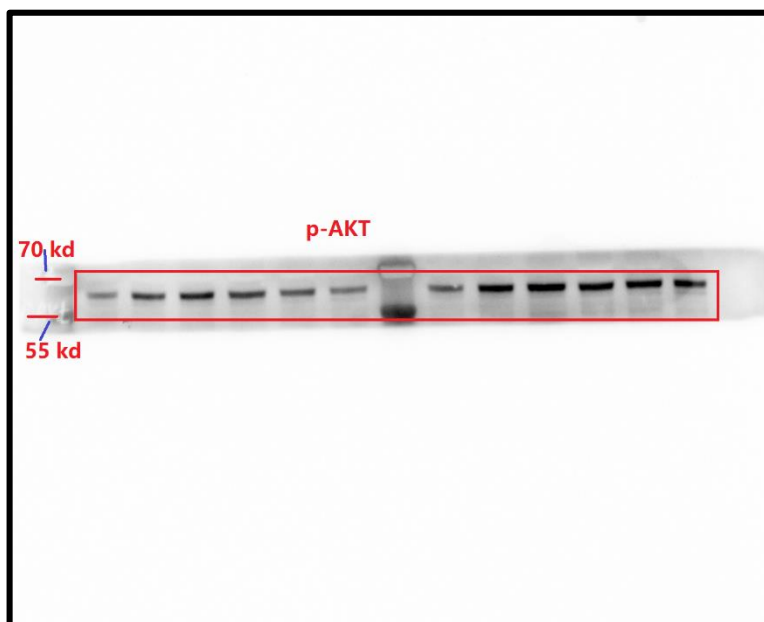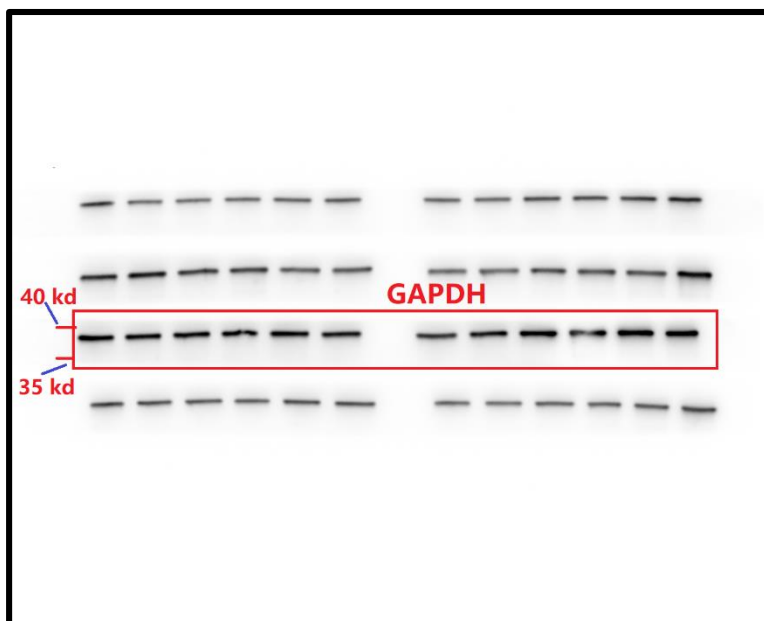

Supplement: Supplementary file 4 — Source Data [file 41467_2022_32787_MOESM4_ESM.zip › Source Data/Supplementary Figure 6/Sup Figure 6h.pdf]
